# Online cognitive monitoring technology for people with Parkinson’s disease and REM sleep behavioural disorder

**DOI:** 10.1038/s41746-024-01124-6

**Published:** 2024-05-07

**Authors:** Maria Bălăeţ, Falah Alhajraf, Tanja Zerenner, Jessica Welch, Jamil Razzaque, Christine Lo, Valentina Giunchiglia, William Trender, Annalaura Lerede, Peter J. Hellyer, Sanjay G. Manohar, Paresh Malhotra, Michele Hu, Adam Hampshire

**Affiliations:** 1 Department of Brain Sciences, Imperial College London, London, UK; 2 Oxford Parkinson’s Disease Centre, Nuffield Department Clinical Neurosciences, University of Oxford, Oxford, UK; 3 Population Health Sciences, University of Bristol, Bristol, UK; 4 Centre for Neuroimaging Sciences, Institute of Psychiatry, Psychology and Neuroscience, King’s College London, London, UK

**Keywords:** Parkinson's disease, Attention

## Abstract

Automated online cognitive assessments are set to revolutionise clinical research and healthcare. However, their applicability for Parkinson’s Disease (PD) and REM Sleep Behavioural Disorder (RBD), a strong PD precursor, is underexplored. Here, we developed an online battery to measure early cognitive changes in PD and RBD. Evaluating 19 candidate tasks showed significant global accuracy deficits in PD (0.65 SD, *p* = 0.003) and RBD (0.45 SD, *p* = 0.027), driven by memory, language, attention and executive underperformance, and global reaction time deficits in PD (0.61 SD, *p* = 0.001). We identified a brief 20-min battery that had sensitivity to deficits across these cognitive domains while being robust to the device used. This battery was more sensitive to early-stage and prodromal deficits than the supervised neuropsychological scales. It also diverged from those scales, capturing additional cognitive factors sensitive to PD and RBD. This technology offers an economical and scalable method for assessing these populations that can complement standard supervised practices.

## Introduction

Parkinson’s disease (PD) is a complex neurological disorder that is primarily defined by its motor symptoms. However, this heterogeneous condition is also associated with a heightened risk of cognitive impairment, which serves as a marker for disease severity^1^ and can progress to PD-dementia^2^. These impairments have significant ramifications for patients and their carers, motivating a focus on developing new technologies to monitor cognitive decline in PD^3^.

A notable advance in our understanding of PD manifestations, including cognitive deficits, comes from the study of rapid eye movement (REM) sleep behaviour disorder (RBD). RBD is a sleep disorder characterised by the acting out of vivid, often unpleasant, dreams with vocal sounds and sudden, violent arm and leg movements during REM sleep. It has been estimated to affect approximately 1% of older adults, which is comparable to PD^4^. Critically, individuals diagnosed with RBD have a heightened risk of PD and related neurodegenerative disorders. This risk is not homogeneous: about 30% of RBD patients develop a neurodegenerative disorder within three years of diagnosis, but specific subpopulations identified through predictive markers have risks as high as 65%^5^. Cognitive impairments have been detected in up to 50% of RBD patients^6^. These heightened risks present an opportunity to investigate cognition across the disease continuum spanning from the RBD prodrome to later stage PD^7^. However, this potential is hindered by the high cost and limited scalability and repeatability of standard face-to-face assessment tools^8^.

Automated online assessment technologies offer a new approach for screening and examining cognitive performance that has advantages over traditional supervised assessment scales^9^. Most notably, reliance on pen-and-paper scales incurs significant costs associated with administration, as well as additional burdens on patients and their carers who must travel for face-to-face appointments. Online testing alleviates these burdens and reduces costs. With careful design, it has the potential to engage participants more effectively through gamification and, crucially, may be repeated many times^10^, enabling large-scale regular longitudinal monitoring accessible from individuals’ homes for research purposes^11^ and as an adjunct to in-person assessments^12^. The advantages of unsupervised online deployment extend to automated scoring and data export, minimising the time individual assessors spend on data management following appointments^12^. Furthermore, modelling of detailed performance timecourses can output model-based and contrast measures of specific aspects of cognitive processing, e.g., subtracting out and quantifying components of performance variance that relate to basic visuomotor slowing^13,14^.

To date, this potential has not been realised for PD, though researchers have begun to develop computerised assessment batteries that are sensitive to cognitive change in this population. Early work applied the Cambridge Cognitive Assessment battery^15^ and the Frontal Assessment Battery^16^. However, these pioneering tools were designed to assess a wide range of patients with varying pathologies and were originally intended for deployment under supervised conditions, placing limits on their repeatability and scalability.

With the advancement of internet-based technologies, cognitive assessment tools have transitioned from bedside/clinical administration to both online-supervised^17^ and unsupervised in-home delivery^18^. Concerning cognition in PD, Hanna-Plady et al. (2010) evaluated the NeuroTrax computerised battery in a study of 50 PD patients with mild to moderate disease severity^19^. However, they concluded that akin to the mini-mental state examination (MMSE), it lacked the necessary sensitivity for assessing PD-specific cognitive deficits. Others have focused on remote screening and monitoring PD-specific motor characteristics, such as gait issues^20^, and cognitive symptomatology, including visuospatial deficits^21^. Despite these advances, few, if any, current tools offer ease of deployment in clinical populations and high sensitivity in detecting subtle cognitive changes at the early-mid stages of PD or in prodromal conditions like RBD. Such a tool could be transformative for early screening of patients and monitoring to identify those at risk of accelerated cognitive decline based on their trajectories^10^ and when validating early-stage cognitive interventions.

When tailoring a cognitive testing battery for RBD and early- to mid-stage PD, several factors merit consideration. First, cognition is multifaceted, encompassing distinct domains including attention, memory, reasoning, and language. PD is heterogeneous, with different patients having different combinations of deficits across these domains that have distinct neurobiological and genetic underpinnings^22,23^. For example, PD-associated cognitive deficits have been reported to include memory, attention, visuospatial abilities, and particularly executive functions, such as mental flexibility, set-shifting, switching, efficient planning of future actions, and problem-solving^1^. Meanwhile, RBD deficits have also been reported in attention and memory domains^24^. An optimal assessment battery should encompass and differentiate between these cognitive domains, i.e., with tasks that have sensitivity to PD or RBD and that are decorrelated from each other to provide detailed multivariate deficit profiles.

Currently, the MMSE and Montreal Cognitive Assessment (MoCA) are amongst the most widely employed cognitive assessment tools in PD, whereas more detailed comprehensive cognitive assessment is often considered too time-consuming for repeat or large-scale deployment^25^. A new battery assessing cognition in PD should be at least as sensitive to PD and RBD as these scales. Indeed, it would be advantageous to outperform them, as they are known to have limited detail across cognitive domains and poor sensitivity to subtle cognitive changes^12^, especially in early-stage PD^26^, whereas computerised assessments can provide more detailed and precise outputs^13^. Consequently, an optimal assessment battery might also capture cognitive domains that are divergent from those scales whilst being affected in early and prodromal PD. Moreover, people, when assessed online, may not tolerate long sessions or tedious tasks. Therefore, the battery should achieve that detail while being engaging and concise, ideally requiring no more than 20–30 min to complete.

In developing an online cognitive assessment battery, it is also essential to consider the potential challenges patients may face, whether due to their condition or unfamiliarity with computerised testing. As such, the battery should feature a user-friendly interface, incorporating intuitive tasks and clear instructions. Furthermore, there should be minimal sensitivity to variability in personal devices that participants use to perform the tasks. Differences in screen size and response time recording should be minimised as they can influence the measurement of cognitive performance^27^. Finally, algorithms can be programmed that generate novel stimuli on the fly, with careful balancing along relevant difficulty dimensions, to minimise cross-session learning effects and enhance the interpretability of longitudinal timecourses from repeat assessments.

Here, we sought to develop such an assessment battery. Specifically, we objectively assessed the cognitive performance of control, PD and RBD participants from the Oxford Discovery Cohort^28^ using a superset of 19 candidate tasks spanning cognitive domains previously reported to be affected in these populations. The tasks were selected from the broad library available on the Cognition online assessment platform, which has been successfully deployed in diverse clinical and non-clinical population studies with >500,000 participants^12,29–32^. First, we identified tasks that were (a) sensitive to cognitive deficits in PD and RBD, (b) insensitive to device-related confounds, and (c) decorrelated in relation to each other within a data-driven factor model of cognitive domains. Then, we evaluated the convergence and divergence of online task scores with the Montreal Cognitive Assessment (MoCA) to determine whether they captured additional cognitive constructs that were sensitive to subtle early cognitive changes in these populations. Based on these results, we proposed a brief assessment battery with optimal properties for research and clinical application in early-stage and prodromal PD.

## Results

### Clinical characteristics and cognitive assessment engagement

Demographics and numbers of participants completing each task are reported in Table 1 and Supplementary Table S2. There was no significant difference in the average education levels of patient groups and controls. There was a difference in mean ages at both clinical and cognitive assessment time points, with controls being, on average, half a decade older than the patient population. Also, as expected, given the population distribution of RBD, this group was ~90% male. In line with the RBD state being a prodrome, PD patients had worse scores than RBD patients on the updated Unified Parkinson’s Disease Rating Scale (MDS-UPDPRS) I-III scales, as well as on the Purdue dexterity scale, but not on the REM Sleep Behaviour Disorder Screening Questionnaire (RBDSQ). There was no difference between patients and controls MoCA or MMSE scores at baseline or at their most recent clinical assessment.

**Table 1:** Clinical and demographic characteristics of participants at baseline (at the point of recruitment into the Oxford Discovery Cohort) and the clinical visit closest to the cognitive assessment

	Control Baseline	Control Assessment	PD Baseline	PD Assessment	RBD Baseline	RBD Assessment	Significant baseline differences	Significant differences at assessment
Age at cognitive assessment	N/A	72.84 ± 8.37	N/A	66.01 ± 8.59	N/A	68.67 ± 8.78	N/A	***
Age at clinical assessment	64.97 ± 8.22	71.66 ± 8.6	58.2 ± 8.83	65.24 ± 8.79	64.39 ± 8.39	68.09 ± 8.66	***	***
MOCA total	27.11 ± 1.86	N/A	27.21 ± 1.66	27.45 ± 2.19	26.35 ± 2	26.69 ± 1.7	ns	ns
MMSE total	28.73 ± 1.48	N/A	28.79 ± 1.42	N/A	28.28 ± 1.17	28.5 ± 1.15	ns	ns
Purdue total	38.55 ± 6.26	N/A	34.48 ± 6.81	29.56 ± 7.86	35.7 ± 6.05	32.32 ± 5.87	***	*
Purdue assembly	25.68 ± 6.52	N/A	23.1 ± 6.27	19.2 ± 5.9	22.26 ± 5.61	21.39 ± 5.34	*	***
Hoehn&Yahr	N/A	N/A	1.61 ± 0.56	2 ± 0.47	0	0.06 ± 0.31	***	***
MDS-UPDRS I	3.80 ± 2.49	4.90 ± 4.74	8.13 ± 4.62	9.78 ± 5.13	6.53 ± 4.16	7.17 ± 4.61	*	***
MDS-UPDRS II	0	0.90 ± 1.49	6.9 ± 5.83	11.64 ± 7.34	1.73 ± 2.43	2.69 ± 4.15	***	***
MDS-UPDRS III	1.43 ± 1.64	N/A	22.85 ± 11.49	30.67 ± 9.87	3.63 ± 2.91	6.83 ± 6.97	***	***
MDS-UPDRS IV	N/A	N/A	0.7 ± 2.42	4.1 ± 3.64	N/A	N/A	N/A	N/A
Probability of idiopathic PD	N/A	N/A	90.49 ± 3.62	95.36 ± 5.67	N/A	N/A	N/A	N/A
ESS scale	5.63 ± 3.63	5.53 ± 3.2	6.93 ± 4.56	7.66 ± 4.24	5.95 ± 4.24	5.61 ± 3.89	ns	*
RBDSQ scale	2.54 ± 1.81	2.15 ± 1.77	4.26 ± 2.82	5.04 ± 3.14	9.93 ± 2	9.45 ± 2.48	*	***
Age at PD/RBD diagnosis	N/A	N/A	56.95 ± 9.09	N/A	63.23 ± 8.63	N/A	***	N/A
Age at motoric symptom	N/A	N/A	55.18 ± 9.4	N/A	58.83 ± 9.98	N/A	N/A	N/A
Disease duration since motoric symptom onset in years at the latest clinical assessment	N/A	N/A	3.02 ± 2.28	10.06 ± 2.59	N/A	N/A	N/A	N/A
Disease duration since diagnosis in years at the latest clinical assessment	N/A	N/A	1.27 ± 1.52	8.329 ± 1.9	1.17 ± 1.71	4.86 ± 3.31	N/A	N/A

Means ± SD are presented for all scores, for all participants who took part in the study: *N* = 50 Healthy Controls (42% female; 4.95 ± 2.34 years of further education), *N* = 59PD patients (48% female; 4.2 ± 2.77 years of further education), *N* = 54 RBD patients (11% female; 4.35 ± 2.5 years of further education). There was no significant difference between participants years of further education. Participant ethnicity was 98% white background. ANOVA and *t*-tests results significance: ns for not significant, * for *p* < 0.05; ** for *p* < 0.01; *** for *p* < 0.001. N/A indicates where data were not collected or the statistical test was not applicable.

### PD, RBD and age-related decline have distinct patterns of cognitive deficit

**Fig. 1:**
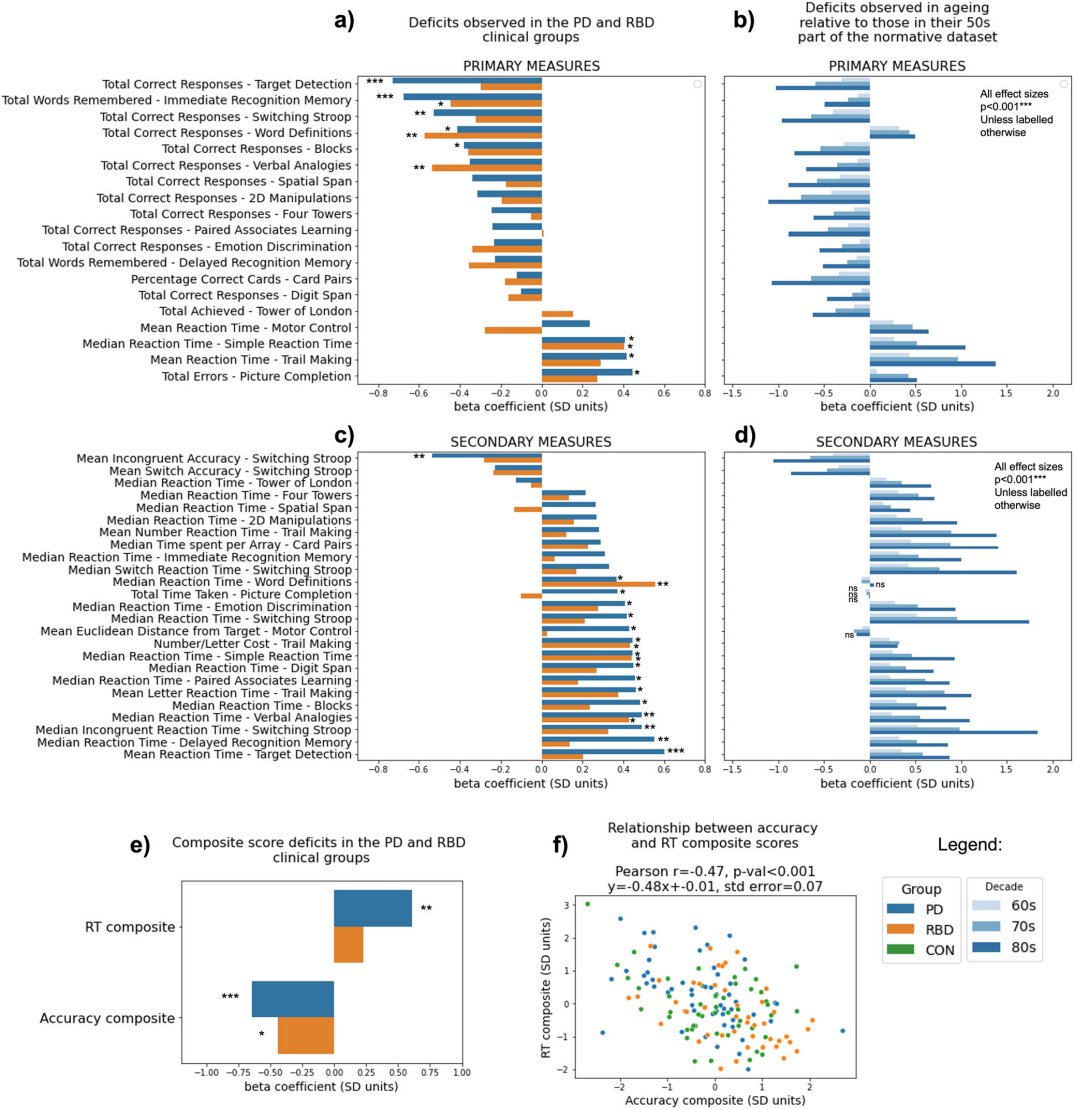
Assessing cognition with the reduced battery in patients at group levels, as well as individually. **a** Deficits in primary task measures identified in PD and RBD groups relative to the controls from the Discovery Cohort. **b** Deficits in primary task measures characteristic to older age decades relative to those in their 50 s in the large independent population sample. **c** Deficits in secondary task measures identified in PD and RBD groups relative to the controls from the Discovery Cohort. **d** Deficits in secondary task measures characteristic to older age decades relative to those in their 50 s in the large independent population sample. **e** Differences in composite scores in the patients who completed all tasks of the optimised battery relative to controls. **f** Speed accuracy trade-off relationship between composite scores. ANOVA results significance: * for *p* < 0.05; ** for *p* < 0.01; *** for *p* < 0.001. N/A indicates where data was not collected. Task outcome measures are further characterised in Supplementary Materials–Supplementary Task Descriptions and Supplementary Table 1.

R-squared of models adjusting sociodemographic confounds showed that they accounted for a substantial proportion of variance in task performance (R-squared values range up to ~0.3). When using linear modelling to assess differences between patients and controls (Fig. 1) for the adjusted primary task scores, both PD and RBD showed deficits in immediate and delayed recognition memory, word definitions and verbal analogies. Additional deficits were evident in the PD cohort, including Target Detection, Switching Stroop and Trail Making. For secondary scores, PD patients consistently took longer to respond across multiple tasks, whereas RBD patients showed more selective impairments, notably in simple reaction time tasks and Trail Making. Contrasting directly between the two patient groups showed superior performance for RBD patients in Spatial Span, Motor Control, and Picture Completion (Supplementary Materials–Supplementary Discussion).

For comparison, sensitivities of the same task measures were analysed in relation to age decade in the large independent online cohort, contrasting people who were in their 80s, 70s and 60s relative to those in their 50s (Fig. 1b) and Fig. 1d), participant N per age decade in Supplementary Table 15, model outputs in Supplementary Materials – Supplementary Model Outputs). The pattern of cognitive differences was distinct from that of PD or RBD. A prominent example is the performance on the Word Definitions task, where healthy participants improve with age, as expected for a measure of crystallised intelligence, whereas both PD and RBD show significant deficits relative to controls.

Finally, both patient groups had overall poorer performance for the global composite calculated via factor analysis across all task summary scores. PD but not RBD patients had poorer performance on the global response time composite than healthy controls. The difference between RBD and PD was non-significant.

### Discriminability of primary task outcomes to patient groups vs. device

**Fig. 2:**
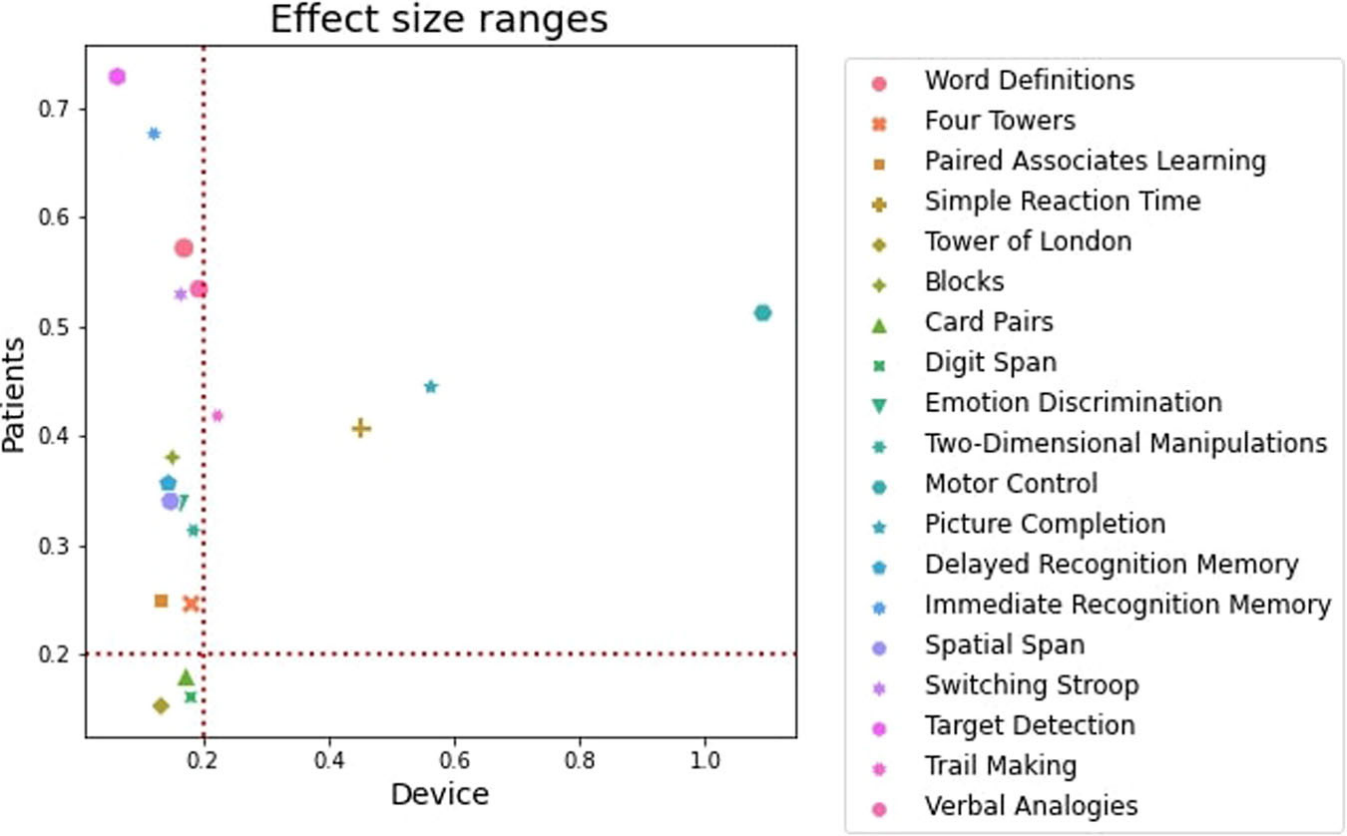
Task discriminability analysis. Effect size ranges in SD units of differences in performance for clinical groups in the Discovery Cohort (*y*-axis) and mobile vs. computer devices for preexisting participants from the Cognitron platform (*x*-axis) (Supplementary Table 2). The tasks in the top left quadrant are optimal, with medium to high discrimination of patient groups and small to negligible discrimination of devices. The lower left quadrant represented tasks with minimal discriminability to both group differences and devices. Right-hand quadrants have suboptimal device discriminability.

Before calculating the ranges of effect size differences between patient vs. control groups and assessment devices, we first accounted for potential confounding effects of population factors, specifically, age-decade, gender, and education among the patients (Fig. 2). Tower of London and Card Pairs were excluded from the final battery, as they had low discrimination of patient groups. SRT, Motor Control and Picture Completion were excluded from the final battery due to higher device sensitivity.

### Latent factors captured by the primary measures of the 19 candidate tasks

**Fig. 3:**
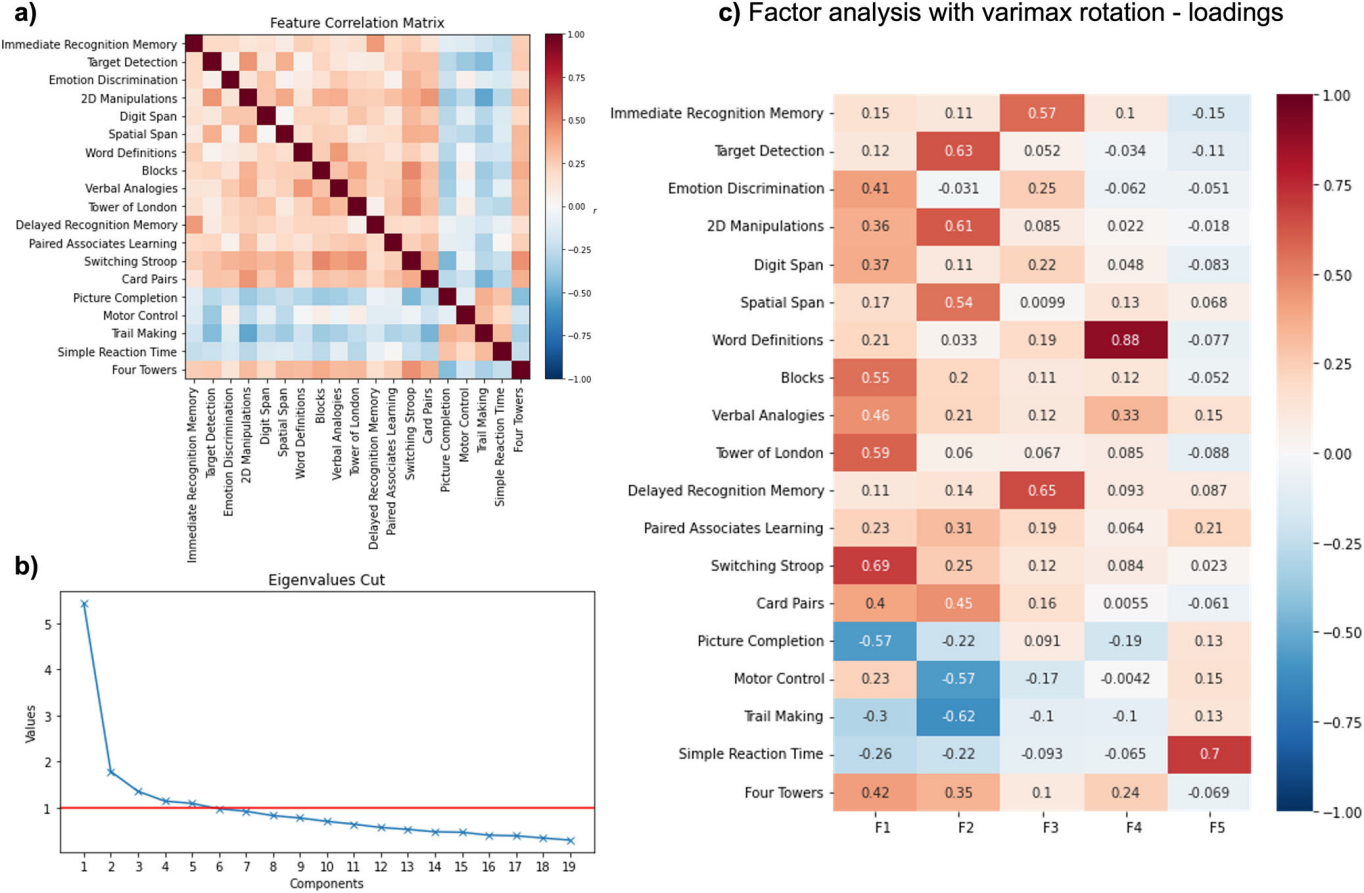
Factor analysis. **a** The correlation between all primary task performance scores after standardisation. Note that Trail Making, Motor Control and Simple Reaction Time are reaction time measures. **b** Scree plot of Eigenvalues. **c** Task loadings from factor analysis with orthogonal varimax rotation of the task summary scores from controls and the PD and RBD patients. We labelled F1 as executive functions, F2 as visuospatial/attention functions, F3 as short-term memory, F4 as word knowledge and F5 as motor reaction time.

Applying the Kaiser convention to the eigenvalues identified a five-factor solution as optimal (Fig. 3b)). Applying factor analysis with varimax rotation resulted in interpretable task-factor loadings, specifically, visuospatial ability, executive function, short-term memory, word knowledge, and motor reaction time (Fig. 3c)). Notably, the most discriminative tasks for PD and RBD loaded onto different factors, which can therefore enable multivariate profiling of cognitive deficits. SRT loaded onto the reaction time factor, but since it has high discrimination of devices, we did not retain this task. This is not problematic, as reaction times are measured for all tasks. We also excluded paired associate learning (PAL) from the recommended battery as it did not load strongly or discretely on any of the factors and shares variances with other tasks that better discriminate PD and RBD from controls. (Full task selection process in Supplementary Table 4).

### Benchmarking against the MoCA

**Fig. 4:**
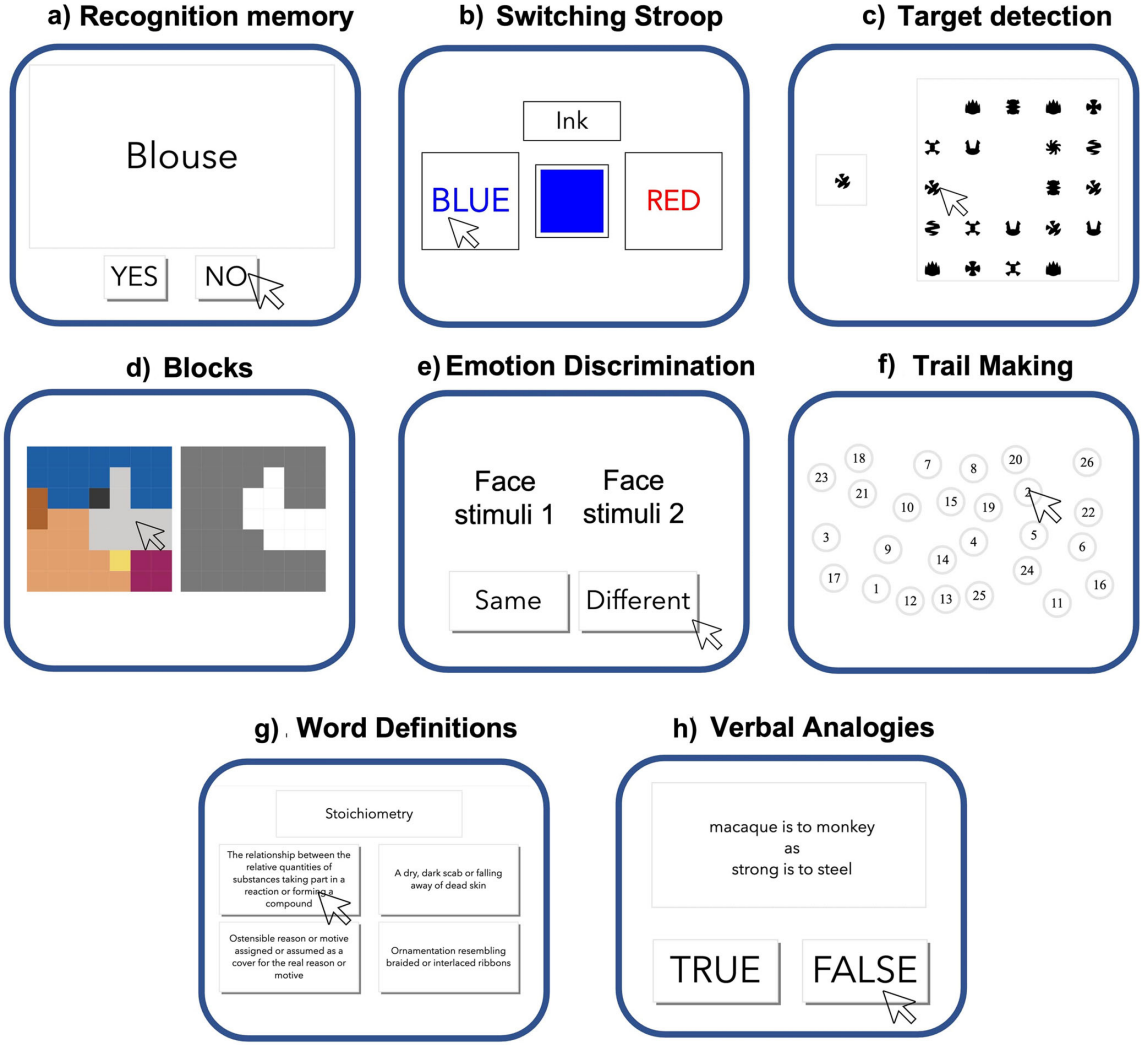
A short comprehensive cognitive assessment battery. **a** Word recognition memory immediate and delayed (1 min for immediate presentation, 30 s for delayed presentation)—participants are presented with a list of words at the start and then asked whether they recognise them as being from the list immediately after completion of all other tasks. **b** Switching Stroop (1 min 30 s)—participants indicate the colour of either the ink or the colour of the text, which can be congruent or incongruent, with the relevant dimension periodically switching. **c** Target Detection (2 min)—participants identify the target in a pool of similar-looking targets that are periodically updated on the screen. **d** Blocks (1 min)—participants delete shapes on the left panel to match the structure on the right panel, with blocks falling under gravity when unsupported from below. **e** Emotional discrimination (1 min 30 s)—participants indicate whether facial emotions displayed by the models are identical or different from one another. **f** Trail Making (2 min)—participants click on numbered circles in ascending order as quickly as possible. A further condition requires clicking on numbers and letters alternatively in ascending order. **g** Word definitions (4 min)—participants indicate which definition is correct for a sequence of rare words. **h** Verbal analogies (3 min)—participants indicate whether the relationships between two pairs of words are analogous, solving as many problems as possible within the time limit. The whole battery takes ~20 min and does not require supervision.

**Fig. 5:**
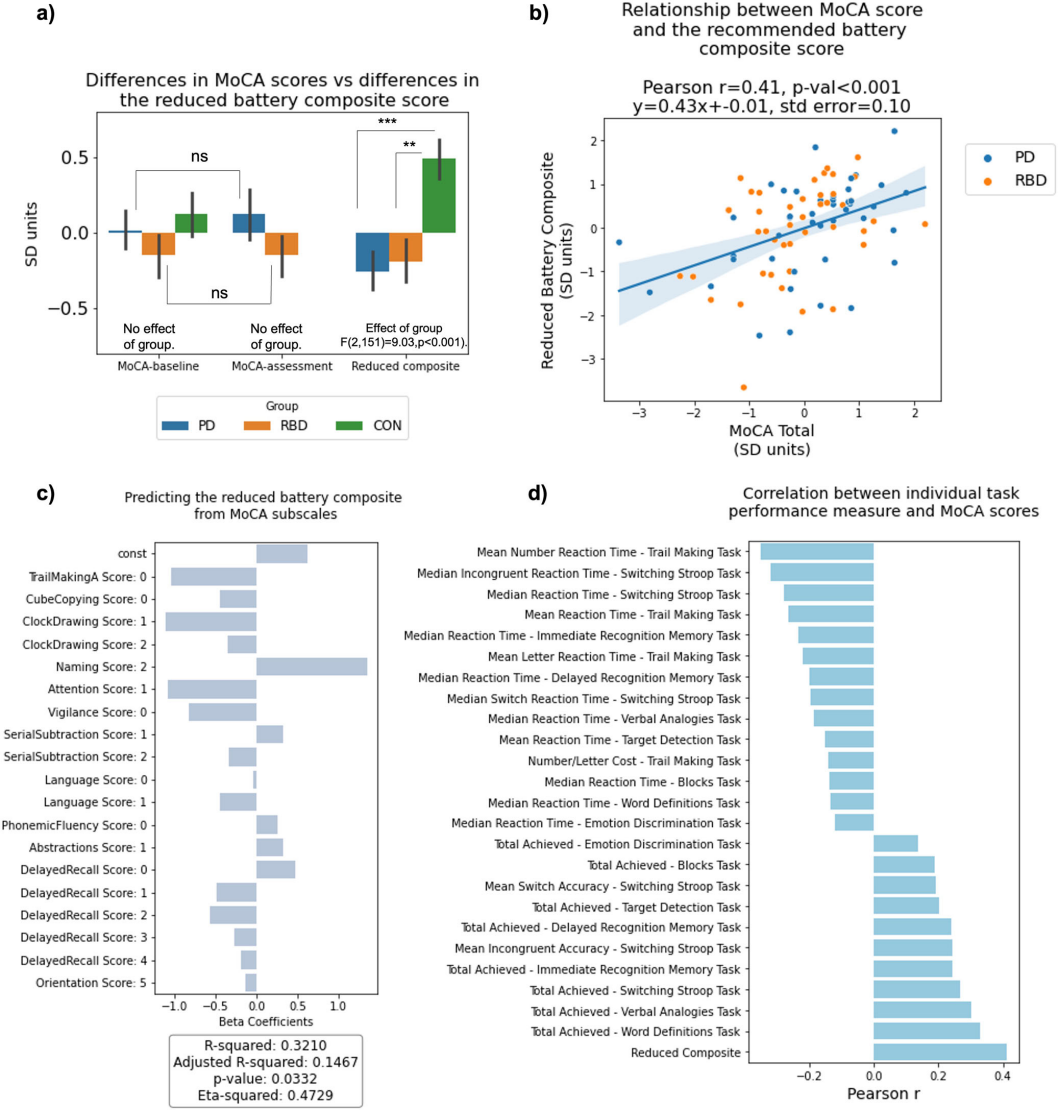
Relationship between MoCA and performance on the computerised cognitive tasks. **a** Differences in MoCA scores across groups at baseline and at the timepoint closest to the online cognitive assessment, and for online cognitive assessment global composite score. Note the online assessment detects group differences through a range that the MoCA is insensitive to. **b** The relationship between the MoCA scores and the global performance score on the selected battery of tasks. The moderate correlation indicates a convergence between these assessments that is equivalent to the re-test correlation across MoCA time points. **c** Predicting the reduced battery composite score from MoCA subscales. **d** Pearson’s correlations between the MoCA scores after adjusting for age decade, sex and years of further education and performance on individual computerised tasks part of the final selection. Note some tasks have a low correlation with MoCA but detect cross-group differences, indicating divergence from constructs measured by the MoCA.

The proposed online assessment battery (Fig. 4) was contrasted against the MoCA scale, which is routinely used under supervised conditions to assess cognition in PD and RBD patients. Multiple linear regression was fitted to predict the standardised MoCA score, corrected for effects of age-decade, sex, and level of education, from the performance of the selected tasks when processed in the same way (Supplementary Table 7). Online task performance showed a moderate but significant correlation with MoCA score with moderate accuracy (*R*^2^ ~ 0.25, *p* < 0.01). Furthermore, there was a medium-scaled correlation between the reduced battery composite score (determined as the first factor in a factor analysis of the reduced battery data) and the global MoCA scores (*r* = 0.41, *p* < 0.001) (Fig. 5b)). For comparison, comparing the MoCA scores at baseline with those from the most recent MoCA assessment produced a similar correlation (*r* = 0.43, *p* < 0.001). Applying a linear model with MoCA subscales to predict composite score from the recommended online battery produced a significant fit (*R*^2^ ~ 0.3, *p* = 0.03) (Fig. 5c)). The basis of the moderate correlation was apparent when examined at a finer grain; some online tasks exhibited only small correlations with MoCA score despite having high discriminability for RBD and PD vs. controls, indicating that they evaluate additional aspects of cognitive performance that are affected in these clinical populations (Fig. 5d)). While Word Definitions, a measure of crystallised intelligence, was the task where performance was most highly correlated with the total MoCA score, tasks such as Blocks and Emotional Discrimination had small correlations. Secondary measures also had notable (inverse) correlations with the MoCA score, the highest being for the Number Reaction Time when performing on the Trail Making task, whereas the switching cost for the Trail Making task and the median reaction time for Blocks had the lowest correlations. In contrast to the computerised assessment battery as reported above, discriminability of the MoCA to cognitive deficits in RBD and early-mid stage PD was of small effect size (mean difference = 0.32 SD, *p* = 0.1) and statistically non-significant. Paired *t*-tests showed that patient scores did not significantly change in the time (mean 6.5 years ± 2.57 SD) from baseline until the most recent assessment for PD patients (*T* = −7.67, *p* = 0.4) or RBD patients (*T* = −0.38, *p* = 0.7).

### Sensitivity analyses

Running variants on the analysis with sociodemographic factors accounted for within the same model as the group predictor and where age was treated as a continuous not categorical variable, did not materially change the results (Supplementary Table 8, Supplementary Fig. 5, Supplementary Table 9, Supplementary Table 10, Supplementary Table 13, Supplementary Table 14, Supplementary Figure 6, Supplementary Table 11, Supplementary Table 12, Supplementary Table 13, Supplementary Table 14). Assessing the generalisability of these results using leave-one-out and cross-validation methods showed modest but statistically significant correlations between predicted and observed accuracy and reaction time composites. Mean squared error calculations indicated that the predicted values were close to the observed values (Supplementary Fig. 13, Supplementary Fig. 14).

Contrasting PD with (*N* = 15) vs. without probable RBD (*N* = 44) showed the RBD sub-group to have overall worse response times, with the accuracy composite also showing a non-significant trend towards poorer performance (Supplementary Fig. 7, Supplementary Table 15, and Supplementary Table 16). Contrasting participants with PD and probable RBD vs. those with idiopathic RBD also showed overall worse response times for the PD group (Supplementary Fig. 8, Supplementary Table 16, and Supplementary Table 17). Contrasting these three groups individually to controls showed that participants with PD and probable RBD had the worst deficits, particularly in the reaction time measures (Supplementary Fig. 9, Supplementary Table 19, Supplementary Table 20).

To explore how bradykinesia in the PD group might affect PD participants task responses, we ran correlations between UPDRS-III score (general motor deficits), and a more focused composite of finger tapping and the hand movement sub-scales, with task performance. This showed significant correlations in the medium range with the final battery composite score but not general accuracy or reaction time composites taken across the entire task performance (Supplementary Materials Fig. 10).

When the proposed battery composite score was correlated with the MMSE as measured at enrolment (i.e., with a mean 10-year offset from the online assessment) it showed broadly similar results to those reported above for the MoCA (Supplementary Figure 6 and Supplementary Table 21).

## Discussion

The results of this study confirmed the suitability of online technology for assessing cognitive functions in PD and RBD. We identified a subset of tasks that produced performance scores with the intended optimal properties, i.e., (a) discriminating patients from controls, (b) having minimal performance variability attributable to the device used during the assessment, (c) being brief (20-min) and (d) producing measures across multiple relevant cognitive domains.

The strength of the correlation between the online battery global composite score and the MoCA was comparable to the MoCA retest reliability across time points, but the online composite also detected subtle cognitive deficits in both PD and RBD groups, whereas the MoCA did not. These global cognitive differences relative to controls had medium-large effect sizes for PD and were of an equivalent scale to approximately 20–30 years of age beyond 50 for the RBD group. This indicates that the online tasks were substantially more sensitive to subtle cognitive changes. Relatedly, the MoCA did not detect significant changes between the baseline and the closest time to the online cognitive assessment.

At the individual task level, online assessment scores with high discriminability to PD and RBD displayed varied correlations with MoCA scores and subscales. This reflects that the online battery measured additional constructs to the MoCA or, in some instances, the same constructs at higher precision. The latter was exemplified by immediate memory for words; despite both assessments featuring word memory tasks, only the online task discriminated PD and RBD from controls. This disparity was likely due to the MoCA version’s short word list (five) making it susceptible to ceiling effects.

More broadly, the observation of superior patient-group discrimination aligned with studies applying online assessment tasks in other clinical populations, e.g., Multiple Sclerosis^11^, dementia^29^, traumatic brain injury^10,12^, COVID-19^30^ and autoimmune limbic encephalitis^33^. Taken together, the achieved accessibility, heightened discriminability, and superior assessment detail indicate potential value for evaluating early-stage and prodromal patients, enabling the detection of subtle cognitive changes and measuring early-stage intervention effects.

Past studies have presented alternative perspectives on whether RBD and early-stage PD share similar cognitive impairments^24,34–37^, with the concept of a distinct cognitive profile for individuals transitioning from RBD to PD being debated^35^. While intuitively, RBD as a prodrome might be expected to exhibit less severe cognitive deficits than those observed after PD phenoconversion, studies have indicated that individuals who transition from RBD to PD tend to experience more significant cognitive deficits^35^.

Regarding accuracy, we observed that both PD and RBD patients had significant deficits in global cognitive scores and a similar pattern of more pronounced impairments in memory, reasoning, and crystallised intelligence tasks. While PD patients demonstrated statistically significant impairments on a greater number of tasks, sub-threshold trends were discernible in RBD, and the differences between the two patient groups were generally non-significant. This pattern was also consistent with prior research, where cognitive deficits have been reported in both PD and RBD, often preceding the onset of other symptoms that characterise more advanced disease stages^1,6^ and spanning domains, including memory, executive function, attention, and language^36^.

The response time measures showed substantial global impairments in reaction times for PD, aligning with the population’s characteristic motor deficits^38^. Some sub-threshold slowing was also evident in the RBD group, but this did not approach statistical significance. However, when subdividing the PD group into those with and without RBD, the PD-RBD subgroup had more pronounced cognitive deficits, including significantly greater slowing of response and numerically lower performance accuracy. This finding accords with prior research reporting a more aggressive disease trajectory for the RBD sub-type characterised by rapid cognitive decline and risk of progression to dementia^39–42^.

Overall, the profile of cognitive differences associated with PD and RBD differed from that seen for normal ageing. An intriguing example was the significant impairment in the performance of both patient groups on Word Definitions, a measure of crystallised intelligence reflecting the acquisition of words throughout the lifespan. This contrasts with healthy ageing, where individuals, on average, continue to enhance their word knowledge well beyond the age of 50. These findings gain significance, considering the early onset of RBD, which can occur between 38 and 64 years^43^. Given the high probability of phenoconversion to PD or related neurodegenerative diseases^5^, these results align with the perspective that RBD and PD undergo an accelerated decline in both fluid and crystallised cognitive abilities, diverging from the impact of normal ageing processes.

In the largest study to date, involving 754 longitudinally assessed RBD patients, reduced attention and executive function, particularly in the Trail Making Test Part B, were identified as the strongest indicators of future conversion to Dementia with Lewy Bodies (DLB)^44^. Our proposed short comprehensive cognitive testing battery retains attention and executive tasks, including Trail Making, Switching Stroop, and Target Detection. Past findings also highlight the heterogeneity of cognitive deficits, including across PD subtypes^22^. Given the high discriminability of the assessment battery to RBD, early-stage PD, and age-related changes, combined with the detailed multi-domain output, it may be particularly useful for application in future research tracking heterogeneity of cognitive decline spanning the continuum from prodromal to mid-stage PD, and to PD dementia.

Assessing cognition in PD poses a challenge due to the confounding effects of primary motor deficits—slower and less accurate responses may reflect impaired cognitive information processing speeds or motor processes^45^. Indeed, historically, most online digital tools developed for PD have focused on motor tracking rather than cognitive assessment^46–48^. Additionally, cognitive testing tools available for neurodegenerative disorders have often been generalised scales used across various conditions rather than specifically tailored for PD^19^. Here, although there was a relationship between the global cognitive composite and clinically assessed motor deficits, it was possible to achieve an accuracy composite where that correlation was statistically non-significant. To further address motor confounding, future studies may explore the application of newly developed methods specifically designed to disentangle visuomotor and cognitive difficulty-related components of task performance by modelling the detailed trial-by-trial performance timecourses that are recorded by computerised tasks^13^.

The recommended 20-min online battery has several further advantages over popular assessment scales. It is designed to be deployed online without supervision, offering benefits in convenience, scalability and affordability. The tasks feature bespoke and adaptive functions, generating stimuli dynamically on the fly to prevent participants from becoming familiar with specific solutions or response sequences, thereby minimising repeat assessment learning effects^9^. Coupled with the low resource requirement for unsupervised deployment, this technology can enable more precise temporal monitoring of patients. It holds potential for applications in monitoring cognitive decline and conducting better-powered studies of intervention effects and daily variability, especially regarding the relationship between sleep disturbance and cognitive decline in conditions like RBD and PD. Longitudinal monitoring using similar online technology has demonstrated increased sensitivity in tracking cognitive decline in older adults with mild cognitive impairment and early-stage dementia^49^. Further mitigation of learning effects, including general familiarity with online testing, is possible through protocols involving training sessions before longitudinal data collection^49^. Future efforts could develop analogous longitudinal protocols for application in PD and RBD.

The task accuracy scores showed low to negligible scaled sensitivity to the type of device that the assessment was deployed on. This is important due to the diverse array of personal computers, tablets, and smartphones with varying interfaces and software configurations that people commonly own; device sensitivity would necessitate periodic updates of normative data and models to accommodate the changing landscape of available devices. The ability to assess patients on a variety of home devices they own without supervision facilitates cost-effective evaluation on a very large scale and is critical to reducing confounding changes in their abilities with changes in their device when monitoring patients longitudinally.

This study has limitations. Patients without access to compatible devices for the assessment will have been excluded from participation. Notably, though, the software functions on various personal devices, such as computers, smartphones, or tablets with internet access, which are increasingly prevalent in older adult populations^48^. Nonetheless, caution is warranted in extrapolating response rates to the broader PD population as the participants were part of a well-established longitudinal cohort who are more accustomed to cognitive assessments. More broadly, our recent work using the same online assessment technology in healthcare has relevance to the related issues of digital poverty and technology familiarity. There, we implemented a practical solution whereby the same assessment software was deployed in the clinic before routine appointments for those who had not yet participated online; comparable results were achieved remotely and in clinic^12^, thereby maximising efficiency while ensuring inclusivity. The cohort predominantly comprises a white British sample from the Discovery Cohort, necessitating further investigation into the generalisability of findings across culturally diverse PD populations. Despite this, the assessment platform’s previous testing on diverse populations^29^ positions it well for facilitating large-scale studies on cultural variability in cognitive outcomes across different patient groups. This study was cross-sectional; as noted above, longitudinal validation is required for PD and RBD. Although the MoCA has been widely adopted for its brevity, it was originally developed as a quick and effective instrument for detecting mild cognitive impairment. As a benchmark, it likely lacks the discriminability of more detailed supervised assessment scales, especially to subtle changes that occur in early and prodromal stages of cognitive decline. Future research should undertake comparative analyses of online technologies with more complete neuropsychological assessments in PD and RBD. Finally, although the outputs of the online assessment were detailed, the size of the patient populations examined here was too small to leverage that detail with more sophisticated multivariate analysis methods. Future work at a larger population scale could apply machine learning techniques to better characterise cognitive deficits across patient subtypes and predict trajectories.

In summary, our results indicate that automated online assessment technology is a viable method for objectively assessing cognitive deficits in PD and RBD populations remotely without supervision. It demonstrates superior discriminability and captures a broader range of domains compared to the commonly used MoCA and MMSE scales. Considering the aspects of accessibility, discriminability to clinical conditions, insensitivity to device, and factor structure, we propose a subset of tasks suitable for inclusion in a brief and versatile battery for these populations. With further longitudinal validation, this battery could be suitable for tracking disease progression and phenoconversion, studying patient cognitive heterogeneity, and measuring responsiveness to interventions, including at early and prodromal stages.

## Methodology

### Participants

We recruited participants from the Oxford Discovery Cohort^28^, which comprises controls and patients within 3.5 years of diagnosis between 2010 and 2016. The cohort had been assessed every 18 months on clinical scales including the Montreal Cognitive Assessment (MoCA), and on the MMSE at baseline. Between 2020 and 2022, we emailed invitations to participants from this cohort who had a MoCA score above 24 (indicating a lack of global cognitive impairment) at their most recent clinical assessment to complete two batteries of computerised cognitive tasks hosted on the Cognitron platform^29^. Fifty-nine participants with clinically confirmed PD, 54 with isolated RBD and 50 healthy controls completed at least one task, with 56 PD, 50 RBD and 46 healthy controls completing all 19 tasks. Additional normative cognitive data were available via the Cognitron platform and had been collected between December 2019–May 2020 via a custom website https://www.cognitron.co.uk^29^. Ethical approval was given by the South Central- Oxford A Research Ethics Committee in accordance with the Declaration of Helsinki 1964, **Ethics Ref:** 16/SC/0108 and the Imperial College Research Ethics Committee (17IC4009). All participants provided written informed consent for remote cognitive testing as part of their participation in the Oxford Discovery Cohort. Additionally, they gave electronic consent via email prior to completing the Cognition tasks.

### Experimental design

The cognitive tasks were split into two batteries to mitigate fatigue due to long assessment times. Both batteries were available via links hosted on the Cognitron website, which were emailed to participants. Participants were encouraged to complete the batteries on consecutive days. A single battery took approximately 40 min to complete (including reading the instructions for the tasks and inputting their subject ID). The tasks were presented to participants in a fixed order based approximately on increasing complexity, with simple motor and reaction tasks early and more operationally complicated executive tasks later.

Both batteries (Table 2) included a brief questionnaire with five items collecting information about the group, subject ID, and in the case of PD, information about dopaminergic medication, followed by the cognitive tasks. The tasks were designed to measure attention (one task - Target Detection), simple reaction time (one task—SRT), immediate and delayed word recognition memory (two tasks), working memory capacity (four tasks - Digit Span, Spatial Span, Paired Associate Learning and Card Pairs), visuospatial processing (three tasks—2D Manipulations, Four Towers/3D Scene Rotation, Picture Completion), emotion discrimination (one task), spatial planning (two tasks—Blocks and Tower of London), cognitive control (two tasks—Switching Stroop, Trail Making), semantic reasoning (one task—Verbal Analogies) and crystalised intelligence (one task—Word Definitions). Both batteries included the Motor Control task to measure visuomotor processing times at the time of assessment, which was expected to be affected by the primary motor deficits of PD. Details of task designs can be found in the Supplementary Materials–Supplementary Task Descriptions and Supplementary Table 1.

**Table 2:** Batteries administered to participants

Battery 1 (Day 1)	Operational complexity	Battery 2 (Day 2)	Operational complexity
Motor control	1	Motor control	1
Recognition memory immediate	1	SRT	1
Target detection	1	Trail making	1
Emotional discrimination	1	Paired associate learning	2
2D manipulations	1	Switching Stroop	3
Digit Span	2	Picture completion	3
Spatial Span	2	Card Pairs	3
Blocks	3	3D Scene rotation (four towers)	3
Tower of London	3		
Verbal Analogies	3		
Word definitions	3		
Recognition memory delayed	2		

Operational complexity: 1. Individual responses to trials based on simple rules. 2. Multiple responses to trials based on information in working memory. 3. Responses require more complex reasoning/problem-solving.

### Statistical analysis

Preprocessing was performed in Python with statistical analysis using^50^.

Statistical analysis focused on the summary scores for each task, these being measures of response accuracy or response latency (Supplementary Table S1). For inferring group differences, a linear regression model was first fitted to factor out confounding effects of sociodemographic variables (predictors: age in decade categories to allow for non-linear age effects; sex, male or female; education, none, less than 5 years, 5–10 years or more than 10 years). The residuals were rank transformed to normality. Two-way analysis of variance (ANOVA) examined whether the adjusted scores varied with the factors of clinical group and task and their two-way interaction. All scores were input to the ANOVA with high = good and low = poor performance. Tukey post-hoc tests confirmed which group differences drove any significant effects. Global performance was estimated by applying factor analysis (FA) with one unrotated factor for participants who completed all tasks. To interpret effect sizes, we conformed with Sawilowsky’s extended version of Cohen’s notion of effect sizes (0.1 SD = very small, 0.2 SD = small, 0.5 SD = medium, 0.8 SD = large, 1.2 SD = very large and 2.0 SD = huge)^51^ .

To compare group effect sizes relative to normal age-related decline, the same summary scores were examined within the large previously collected online cohort. Specifically, performances of people in their 80 s, 70 s and 60 s were contrasted with those in their 50 s using a linear regression model with age as a categorical factor to allow for non-linear associations.

Exploratory factor analysis conformed to the Kaiser convention of defining the number of factors as the number of eigenvalues greater than 1^52^. Varimax rotation reoriented the resultant factors to produce parsimonious loadings.

To select the tasks for the final battery, we considered (a) which tasks had scores that were discriminative when contrasting PD and RBD to controls, (b) which tasks had scores that were sensitive to the device the assessment was run on and (c) the dispersion of the tasks in terms of their loadings on different factors from the FA. To ensure tasks were not excluded if only sensitive to impairments in one or other clinical group, effect size ranges were defined as the greatest difference between the means of the patient groups relative to the Discovery Cohort controls. As Discovery Cohort participants primarily used personal computers for the assessment, sensitivity to the device was defined as the mean difference between scores of phone and computer users in the large preexisting normative dataset.

Both task scores and the MoCA scores were adjusted to sociodemographic variables (age, decade, sex, education) using a linear model. A linear regression was then fitted to predict the MoCA score from the performance on the subset of tasks recommended for the brief assessment battery. Pearson correlation coefficients were also calculated between the MoCA score and each individual task metric. The reduced battery composite was also predicted using linear regression from the MoCA subscales, where categorical scores of each section in the MoCA scale were used as predictors.

### Sensitivity analyses

To evaluate the robustness of the results, the following sensitivity analyses were conducted.

To assess the generalisability of the findings, the linear models contrasting global cognitive performance between the three groups were rerun using leave-one-out and 25-fold cross-validation, and the correlation between the observed and predicted scores was measured.

To assess the impact of using a two-step modelling approach with categorical age, models contrasting between groups were re-estimated (a) including sociodemographic variables in the model contrasting patient groups to account for residual confounding, (b) using one model with all variables included instead of first factoring out demographic variables and (c) with age fitted as a continuous variable taken to the third order instead of as a categorical factor.

To assess the impact on the results of modelling PD as a single group, supplementary analyses were conducted with PD sub-categorised according to probable RBD, as defined with a cutoff of >6 points on the RBD scale. Models were run (a) contrasting global cognitive composites between PD with vs. without RBD, (b) for RBD with vs. without PD and (c) for all three patient groups vs. Controls.

To explore how bradykinesia in the PD group might affect PD participants task responses, correlations were run between global cognitive composites and the final battery composite and (a) the UPDRS-III and (b) a composite calculated across the finger tapping and hand movement difficulty sub-scales.

To examine the generalisability of the associations between the online computerised battery and pen-and-paper supervised assessment, the regression and correlation analyses were rerun compared to the MMSE, which was collected at enrolment only.

### Supplementary information

Supplementary Materials - final

## Data Availability

Requests for data or for setting up the cognitive assessment battery to be used in other studies should be directed to Professor Adam Hampshire (a.hampshire@imperial.ac.uk). Data is available upon reasonable request.
