# Supplementary material for: Online cognitive monitoring technology for people with Parkinson’s disease and REM sleep behavioural disorder
Source: NPJ Digit Med. 2024 May 7;7:118. doi: 10.1038/s41746-024-01124-6 (PMC11076465; doi:10.1038/s41746-024-01124-6)
Supplement: Supplementary file 1 — Supplementary Materials - final [file 41746_2024_1124_MOESM1_ESM.pdf]

Developing and validating online cognitive monitoring technology for people with Parkinson's disease and REM sleep behavioural disorder

Maria Bălăeţ[1]*, Falah Alhajraf[2], Tanja Zerenner[3], Jessica Welch[2], Jamil Razzaque[2], Christine Lo[2], Valentina Giunchiglia[1], William Trender[1], Annalaura Lerede[1], Peter J Hellyer[4], Sanjay G Manohar[2], Paresh Malhotra[1], Michele Hu[2], Adam Hampshire[4,1]

1. Department of Brain Sciences, Imperial College London, London, United Kingdom
2. Oxford Parkinson's Disease Centre, Nuffield Department Clinical Neurosciences, University of Oxford, Oxford, United Kingdom
3. Population Health Sciences, University of Bristol, Bristol, United Kingdom
4. Centre for Neuroimaging Sciences, Institute of Psychiatry, Psychology and Neuroscience, King's College London, London, United Kingdom

*Correspondence: m.balaet17@imperial.ac.uk

Supplementary Materials

Supplementary Task Descriptions
Supplementary Tables 1-6
Supplementary Discussion – further information on statistical testing presented in Figure 1
Supplementary Sensitivity Analyses:

- **Supplementary Tables 7-23**
- **Supplementary Figures 1-14**
- **Supplementary Model Outputs**

SUPPLEMENTARY TASK DESCRIPTIONS

The tasks descriptions have been adapted from Hampshire et al., 2021.

Hampshire, A., Trender, W., Chamberlain, S.R., Jolly, A.E., Grant, J.E., Patrick, F., Mazibuko, N., Williams, S.C., Barnby, J.M., Hellyer, P., Mehta, M.A., 2021. Cognitive deficits in people who have recovered from COVID-19. EClinicalMedicine 39, 101044. https://doi.org/10.1016/j.eclinm.2021.101044

Motor control

This task is designed to measure motor control - which is defined as the ability to click on discrete stimuli on the screen quickly and with high accuracy. Participants must click on a number of targets that appear at random points on the screen. The mean reaction time, as well as the mean (euclidean) distance from the target are measured.

Recognition memory (immediate and delayed)

The test measures recognition memory for words at different time delays. The participant is presented with a sequence of objects in a pseudo-random order, each presented for 1000 ms and with an inter-image interval of 200 ms. The participant is then immediately presented with a sequence of 24 objects. 12 of the objects are the ones they were asked to commit to memory and 12 are new words, six of which are semantically similar foils to the ones committed to memory. At the end of the battery of tests, the participant’s recognition memory is probed again for the same target items alongside a different set of foils and distractors. The primary output for both immediate and delayed word memory is the sum of correct responses, with maximum score being 24.

Target detection

The Target Detection test measures spatial visual attention. The participant is presented with a target shape on the left of the screen and a probe area on the right side of the screen. After 3000ms, the probe area begins to fill with shapes, the participant must identify and click the target shape while ignoring the distractor shapes. Shapes are added every 1000ms and a subset of the shapes in the probe area are removed every 1000ms. The trial runs for a total of 120 addition/removal cycles. The target shape is included in the added shapes pseudo randomly, at a frequency of 12 in 20 cycles. The primary output is the total number of target shapes clicked.

Emotional discrimination

This test measures an individual’s ability to identify and discern between emotions. Participants are presented with pictures of two people, each expressing a particular emotion (e.g. happy, neutral, angry, scared). They must decide if the emotions expressed by each person are the same or different. Trials vary based on the emotions used as well as whether individuals have congruent vs. incongruent emotional expressions. To obtain maximum points, participants must complete 50 trials as accurately as possible. For every correct answer, the total score increases by one point. The outcome measure is the total score.

2D manipulations

The 2D Mental Manipulation test measures the ability to spatially manipulate objects in mind (Silverman et al., 2000). In this version of the test, a grid with coloured squares is presented at the top of the screen, with a further four grids with coloured squares presented below (i.e. probe grids). One of the four grids is identical to the target grid above but is rotated by either 90, 180 or 270 degrees whilst the other grids differ by five squares. To obtain maximum points, the participant must indicate which of the four grids is identical to target, solving as many problems as possible within three minutes.

Digit Span

This task is a proxy for working memory. Participants must remember a string of digits that increment in length. Each time participants remember the full string, the next string displayed increments in length by one unit. The total amount of strings participants recalled fully is recorded.

Spatial Span

The Spatial Span test measures spatial short-term memory capacity. It is a variant on the classic Corsi Block Tapping Test (Corsi, 1972). The participant is presented with a 4 x 4 grid, onto which is displayed a sequence of squares in different positions in the grid. The participant must then click the squares in the order that they were highlighted. The difficulty is incremented using a ratchet system, every time a sequence is recalled correctly, the length of the subsequent sequence is incremented by one. The test is terminated when three consecutive mistakes are made on a particular sequence length. The primary output is the maximum sequence length correctly recalled. Minimum level = 2, maximum level = 16, ISI = 0ms, encoding time = 1500ms.

Blocks

Participants must remove blocks from one array until it matches a target array; blocks will fall under gravity. The Blocks task has been adapted from a common analogue neuropsychological test in which the subject has to match a shape using coloured blocks. This is thought to be a good measure of spatial visualisation skills. The current version also incorporates a measure of planning as you have to predict the shapes that will be created once a block is removed and gravity takes effect.

Tower of London

The Tower of London test measures spatial planning. It is a variant on the original Tower of London Test (Shallice, 1982). The participant is shown two sets of three prongs with coloured beads on them. The first set is the initial state and the second set is the target state. The participant must work out the lowest number of moves it would take to transition from the initial state to the target state. They must then input this number using an on-screen number pad. This differs from the original test in that the participant is not allowed to move the beads, all calculation and planning must be done in their head. This is to prevent correct answers being reached through iterative error correction. The test consists of 10 trials of variable difficulty. The difficulty is scaled using the number of beads and the convolutedness, defined as the number of moves that must be made that do not place a bead in its final target position. The outcome measure is the total number of correct trials.

Verbal Analogies

The Analogical Reasoning test measures semantic reasoning abilities. In this version of the test, participants are presented with two written relationships that they must decide have the same type of association or not (e.g. “Lion is to feline as cabbage is to vegetable”). Participants must indicate their decision by selecting the True or False buttons presented below the written analogies. Analogies are varied across semantic distance to modulate difficulty and association types switch throughout the sequence of trials. To obtain maximum points, participants must solve as many problems as possible within three minutes. For every correct response, the total score increases by one. For every incorrect response, the total score decreases by one. The outcome measure is the total score.

Word Definitions

In this test, individuals are assessed on their ability to identify the correct definitions of words. Participants are presented with a word accompanied by four descriptive statements. They must decide which of the four statements provides the correct definition of the word. Words vary based on their frequency of use in English written language, resulting in rare and commonly used words being presented. For each word, the participant has twenty seconds to choose a definition. To obtain maximum points, participants must answer 21 word-definitions correctly. For every correct response, the total score increases by one point. The outcome measure is the total score.

Simple reaction task

This task is the simplest task available for measuring reaction time. Participants must respond as quickly as they can to a stimulus that appears on the screen at different timepoints. What is measured is the total time it takes for the participant to press on the screen after the stimulus appeared.

Trail making

Trail Making is based on a classical pen and paper neuropsychological test. The first section, containing only numbers reflects the speed at which the participant is able to process a field of information and sort it accurately. The second section, in which there are both numbers and letters requires ‘attentional switching’, which is an important executive function. Subtracting the former from the latter provides a

measure of the ‘switching cost’. What is measured is the time required by the participant to click on all the targets in the correct order.

Pairs associate learning

This task is a measure of visual working memory. Participants must remember a series of objects and their associated locations on a grid. What is measured is the total amount of correct trials.

Switching stroop

This task is a proxy for executive function, and is a routine cognitive test that is used to classify patients suffering with conditions such as traumatic brain injury. Participants must describe the colour of a central tile by choosing between two words reading "Red" and "Blue" and coloured either red or blue. The participant must pay attention to either the text of the word or the colour of the word at any given time. What is recorded is the total number of correct trials.

Picture completion

This task is assessing the ability of the participants to identify how patterns taken out of their original context fit back within the context. This taps into visual processing and pattern recognition abilities. Participants must correctly assign missing pieces from a picture. The pieces can be rotated through 90, 180 or 270 degrees. What is recorded is the total number of errors participants make while trying to piece the pictures back together.

Card pairs

This task is a measure of working memory. Participants are shown an array of face up cards. These cards are then placed face down and the participant must remember and identify the locations of pairs of cards. What is measured is the percentage of pairs of cards the participant correctly identified. Formerly, card pairs games used to be a popular computerised entertainment game back in the 90'.

Four towers (3D Scene rotation)

Four Towers/Faulty Towers is a 3D Perspective Rotation test that measures the ability to picture a 3D scene and rotate it in the mind’s eye. The participant is presented with an array comprising four perspective images each containing three tower blocks. Three of the images show the same tower blocks arranged in the same way but viewed from different perspectives. One of the images is the odd one out as it differs either by the type of tower blocks that are present or the relative locations that they are placed in. The participant must identify the odd one out as quickly and accurately as they can. A total of 12 problems are presented and the score increments or decrements by 1 dependent on whether or not the participant correctly identifies the odd one out. The primary output is total score.

Task	Primary Measure	Definition of Primary Measure	Secondary Measure	Definition of Secondary Measure
Motor Control	Accuracy	Mean Euclidean distance from the target, assessing precision in motor responses.	Mean Reaction Time	Average time taken to respond to motor control stimuli, assessing speed and accuracy.
Immediate Recognition Memory	Total Correct Responses	Sum of correctly identified words immediately after presentation, indicating immediate memory recall.	Median Reaction Time	Median time to react to each stimulus in the immediate memory test.
Delayed Recognition Memory	Total Correct Responses	Sum of correctly identified words after a delay, reflecting delayed memory retention.	Median Reaction Time	Median time to react to each stimulus in the delayed memory test.
2D Manipulations	Total Correct Responses	Number of spatial problems correctly solved, indicating spatial manipulation ability.	Median Reaction Time	Median time to react to each spatial manipulation problem.
Emotion Discrimination	Total Correct Responses	Number of correctly identified emotions in images, measuring emotion recognition accuracy.	Median Reaction Time	Median time to react to each emotion discrimination trial.
Blocks	Total Correct Responses	Number of correctly matched block arrangements, assessing spatial visualization and planning skills.	Median Reaction Time	Median time to react to each block arrangement task.

Target Detection	Total Correct Responses	Total number of correctly identified target shapes, reflecting spatial attention and accuracy.	Mean Reaction Time	Average time to react to each target detection stimulus.
Spatial Span	Total Correct Responses	Longest sequence length correctly recalled in a spatial memory task.	Median Reaction Time	Median time to react during each spatial memory recall attempt.
Verbal Analogies	Total Correct Responses	Total number of correctly solved semantic analogies, indicating semantic reasoning ability.	Median Reaction Time	Median time to react to each analogy problem.
Word Definitions	Total Correct Responses	Number of correctly identified word definitions, measuring vocabulary knowledge and comprehension.	Median Reaction Time	Median time to react to each word definition choice.
Simple Reaction Time	Median Reaction Time	Median time taken to respond to a basic visual stimulus, measuring basic reaction speed.	-	-
Trail Making	Mean Reaction Time	Mean reaction time between letter and number reaction times in trail making tasks.	Mean Number Reaction Time	Average time to respond to number sequence tasks.
			Mean Letter Reaction Time	Average time to respond to letter sequence tasks.
			Number/Letter Cost	Additional time (cost) of switching between numbers and letters.

PAL (Paired Associates Learning)	Total Correct Responses	Number of correctly recalled object-location pairs, indicating visual working memory capacity.	Median Reaction Time	Median time to react during each object-location recall attempt.
Switching Stroop	Total Correct Responses	Total number of correctly completed trials in a Stroop task, assessing cognitive flexibility and inhibition.	Mean Incongruent Accuracy	Accuracy in identifying incongruent color-word matches.
			Median Reaction Time	Median time to react to each Stroop task stimulus.
			Mean Switch Accuracy	Accuracy in correctly identifying switches between color and text.
			Median Switch Reaction Time	Median time to react during switch tasks in the Stroop task.
			Median Incongruent Reaction Time	Median reaction time for identifying incongruent color-word matches.
Picture Completion	Total Errors	Number of errors made in picture completion tasks, assessing visual processing and pattern recognition.	Total Time Taken	Total duration taken to complete all picture completion tasks.
Digit Span	Total Correct Responses	Number of correctly remembered digit sequences, indicating working memory capacity.	Median Reaction Time	Median time to react during each digit recall attempt.

Tower of London	Total Correct Responses	Total number of correctly planned moves in a spatial planning task, assessing executive function and planning skills.	Median Reaction Time	Median time to react to each planning challenge in the task.
Four Towers	Total Correct Responses	Number of correctly identified unique towers in a 3D perspective rotation task, measuring spatial visualization ability.	Median Reaction Time	Median time to react to identify the unique tower.
Card Pairs	Percentage of Cards Correctly Identified	Percentage of card pairs correctly identified, assessing memory and attention to detail.	Median Array Time	Median time taken to identify pairs of cards in the memory task.

Supplementary Table 1. Descriptions of tasks primary and secondary measures.

	CON	PD	RBD	Normative Data
Total Unique Participants	51	59	54	302707
Recent Clinical Assessment Available	51	59	54	N/A
Completed Every Single Task	47	56	50	N/A
Recognition Memory - Immediate	50	59	54	63927
Target Detection	49	59	54	338504
Emotion Discrimination	49	59	54	285267
Manipulations 2D	49	59	54	337937
Digit Span	48	59	54	339589
Spatial Span	48	59	54	339506
Word Definitions	48	59	54	338659
Blocks	48	59	54	343238
Verbal Analogies	48	59	54	339605
Tower of London	48	59	53	341962
Recognition Memory - Delayed	49	58	53	62649
Paired Associates Learning	48	58	52	3280
Switching Stroop	48	58	52	6553
Card Pairs	48	58	51	6510
Picture Completion	48	57	50	2279
Four Towers	47	56	50	77365
Motor Control	50	59	54	32572
Trail Making	48	58	52	7362
Simple Reaction Time	47	58	53	4283

Supplementary Table 2. Number of patients and controls completing each individual task.

Task Name	Device	N
Word Definitions	Computer	184004
	Phone	123229
Four Towers	Computer	53006
	Phone	20612
Paired Associates Learning (PAL)	Computer	2555
	Phone	543
Simple Reaction Time (SRT)	Computer	3084
	Phone	1012
Tower of London (TOL)	Computer	185982
	Phone	124342
Blocks	Computer	186669
	Phone	124806
Card Pairs	Computer	4829
	Phone	1318
Digit Span	Computer	184900
	Phone	123324
Emotion Discrimination	Computer	144200
	Phone	112456
Manipulations 2D	Computer	183625
	Phone	122943
Motor Control	Computer	20488
	Phone	11079
Picture Completion	Computer	1752
	Phone	361
Recognition Memory - Delayed	Computer	41775
	Phone	17923
Recognition Memory - Immediate	Computer	42532
	Phone	18356
Spatial Span	Computer	184427
	Phone	123612
Switching Stroop	Computer	4899
	Phone	1333
Target Detection	Computer	183975
	Phone	123125

Supplementary Table 3. Number of participants in the normative data using different devices.

group1	group2	meandiff	p	lower	upper	sig	variable	sig_corrected
CON	PD	-0.6763	0.0008	-1.1063	-0.2463	TRUE	Total Words Remembered - Immediate Recognition Memory	TRUE
CON	RBD	-0.4473	0.0447	-0.8863	-0.0083	TRUE	Total Words Remembered - Immediate Recognition Memory	FALSE
PD	RBD	0.229	0.4011	-0.19	0.6481	FALSE	Total Words Remembered - Immediate Recognition Memory	FALSE
CON	PD	-0.2273	0.4523	-0.6737	0.2192	FALSE	Total Words Remembered - Delayed Recognition Memory	FALSE
CON	RBD	-0.3564	0.1592	-0.814	0.1013	FALSE	Total Words Remembered - Delayed Recognition Memory	FALSE
PD	RBD	-0.1291	0.7622	-0.5638	0.3056	FALSE	Total Words Remembered - Delayed Recognition Memory	FALSE
CON	PD	-0.3134	0.2217	-0.7586	0.1318	FALSE	Total Correct Responses - 2D Manipulations Task	FALSE
CON	RBD	-0.1952	0.5678	-0.6496	0.2593	FALSE	Total Correct Responses - 2D Manipulations Task	FALSE
PD	RBD	0.1182	0.7955	-0.3156	0.552	FALSE	Total Correct Responses - 2D Manipulations Task	FALSE
CON	PD	-0.2311	0.4368	-0.6754	0.2131	FALSE	Total Correct Responses - Emotion Discrimination Task	FALSE
CON	RBD	-0.3375	0.1862	-0.791	0.116	FALSE	Total Correct Responses - Emotion Discrimination Task	FALSE
PD	RBD	-0.1064	0.8302	-0.5393	0.3265	FALSE	Total Correct Responses - Emotion Discrimination Task	FALSE
CON	PD	-0.3804	0.1098	-0.825	0.0643	FALSE	Total Correct Responses - Blocks Task	FALSE
CON	RBD	-0.3602	0.1484	-0.814	0.0936	FALSE	Total Correct Responses - Blocks Task	FALSE
PD	RBD	0.0202	0.9933	-0.4106	0.451	FALSE	Total Correct Responses - Blocks Task	FALSE
CON	PD	-0.7284	0.0002	-1.1557	-0.3012	TRUE	Total Correct Responses - Target Detection Task	TRUE
CON	RBD	-0.2967	0.2445	-0.7328	0.1394	FALSE	Total Correct Responses - Target Detection Task	FALSE
PD	RBD	0.4317	0.0401	0.0154	0.848	TRUE	Total Correct Responses - Target Detection Task	FALSE
CON	PD	-0.529	0.0144	-0.971	-0.087	TRUE	Total Correct Responses - Switching Stroop Task	TRUE
CON	RBD	-0.3234	0.2129	-0.7768	0.1299	FALSE	Total Correct Responses - Switching Stroop Task	FALSE
PD	RBD	0.2056	0.5004	-0.227	0.6382	FALSE	Total Correct Responses - Switching Stroop Task	FALSE
CON	PD	0.4179	0.0716	-0.0283	0.8641	FALSE	Total Correct Responses - Trail Making Task	FALSE
CON	RBD	0.2904	0.2931	-0.1673	0.7481	FALSE	Total Correct Responses - Trail Making Task	FALSE
PD	RBD	-0.1275	0.7691	-0.5642	0.3092	FALSE	Total Correct Responses - Trail Making Task	FALSE

CON	PD	-0.3401	0.1724	-0.7869	0.1066	FALSE	Total Correct Responses - Spatial Span Task	FALSE
CON	RBD	-0.1758	0.6334	-0.6317	0.2801	FALSE	Total Correct Responses - Spatial Span Task	FALSE
PD	RBD	0.1644	0.6421	-0.2685	0.5972	FALSE	Total Correct Responses - Spatial Span Task	FALSE
CON	PD	-0.3501	0.1478	-0.7906	0.0904	FALSE	Total Correct Responses - Verbal Analogies Task	FALSE
CON	RBD	-0.534	0.0153	-0.9836	-0.0845	TRUE	Total Correct Responses - Verbal Analogies Task	TRUE
PD	RBD	-0.184	0.5655	-0.6108	0.2428	FALSE	Total Correct Responses - Verbal Analogies Task	FALSE
CON	PD	-0.102	0.8537	-0.5524	0.3483	FALSE	Total Correct Responses - Digit Span Task	FALSE
CON	RBD	-0.1609	0.6861	-0.6205	0.2987	FALSE	Total Correct Responses - Digit Span Task	FALSE
PD	RBD	-0.0589	0.9454	-0.4952	0.3775	FALSE	Total Correct Responses - Digit Span Task	FALSE
CON	PD	-0.4134	0.069	-0.8518	0.0249	FALSE	Total Words Remembered - Word Definitions Task	FALSE
CON	RBD	-0.5715	0.0082	-1.0189	-0.1242	TRUE	Total Words Remembered - Word Definitions Task	TRUE
PD	RBD	-0.1581	0.6532	-0.5828	0.2666	FALSE	Total Words Remembered - Word Definitions Task	FALSE
CON	PD	-0.2385	0.4228	-0.6882	0.2112	FALSE	Total Correct Responses - Paired Associates Learning Task	FALSE
CON	RBD	0.0115	0.9981	-0.4498	0.4728	FALSE	Total Correct Responses - Paired Associates Learning Task	FALSE
PD	RBD	0.25	0.3729	-0.1901	0.6902	FALSE	Total Correct Responses - Paired Associates Learning Task	FALSE
CON	PD	-0.12	0.8049	-0.5721	0.3321	FALSE	Percentage Correct Cards - Card Pairs Task	FALSE
CON	RBD	-0.18	0.632	-0.6459	0.2859	FALSE	Percentage Correct Cards - Card Pairs Task	FALSE
PD	RBD	-0.06	0.9453	-0.5048	0.3847	FALSE	Percentage Correct Cards - Card Pairs Task	FALSE
CON	PD	-0.2462	0.4105	-0.7028	0.2103	FALSE	Total Achieved - Four Towers Task	FALSE
CON	RBD	-0.0498	0.9658	-0.5187	0.4191	FALSE	Total Achieved - Four Towers Task	FALSE
PD	RBD	0.1964	0.5556	-0.2526	0.6455	FALSE	Total Achieved - Four Towers Task	FALSE
CON	PD	0.0012	1.0	-0.449	0.4513	FALSE	Total Achieved - Tower of London Task	FALSE
CON	RBD	0.1528	0.7137	-0.3086	0.6143	FALSE	Total Achieved - Tower of London Task	FALSE
PD	RBD	0.1517	0.692	-0.2866	0.59	FALSE	Total Achieved - Tower of London Task	FALSE
CON	PD	0.236	0.408	-0.1999	0.6719	FALSE	Mean Reaction Time - Motor Control Task	FALSE
CON	RBD	-0.2761	0.3092	-0.7211	0.1689	FALSE	Mean Reaction Time - Motor Control Task	FALSE

PD	RBD	-0.5121	0.0142	-0.9391	-0.0851	TRUE	Mean Reaction Time - Motor Control Task	TRUE
CON	PD	0.4076	0.0825	-0.0401	0.8553	FALSE	Median Reaction Time - Simple Reaction Time Task	FALSE
CON	RBD	0.4029	0.096	-0.0541	0.86	FALSE	Median Reaction Time - Simple Reaction Time Task	FALSE
PD	RBD	-0.0047	0.9996	-0.4381	0.4288	FALSE	Median Reaction Time - Simple Reaction Time Task	FALSE
CON	PD	0.4444	0.0517	-0.0026	0.8914	FALSE	Total Errors - Picture Completion Task	FALSE
CON	RBD	0.2736	0.341	-0.1874	0.7347	FALSE	Total Errors - Picture Completion Task	FALSE
PD	RBD	-0.1707	0.6323	-0.6129	0.2714	FALSE	Total Errors - Picture Completion Task	FALSE

Supplementary Table 4. Tukey post-hoc tests for primary measures

group1	group2	meandiff	p	lower	upper	sig	variable	sig_corrected
CON	PD	0.3092	0.2301	-0.1356	0.754	FALSE	Immediate Prospective Memory - Median Reaction Time	FALSE
CON	RBD	0.0639	0.9407	-0.3901	0.518	FALSE	Immediate Prospective Memory - Median Reaction Time	FALSE
PD	RBD	-0.2453	0.3758	-0.6787	0.1881	FALSE	Immediate Prospective Memory - Median Reaction Time	FALSE
CON	PD	0.5539	0.009	0.116	0.9918	TRUE	Delayed Prospective Memory - Median Reaction Time	TRUE
CON	RBD	0.1388	0.7451	-0.3101	0.5877	FALSE	Delayed Prospective Memory - Median Reaction Time	FALSE
PD	RBD	-0.4151	0.0582	-0.8415	0.0112	FALSE	Delayed Prospective Memory - Median Reaction Time	FALSE
CON	PD	0.2672	0.3349	-0.1791	0.7135	FALSE	Manipulations 2D - Median Reaction Time	FALSE
CON	RBD	0.1597	0.6853	-0.2959	0.6153	FALSE	Manipulations 2D - Median Reaction Time	FALSE
PD	RBD	-0.1075	0.8286	-0.5424	0.3274	FALSE	Manipulations 2D - Median Reaction Time	FALSE
CON	PD	0.4085	0.0769	-0.0339	0.8509	FALSE	Emotion Discrimination - Median Reaction Time	FALSE
CON	RBD	0.2785	0.3135	-0.1731	0.7301	FALSE	Emotion Discrimination - Median Reaction Time	FALSE
PD	RBD	-0.13	0.7559	-0.5611	0.3011	FALSE	Emotion Discrimination - Median Reaction Time	FALSE
CON	PD	0.4807	0.0297	0.0382	0.9232	TRUE	Blocks - Median Reaction Time	FALSE
CON	RBD	0.2353	0.4355	-0.2162	0.6869	FALSE	Blocks - Median Reaction Time	FALSE
PD	RBD	-0.2453	0.3676	-0.6741	0.1834	FALSE	Blocks - Median Reaction Time	FALSE
CON	PD	0.6011	0.0037	0.1672	1.035	TRUE	Target Detection - Mean Reaction Time	TRUE
CON	RBD	0.2038	0.5225	-0.2391	0.6467	FALSE	Target Detection - Mean Reaction Time	FALSE
PD	RBD	-0.3972	0.0704	-0.82	0.0255	FALSE	Target Detection - Mean Reaction Time	FALSE
CON	PD	0.282	0.3022	-0.1682	0.7321	FALSE	Trail Making - Mean Number Reaction Time	FALSE

CON	RBD	0.1198	0.8128	-0.342	0.5816	FALSE	Trail Making - Mean Number Reaction Time	FALSE
PD	RBD	-0.1622	0.6593	-0.6028	0.2784	FALSE	Trail Making - Mean Number Reaction Time	FALSE
CON	PD	0.4638	0.0383	0.0199	0.9078	TRUE	Trail Making - Mean Letter Reaction Time	FALSE
CON	RBD	0.3771	0.1258	-0.0783	0.8325	FALSE	Trail Making - Mean Letter Reaction Time	FALSE
PD	RBD	-0.0867	0.8843	-0.5212	0.3477	FALSE	Trail Making - Mean Letter Reaction Time	FALSE
CON	PD	0.445	0.0491	0.0014	0.8885	TRUE	Trail Making - Number/Letter Cost	FALSE
CON	RBD	0.4322	0.0664	-0.0227	0.8872	FALSE	Trail Making - Number/Letter Cost	FALSE
PD	RBD	-0.0127	0.9974	-0.4468	0.4214	FALSE	Trail Making - Number/Letter Cost	FALSE
CON	PD	0.416	0.0734	-0.0304	0.8625	FALSE	Switching Stroop - Median Reaction Time	FALSE
CON	RBD	0.2132	0.5144	-0.2447	0.6712	FALSE	Switching Stroop - Median Reaction Time	FALSE
PD	RBD	-0.2028	0.5165	-0.6398	0.2341	FALSE	Switching Stroop - Median Reaction Time	FALSE
CON	PD	-0.5377	0.0125	-0.9794	-0.096	TRUE	Switching Stroop - Mean Incongruent Accuracy	TRUE
CON	RBD	-0.2833	0.3036	-0.7364	0.1698	FALSE	Switching Stroop - Mean Incongruent Accuracy	FALSE
PD	RBD	0.2544	0.3473	-0.1779	0.6867	FALSE	Switching Stroop - Mean Incongruent Accuracy	FALSE
CON	PD	0.492	0.0257	0.0485	0.9355	TRUE	Switching Stroop - Median Incongruent Reaction Time	FALSE
CON	RBD	0.3267	0.2086	-0.1282	0.7817	FALSE	Switching Stroop - Median Incongruent Reaction Time	FALSE
PD	RBD	-0.1652	0.6405	-0.5993	0.2688	FALSE	Switching Stroop - Median Incongruent Reaction Time	FALSE
CON	PD	-0.2272	0.4586	-0.6775	0.2231	FALSE	Switching Stroop - Mean Switch Accuracy	FALSE
CON	RBD	-0.2353	0.4516	-0.6973	0.2266	FALSE	Switching Stroop - Mean Switch Accuracy	FALSE
PD	RBD	-0.0082	0.9989	-0.4489	0.4326	FALSE	Switching Stroop - Mean Switch Accuracy	FALSE
CON	PD	0.3284	0.1971	-0.1207	0.7775	FALSE	Switching Stroop - Median Switch Reaction Time	FALSE
CON	RBD	0.1717	0.6523	-0.2889	0.6324	FALSE	Switching Stroop - Median Switch Reaction Time	FALSE
PD	RBD	-0.1567	0.6766	-0.5962	0.2829	FALSE	Switching Stroop - Median Switch Reaction Time	FALSE
CON	PD	0.2651	0.338	-0.1796	0.7099	FALSE	Spatial Span - Median Reaction Time	FALSE
CON	RBD	-0.1321	0.7706	-0.586	0.3218	FALSE	Spatial Span - Median Reaction Time	FALSE
PD	RBD	-0.3972	0.0776	-0.8281	0.0337	FALSE	Spatial Span - Median Reaction Time	FALSE
CON	PD	0.4885	0.0259	0.0476	0.9294	TRUE	Verbal Analogies - Median Reaction Time	FALSE
CON	RBD	0.427	0.0669	-0.023	0.8769	FALSE	Verbal Analogies - Median Reaction Time	FALSE

PD	RBD	-0.0615	0.938	-0.4887	0.3656	FALSE	Verbal Analogies - Median Reaction Time	FALSE
CON	PD	0.3674	0.1211	-0.0722	0.807	FALSE	Word Definitions - Median Reaction Time	FALSE
CON	RBD	0.556	0.0107	0.1074	1.0046	TRUE	Word Definitions - Median Reaction Time	TRUE
PD	RBD	0.1886	0.548	-0.2373	0.6145	FALSE	Word Definitions - Median Reaction Time	FALSE
CON	PD	0.4565	0.0428	0.0118	0.9013	TRUE	PAL - Median Reaction Time	FALSE
CON	RBD	0.1773	0.6287	-0.2789	0.6335	FALSE	PAL - Median Reaction Time	FALSE
PD	RBD	-0.2792	0.2854	-0.7145	0.1561	FALSE	PAL - Median Reaction Time	FALSE
CON	PD	0.29	0.2817	-0.1598	0.7397	FALSE	Card Pairs - Median Array Time	FALSE
CON	RBD	0.2288	0.4739	-0.2347	0.6923	FALSE	Card Pairs - Median Array Time	FALSE
PD	RBD	-0.0611	0.9428	-0.5036	0.3813	FALSE	Card Pairs - Median Array Time	FALSE
CON	PD	0.3721	0.1205	-0.0726	0.8168	FALSE	Picture Completion - Total Time Taken	FALSE
CON	RBD	-0.0993	0.8655	-0.5579	0.3594	FALSE	Picture Completion - Total Time Taken	FALSE
PD	RBD	-0.4714	0.0325	-0.9112	-0.0316	TRUE	Picture Completion - Total Time Taken	FALSE
CON	PD	0.2157	0.506	-0.242	0.6734	FALSE	Four Towers - Median Reaction Time	FALSE
CON	RBD	0.1338	0.7789	-0.3362	0.6039	FALSE	Four Towers - Median Reaction Time	FALSE
PD	RBD	-0.0818	0.903	-0.532	0.3683	FALSE	Four Towers - Median Reaction Time	FALSE
CON	PD	-0.1243	0.7914	-0.5754	0.3267	FALSE	Tower of London - Median Reaction Time	FALSE
CON	RBD	-0.0513	0.9628	-0.5137	0.4111	FALSE	Tower of London - Median Reaction Time	FALSE
PD	RBD	0.0731	0.9182	-0.3661	0.5122	FALSE	Tower of London - Median Reaction Time	FALSE
CON	PD	0.236	0.408	-0.1999	0.6719	FALSE	Motor Control - Mean Reaction Time	FALSE
CON	RBD	-0.2761	0.3092	-0.7211	0.1689	FALSE	Motor Control - Mean Reaction Time	FALSE
PD	RBD	-0.5121	0.0142	-0.9391	-0.0851	TRUE	Motor Control - Mean Reaction Time	TRUE
CON	PD	0.4452	0.0506	-0.0009	0.8912	FALSE	Simple Reaction Time - Median Reaction Time	FALSE
CON	RBD	0.44	0.0607	-0.0154	0.8954	FALSE	Simple Reaction Time - Median Reaction Time	FALSE
PD	RBD	-0.0052	0.9996	-0.4371	0.4267	FALSE	Simple Reaction Time - Median Reaction Time	FALSE

Supplementary Table 5. Tukey post-hoc tests for secondary measures

Supplementary Discussion - Complete Explanation for Figure 1 in the manuscript:

We assessed deficits specific to PD and RBD via task primary output measures (typically accuracy) and secondary measures (e.g., reaction times and cognitive control contrast). Two linear models were run with all participants who had complete data (N=146 participants), one for the primary and the other for the secondary measures, where the within subject factor was task and the between subject factor was patient group (PD/RBD/Control).

For primary measures, the ANOVA showed a significant main effect of group ($F(2,2976)=10.4, p<0.001$) but not task ($F(18,2976)=0, p=1$) and a significant interaction ($F(36,2976)=12.29, p<0.001$). When running a similar linear model without the controls group,

ANOVA showed no significant effect of patient group (F(1,2190)=2.69,p=0.1), task (F(18,2190)=035,p=0.99), nor an interaction (F(18,2190)=1.5, p=0.05). Therefore, to determine the basis of these differences, linear models were run independently on each task with two binary predictors, one for each clinical group (Figure 4A, 4C, 4E). We observed small to large scaled deficits, as defined by Cohen's notion of effect sizes, in primary outcome measures (mainly indicative of accuracy) for both conditions. The beta coefficients are reported in Supplementary Table S13. The ANOVA analysis of individual linear models is reported in Supplementary Table S21.

For secondary measures, the ANOVA showed a significant main effect of group (F(2,3748)=45.9,p<0.001) but not task (F(23,3748)=0,p=1) nor interaction between group and task (F(46,3748)=0.8,p=0.7). When running a similar linear model without the control group, ANOVA showed a significant effect of group (F(1,2616)=16.58,p<0.001), but not of task (F(23,2616)=0.55,p=0.95), or interaction (F(23,2616)=01.11,p=0.3). Further linear models were conducted independently for each task, with two binary predictors one for each patient group. These showed that secondary measures, predominantly of reaction times, appeared to be significantly affected in PD but not RBD. The beta coefficients are reported in Supplementary Table S14. The ANOVA analysis of individual linear models is reported in Supplementary Table S21.

The Tukey post-hoc analysis is reported in full in Supplementary Tables S4 and S5.

For comparison, sensitivities of the same task measures were analysed in relation to age decade in the large independent online cohort, contrasting people part of our normative dataset who were in their 80s, 70s and 60s at the time of completing the assessment relative to those in their 50s (Figure 1B and 1D; Supplementary Tables S22, S23). The pattern of cognitive differences was distinct to that of PD or RBD. A striking example is of the performance on Word Definitions Task, where we observe that healthy participants improve with age, as expected for a measure of crystalised intelligence, whereas both PD and RBD show significant deficits relative to controls.

Finally, both patient groups had overall poorer performance for the accuracy composite calculated via factor analysis across all tasks (for PD effect size -0.64SD, F(1,147)=10.91, p<0.001; for RBD effect size -0.44SD, F(1,147)=5,p=0.02), and took longer to react to the stimuli presented (for PD effect size 0.6SD, F(1,146)=9.42,p=0.003) relative to healthy controls part of the same cohort. The difference between RBD and PD was non-significant (mean difference 0.22SD, p=0.26) as indicated by Tukey post-hoc comparisons.

Tasks sensitive to clinical groups (final battery in bold)	Has at least one primary/secondary measure sensitive to either PD, RBD or both	Lack of device sensitivity	High loading onto relevant cognitive factors	Anticorrelated with more sensitive measures to avoid redundancy
**Word Recognition Memory Immediate**	x	x	x	x
**Word Definitions**	x	x	x	x
**Verbal Analogies**	x	x	x	x
**Switching Stroop**	x	x	x	x

**Target Detection**	x	x	x	x
**Blocks**	x	x	x	x
**Word Recognition Memory Delayed**	x	x	x	x
2D Manipulations	remove	N/A	N/A	N/A
**Trail Making**	x	x	x	x
Picture Completion	x	remove	N/A	N/A
**Emotional Discrimination**	x	x	x	x
Simple Reaction Task (SRT)	x	remove	N/A	N/A
Card Pairs	remove	N/A	N/A	N/A
Pairs Associate Learning (PAL)	x	x	remove	N/A
Spatial Span	remove	N/A	N/A	N/A
Motor Control	x	remove	N/A	N/A
Digit Span	remove	N/A	N/A	N/A
Four Towers	remove	N/A	N/A	N/A
Tower of London	remove	N/A	N/A	N/A

Supplementary Table 6. Retention of sensitive tasks explained. X indicates retention.

	**coef**	**Std err**	**t**	**P>|t|**	**[0.025**	**0.975]**
**Intercept**	0.0123	0.101	0.122	0.903	-0.188	0.213
**Recognition Memory - immediate**	0.1039	0.122	0.853	0.396	-0.139	0.347
**Target Detection**	0.1043	0.118	0.883	0.380	-0.131	0.340
**Emotion Discrimination**	0.0327	0.114	0.287	0.775	-0.195	0.260
**Word Definitions**	0.1965	0.114	1.729	0.088	-0.030	0.423
**Blocks**	-0.1253	0.129	-0.968	0.336	-0.383	0.132

**Verbal Analogies**	0.1167	0.119	0.982	0.329	-0.120	0.353
**Recognition memory - delayed**	0.1630	0.116	1.402	0.165	-0.069	0.395
**Switching Stroop**	0.2004	0.126	1.589	0.116	-0.051	0.451
**Trail Making**	-0.0959	0.115	-0.838	0.405	-0.324	0.132
**Number of observations**	87					
**Df residuals**	77					
**Df model**	9					
**R-squared**	0.248					
**P-value**	0.006					

Supplementary Table 7. Predicting the most recent MoCA score from performance on the recommended selection of tasks.

SUPPLEMENTARY SENSITIVITY ANALYSES

1. Effect of removing the sociodemographic confounds on task scores

score	R-squared – original model factoring out sociodemographics as categorical nuissance variables	R-squared – factoring out sociodemographic variablesusing age, age squared and age cube instead of categorical age decades	R-squared – original model looking at effects of patient groups	R-squared – attempting to do a second removal of sociodemographic effects whilst modelling effect size differences attributed to patient groups
Motor Control - Mean Euclidean Distance from Target	0.030	0.020	0.042	0.128
Immediate Prospective Memory - Median Reaction Time	0.136	0.150	0.019	0.166
Target Detection - Mean Reaction Time	0.132	0.157	0.067	0.195
Emotion Discrimination - Median Reaction Time	0.108	0.126	0.030	0.138
Manipulations 2D - Median Reaction Time	0.106	0.120	0.013	0.195
Digit Span - Median Reaction Time	0.172	0.178	0.035	0.183
Spatial Span - Median Reaction Time	0.087	0.108	0.030	0.121
Word Definitions - Median Reaction Time	0.035	0.031	0.053	0.100
Blocks - Median Reaction Time	0.086	0.076	0.040	0.170
Verbal Analogies - Median Reaction Time	0.154	0.153	0.047	0.235
Tower of London - Median Reaction Time	0.111	0.128	0.003	0.113
Delayed Prospective Memory - Median Reaction Time	0.130	0.154	0.060	0.209
Simple Reaction Time - Median Reaction Time	0.037	0.046	0.043	0.076
Trail Making - Mean Number Reaction Time	0.107	0.146	0.014	0.124
Trail Making - Mean Letter Reaction Time	0.096	0.113	0.041	0.208
Trail Making - Number/Letter Cost	0.074	0.078	0.043	0.153
PAL - Median Reaction Time	0.124	0.145	0.038	0.180
Switching Stroop - Median Reaction Time	0.184	0.149	0.030	0.180
Switching Stroop - Mean Switch Accuracy	0.063	0.042	0.012	0.082
Switching Stroop - Median Switch Reaction Time	0.130	0.121	0.019	0.151
Switching Stroop - Mean Incongruent Accuracy	0.140	0.103	0.051	0.187
Switching Stroop - Median Incongruent Reaction Time	0.197	0.147	0.043	0.198
Card Pairs - Median Array Time	0.115	0.141	0.016	0.151
Picture Completion - Total Time Taken	0.216	0.220	0.046	0.333
Four Towers - Median Reaction Time	0.093	0.087	0.008	0.124
Mean Reaction Time - Motor Control Task	0.094	0.107	0.048	0.137
Total Words Remembered - Immediate Recognition Memory	0.024	0.026	0.081	0.120
Total Correct Responses - Target Detection Task	0.135	0.146	0.095	0.218

Total Correct Responses - Emotion Discrimination Task	0.114	0.095	0.020	0.137
Total Correct Responses - 2D Manipulations Task	0.282	0.288	0.017	0.286
Total Correct Responses - Digit Span Task	0.136	0.109	0.004	0.118
Total Correct Responses - Spatial Span Task	0.110	0.086	0.020	0.109
Total Words Remembered - Word Definitions Task	0.094	0.103	0.058	0.169
Total Correct Responses - Blocks Task	0.046	0.053	0.030	0.089
Total Correct Responses - Verbal Analogies Task	0.148	0.130	0.049	0.201
Total Achieved - Tower of London Task	0.116	0.116	0.005	0.114
Total Words Remembered - Delayed Recognition Memory	0.052	0.058	0.022	0.080
Median Reaction Time - Simple Reaction Time Task	0.030	0.041	0.036	0.075
Total Correct Responses - Trail Making Task	0.106	0.131	0.032	0.199
Total Correct Responses - Paired Associates Learning Task	0.081	0.084	0.015	0.098
Total Correct Responses - Switching Stroop Task	0.157	0.115	0.049	0.206
Percentage Correct Cards - Card Pairs Task	0.126	0.155	0.006	0.117
Total Errors - Picture Completion Task	0.095	0.078	0.035	0.135
Total Achieved - Four Towers Task	0.070	0.084	0.012	0.096

Supplementary Table 8. R-squared for the prediction of task performance from sociodemographics when trailing different models

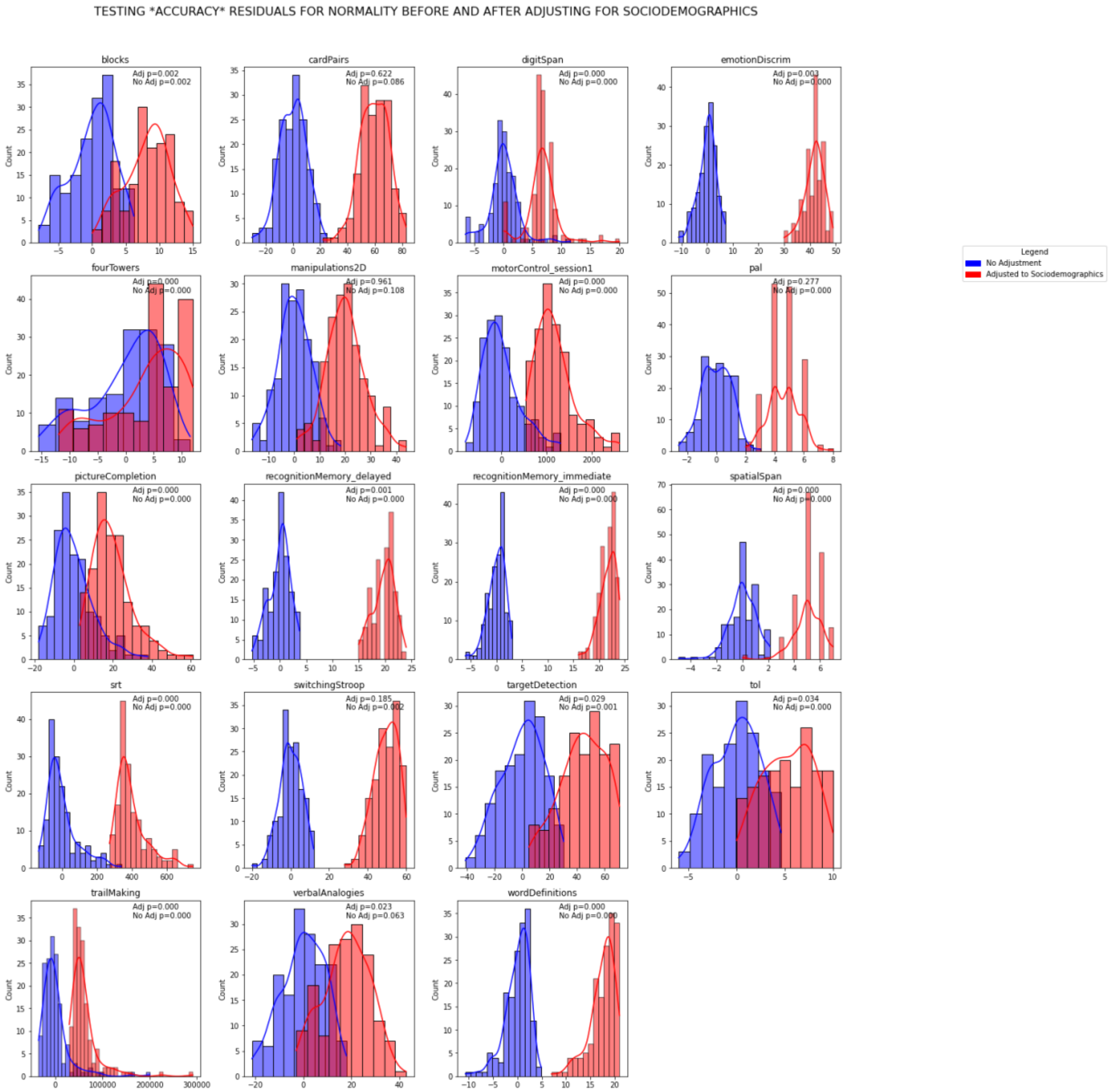

Supplementary Figure 1. Testing primary measures residuals for normality before and after removing the effect of socioedemographic confounds.

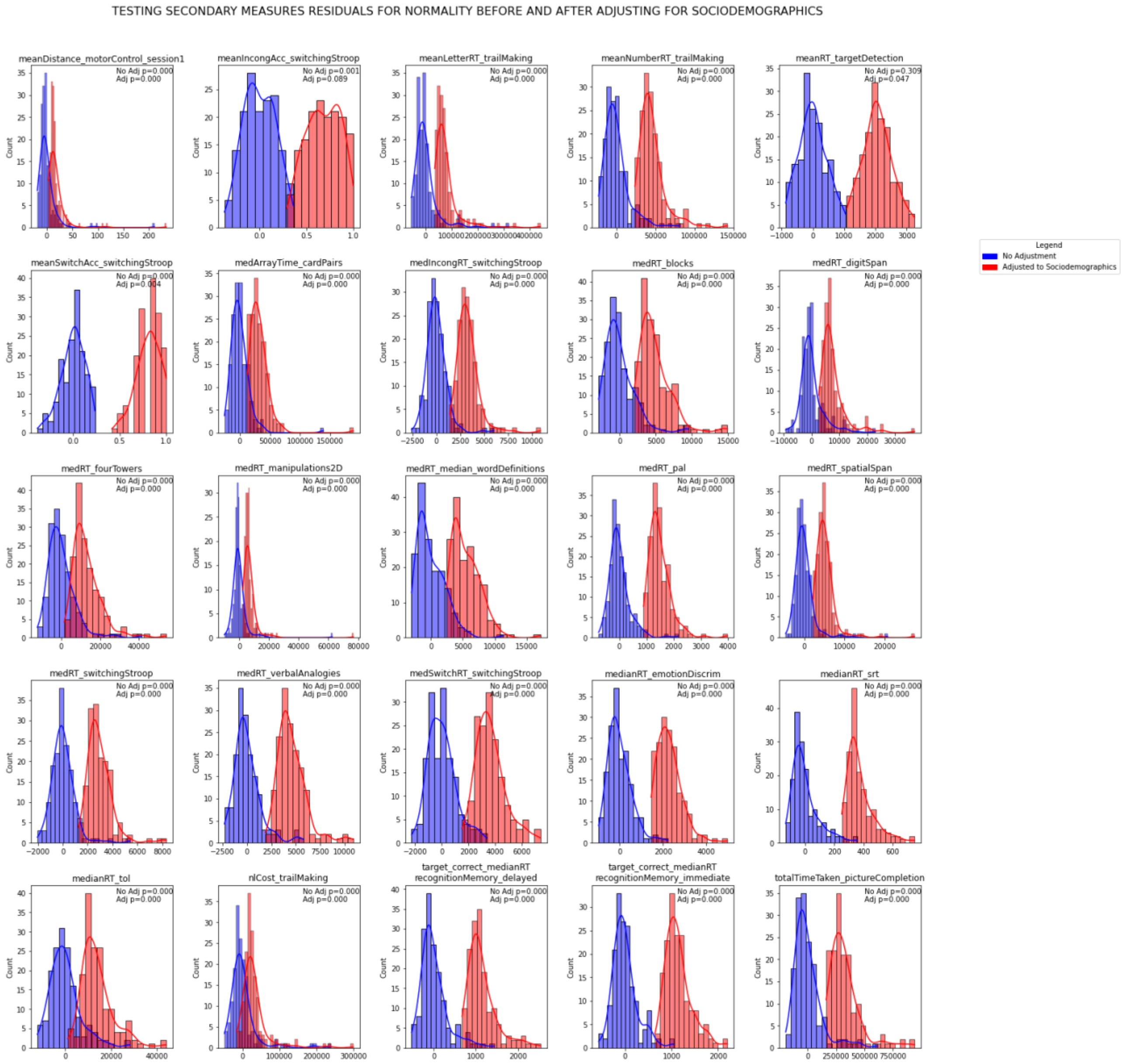

Supplementary Figure 2. Testing secondary measures residuals for normality before and after removing the effect of socioedemographic confounds.

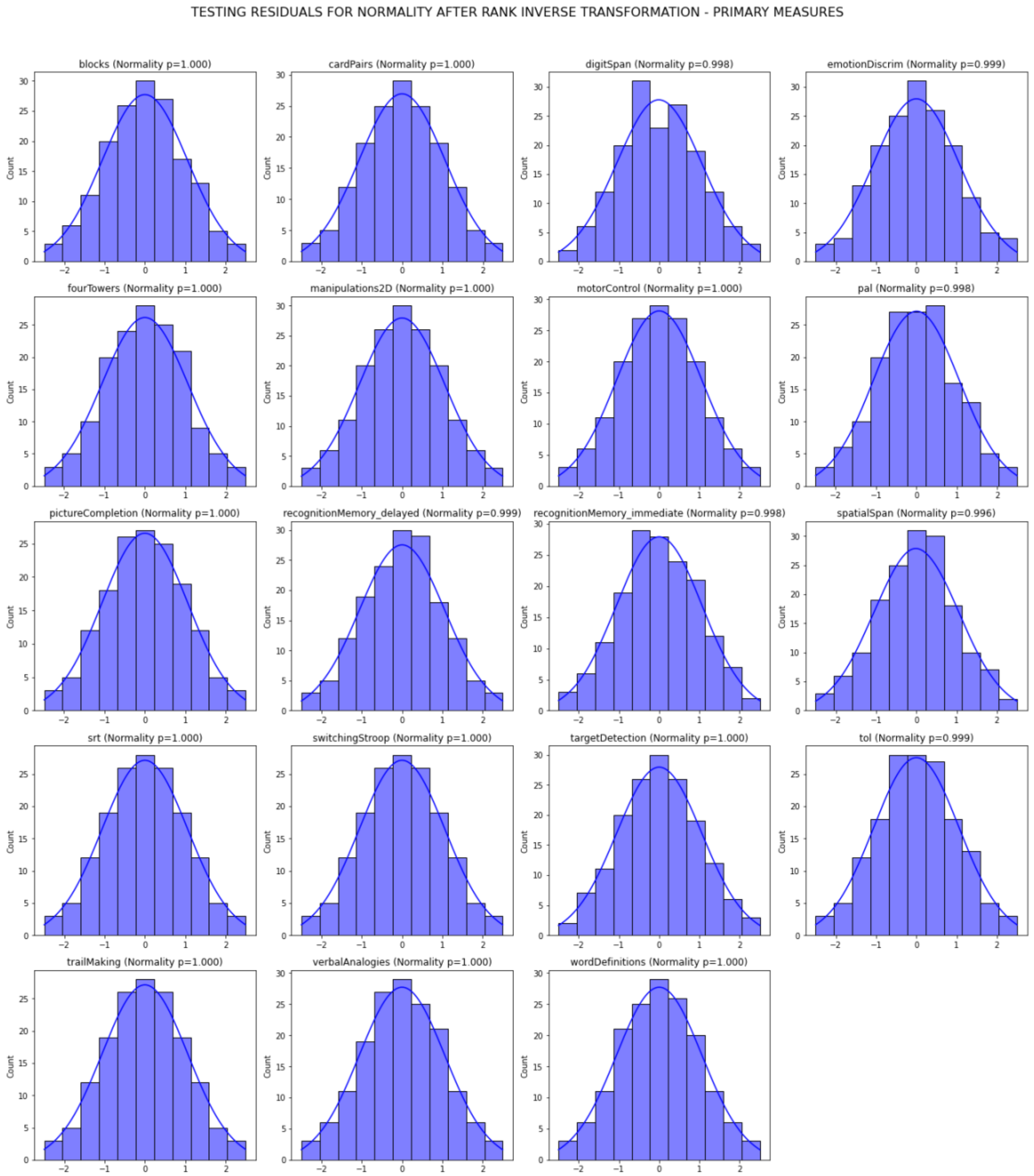

Supplementary **Figure 3. Testing rank inverse transformed residuals for normality – primary measures.**

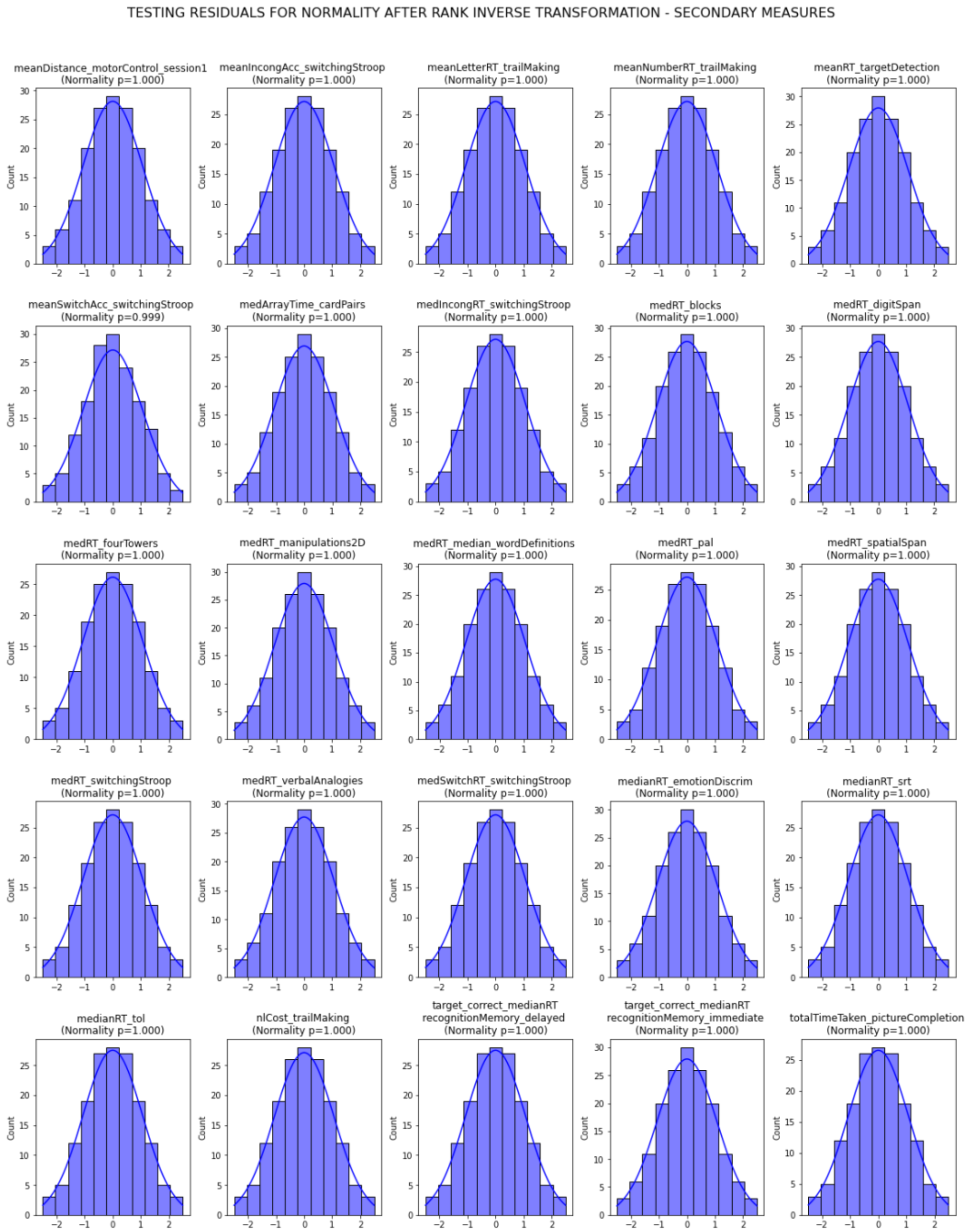

Supplementary Figure 4. Testing rank inverse transformed residuals for normality – secondary measures.

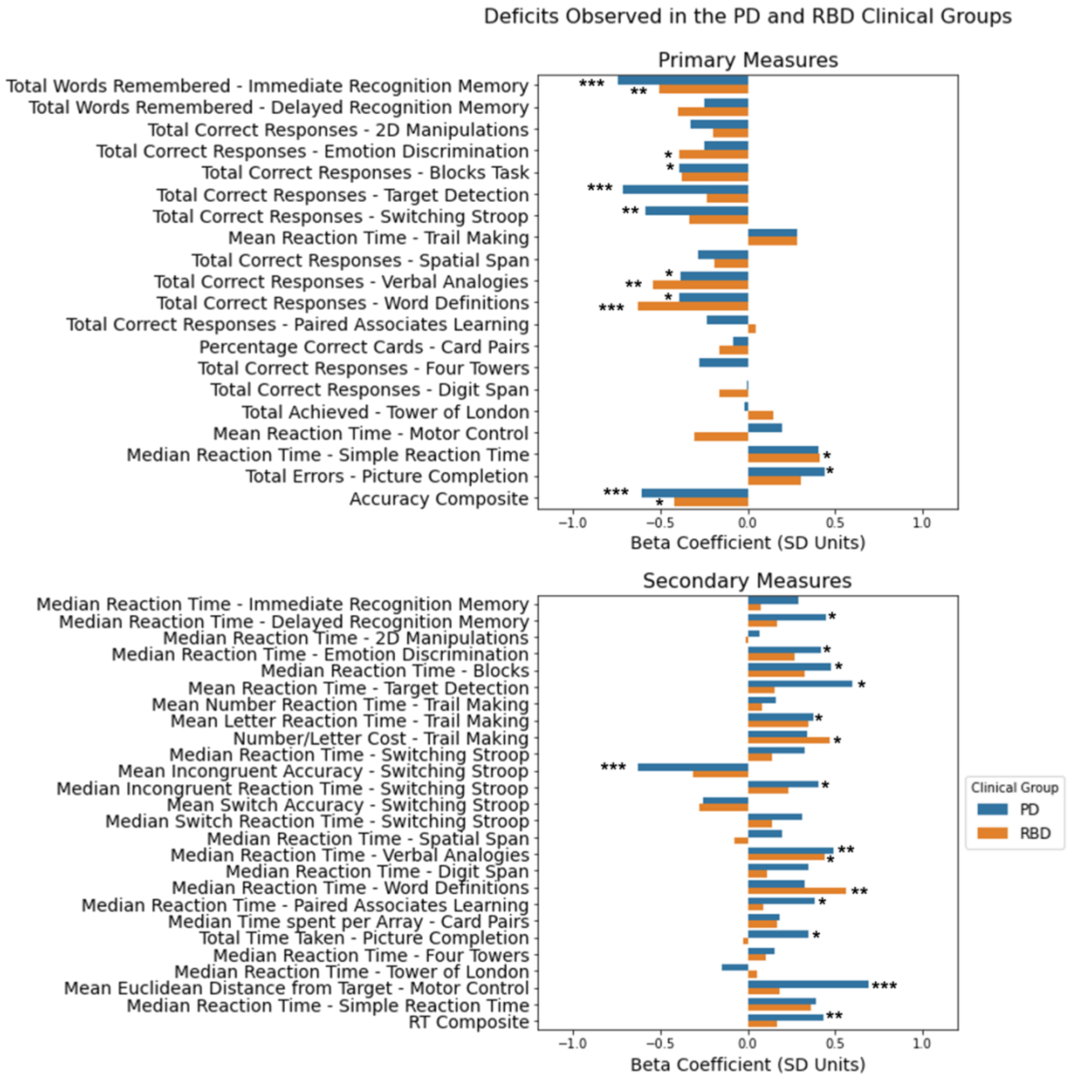

Supplementary Figure 5. Sensitivity analysis – efficacy of having removed sociodemographic confounds alongside modelling group effects using linear regression

	sum_sq	df	F	PR(>F)	task
C(age_bucket)	4.676	4.000	1.452	0.220	Total Words Remembered - Immediate Recognition Memory
C(gender)	0.004	1.000	0.005	0.944	Total Words Remembered - Immediate Recognition Memory
C(ed_bucket)	0.389	3.000	0.161	0.923	Total Words Remembered - Immediate Recognition Memory
PD	13.149	1.000	16.327	0.000	Total Words Remembered - Immediate Recognition Memory
RBD	5.784	1.000	7.183	0.008	Total Words Remembered - Immediate Recognition Memory
Residual	121.602	151.000			Total Words Remembered - Immediate Recognition Memory
C(age_bucket)	0.651	4.000	0.184	0.947	Total Words Remembered - Delayed Recognition Memory
C(gender)	3.342	1.000	3.773	0.054	Total Words Remembered - Delayed Recognition Memory
C(ed_bucket)	0.491	3.000	0.185	0.907	Total Words Remembered - Delayed Recognition Memory
PD	1.429	1.000	1.614	0.206	Total Words Remembered - Delayed Recognition Memory
RBD	3.436	1.000	3.879	0.051	Total Words Remembered - Delayed Recognition Memory
Residual	131.997	149.000			Total Words Remembered - Delayed Recognition Memory

**C(age_bucket)**	38.364	4.000	13.291	0.000	Total Correct Responses - 2D Manipulations Task
**C(gender)**	1.692	1.000	2.344	0.128	Total Correct Responses - 2D Manipulations Task
**C(ed_bucket)**	2.612	3.000	1.206	0.309	Total Correct Responses - 2D Manipulations Task
**PD**	2.543	1.000	3.523	0.062	Total Correct Responses - 2D Manipulations Task
**RBD**	0.862	1.000	1.195	0.276	Total Correct Responses - 2D Manipulations Task
**Residual**	108.963	151.000			Total Correct Responses - 2D Manipulations Task
**C(age_bucket)**	8.554	4.000	2.497	0.045	Total Correct Responses - Emotion Discrimination Task
**C(gender)**	2.161	1.000	2.524	0.114	Total Correct Responses - Emotion Discrimination Task
**C(ed_bucket)**	5.491	3.000	2.137	0.098	Total Correct Responses - Emotion Discrimination Task
**PD**	1.507	1.000	1.760	0.187	Total Correct Responses - Emotion Discrimination Task
**RBD**	3.433	1.000	4.009	0.047	Total Correct Responses - Emotion Discrimination Task
**Residual**	129.311	151.000			Total Correct Responses - Emotion Discrimination Task
**C(age_bucket)**	5.767	4.000	1.592	0.179	Total Correct Responses - Blocks Task
**C(gender)**	0.517	1.000	0.571	0.451	Total Correct Responses - Blocks Task
**C(ed_bucket)**	4.099	3.000	1.509	0.215	Total Correct Responses - Blocks Task
**PD**	3.667	1.000	4.049	0.046	Total Correct Responses - Blocks Task
**RBD**	3.126	1.000	3.452	0.065	Total Correct Responses - Blocks Task
**Residual**	135.842	150.000			Total Correct Responses - Blocks Task
**C(age_bucket)**	23.631	4.000	7.482	0.000	Total Correct Responses - Target Detection Task
**C(gender)**	2.287	1.000	2.897	0.091	Total Correct Responses - Target Detection Task
**C(ed_bucket)**	1.506	3.000	0.636	0.593	Total Correct Responses - Target Detection Task
**PD**	12.100	1.000	15.324	0.000	Total Correct Responses - Target Detection Task
**RBD**	1.228	1.000	1.555	0.214	Total Correct Responses - Target Detection Task
**Residual**	119.232	151.000			Total Correct Responses - Target Detection Task
**C(age_bucket)**	21.289	4.000	6.665	0.000	Total Correct Responses - Switching Stroop Task
**C(gender)**	0.003	1.000	0.004	0.949	Total Correct Responses - Switching Stroop Task
**C(ed_bucket)**	4.318	3.000	1.802	0.149	Total Correct Responses - Switching Stroop Task
**PD**	7.903	1.000	9.898	0.002	Total Correct Responses - Switching Stroop Task
**RBD**	2.520	1.000	3.156	0.078	Total Correct Responses - Switching Stroop Task
**Residual**	117.375	147.000			Total Correct Responses - Switching Stroop Task
**C(age_bucket)**	26.091	4.000	8.006	0.000	Total Correct Responses - Trail Making Task
**C(gender)**	0.896	1.000	1.100	0.296	Total Correct Responses - Trail Making Task
**C(ed_bucket)**	0.359	3.000	0.147	0.931	Total Correct Responses - Trail Making Task
**PD**	1.788	1.000	2.194	0.141	Total Correct Responses - Trail Making Task
**RBD**	1.756	1.000	2.155	0.144	Total Correct Responses - Trail Making Task
**Residual**	119.767	147.000			Total Correct Responses - Trail Making Task
**C(age_bucket)**	11.736	4.000	3.734	0.006	Total Correct Responses - Spatial Span Task
**C(gender)**	0.017	1.000	0.022	0.884	Total Correct Responses - Spatial Span Task
**C(ed_bucket)**	1.117	3.000	0.474	0.701	Total Correct Responses - Spatial Span Task
**PD**	1.883	1.000	2.397	0.124	Total Correct Responses - Spatial Span Task

RBD	0.794	1.000	1.010	0.316	Total Correct Responses - Spatial Span Task
Residual	117.851	150.000			Total Correct Responses - Spatial Span Task
C(age_bucket)	20.377	4.000	6.307	0.000	Total Correct Responses - Verbal Analogies Task
C(gender)	0.216	1.000	0.268	0.606	Total Correct Responses - Verbal Analogies Task
C(ed_bucket)	3.227	3.000	1.332	0.266	Total Correct Responses - Verbal Analogies Task
PD	3.528	1.000	4.368	0.038	Total Correct Responses - Verbal Analogies Task
RBD	6.477	1.000	8.020	0.005	Total Correct Responses - Verbal Analogies Task
Residual	121.146	150.000			Total Correct Responses - Verbal Analogies Task
C(age_bucket)	2.841	4.000	0.894	0.469	Total Words Remembered - Word Definitions Task
C(gender)	0.134	1.000	0.168	0.682	Total Words Remembered - Word Definitions Task
C(ed_bucket)	9.299	3.000	3.899	0.010	Total Words Remembered - Word Definitions Task
PD	3.675	1.000	4.623	0.033	Total Words Remembered - Word Definitions Task
RBD	8.880	1.000	11.171	0.001	Total Words Remembered - Word Definitions Task
Residual	119.240	150.000			Total Words Remembered - Word Definitions Task
C(age_bucket)	9.509	4.000	2.998	0.021	Total Correct Responses - Paired Associates Learning Task
C(gender)	0.001	1.000	0.001	0.978	Total Correct Responses - Paired Associates Learning Task
C(ed_bucket)	2.660	3.000	1.118	0.344	Total Correct Responses - Paired Associates Learning Task
PD	1.303	1.000	1.643	0.202	Total Correct Responses - Paired Associates Learning Task
RBD	0.045	1.000	0.057	0.812	Total Correct Responses - Paired Associates Learning Task
Residual	116.556	147.000			Total Correct Responses - Paired Associates Learning Task
C(age_bucket)	12.979	4.000	3.638	0.007	Percentage Correct Cards - Card Pairs Task
C(gender)	0.491	1.000	0.550	0.459	Percentage Correct Cards - Card Pairs Task
C(ed_bucket)	2.750	3.000	1.028	0.382	Percentage Correct Cards - Card Pairs Task
PD	0.160	1.000	0.180	0.672	Percentage Correct Cards - Card Pairs Task
RBD	0.563	1.000	0.632	0.428	Percentage Correct Cards - Card Pairs Task
Residual	130.214	146.000			Percentage Correct Cards - Card Pairs Task
C(age_bucket)	5.520	4.000	1.615	0.174	Total Achieved - Four Towers Task
C(gender)	0.019	1.000	0.022	0.882	Total Achieved - Four Towers Task
C(ed_bucket)	6.576	3.000	2.565	0.057	Total Achieved - Four Towers Task
PD	1.686	1.000	1.974	0.162	Total Achieved - Four Towers Task
RBD	0.000	1.000	0.000	0.995	Total Achieved - Four Towers Task
Residual	121.326	142.000			Total Achieved - Four Towers Task
C(age_bucket)	12.117	4.000	3.630	0.007	Total Correct Responses - Digit Span Task
C(gender)	0.306	1.000	0.367	0.545	Total Correct Responses - Digit Span Task
C(ed_bucket)	2.374	3.000	0.948	0.419	Total Correct Responses - Digit Span Task
PD	0.000	1.000	0.000	0.987	Total Correct Responses - Digit Span Task
RBD	0.564	1.000	0.676	0.412	Total Correct Responses - Digit Span Task
Residual	125.179	150.000			Total Correct Responses - Digit Span Task
C(age_bucket)	7.947	4.000	2.307	0.061	Total Achieved - Tower of London Task

C(gender)	4.872	1.000	5.657	0.019	Total Achieved - Tower of London Task
C(ed_bucket)	2.657	3.000	1.029	0.382	Total Achieved - Tower of London Task
PD	0.007	1.000	0.008	0.928	Total Achieved - Tower of London Task
RBD	0.466	1.000	0.541	0.463	Total Achieved - Tower of London Task
Residual	128.313	149.000			Total Achieved - Tower of London Task
C(age_bucket)	13.567	4.000	3.869	0.005	Mean Reaction Time - Motor Control Task
C(gender)	3.466	1.000	3.953	0.049	Mean Reaction Time - Motor Control Task
C(ed_bucket)	1.753	3.000	0.667	0.574	Mean Reaction Time - Motor Control Task
PD	0.942	1.000	1.075	0.302	Mean Reaction Time - Motor Control Task
RBD	2.096	1.000	2.391	0.124	Mean Reaction Time - Motor Control Task
Residual	133.255	152.000			Mean Reaction Time - Motor Control Task
C(age_bucket)	5.933	4.000	1.576	0.184	Median Reaction Time - Simple Reaction Time Task
C(gender)	0.066	1.000	0.070	0.791	Median Reaction Time - Simple Reaction Time Task
C(ed_bucket)	0.951	3.000	0.337	0.799	Median Reaction Time - Simple Reaction Time Task
PD	3.673	1.000	3.904	0.050	Median Reaction Time - Simple Reaction Time Task
RBD	3.704	1.000	3.937	0.049	Median Reaction Time - Simple Reaction Time Task
Residual	138.297	147.000			Median Reaction Time - Simple Reaction Time Task
C(age_bucket)	11.760	4.000	3.353	0.012	Total Errors - Picture Completion Task
C(gender)	1.135	1.000	1.295	0.257	Total Errors - Picture Completion Task
C(ed_bucket)	2.549	3.000	0.969	0.409	Total Errors - Picture Completion Task
PD	4.441	1.000	5.064	0.026	Total Errors - Picture Completion Task
RBD	2.005	1.000	2.287	0.133	Total Errors - Picture Completion Task
Residual	126.268	144.000			Total Errors - Picture Completion Task
C(age_bucket)	25.231	4.000	9.356	0.000	Accuracy composite
C(gender)	0.011	1.000	0.017	0.896	Accuracy composite
C(ed_bucket)	4.130	3.000	2.042	0.111	Accuracy composite
PD	8.211	1.000	12.178	0.001	Accuracy composite
RBD	3.655	1.000	5.421	0.021	Accuracy composite
Residual	95.066	141.000			Accuracy composite

Supplementary Table 9. Statistical significance of effect size deficits in the PD and RBD groups for primary measures after the sensitivity analysis presented in FigureS5

	sum_sq	df	F	PR(>F)	task
C(age_bucket)	19.227	4.000	5.678	0.000	Immediate Prospective Memory - Median Reaction Time
C(gender)	4.580	1.000	5.410	0.021	Immediate Prospective Memory - Median Reaction Time
C(ed_bucket)	0.984	3.000	0.387	0.762	Immediate Prospective Memory - Median Reaction Time
PD	1.983	1.000	2.343	0.128	Immediate Prospective Memory - Median Reaction Time
RBD	0.130	1.000	0.153	0.696	Immediate Prospective Memory - Median Reaction Time
Residual	127.835	151.000			Immediate Prospective Memory - Median Reaction Time
C(age_bucket)	23.366	4.000	7.267	0.000	Delayed Prospective Memory - Median Reaction Time

C(gender)	2.788	1.000	3.469	0.065	Delayed Prospective Memory - Median Reaction Time
C(ed_bucket)	3.061	3.000	1.269	0.287	Delayed Prospective Memory - Median Reaction Time
PD	4.687	1.000	5.831	0.017	Delayed Prospective Memory - Median Reaction Time
RBD	0.595	1.000	0.740	0.391	Delayed Prospective Memory - Median Reaction Time
Residual	119.771	149.000			Delayed Prospective Memory - Median Reaction Time
C(age_bucket)	24.833	4.000	7.594	0.000	Manipulations 2D - Median Reaction Time
C(gender)	2.619	1.000	3.204	0.075	Manipulations 2D - Median Reaction Time
C(ed_bucket)	1.300	3.000	0.530	0.662	Manipulations 2D - Median Reaction Time
PD	0.096	1.000	0.118	0.732	Manipulations 2D - Median Reaction Time
RBD	0.005	1.000	0.006	0.939	Manipulations 2D - Median Reaction Time
Residual	123.446	151.000			Manipulations 2D - Median Reaction Time
C(age_bucket)	17.187	4.000	4.909	0.001	Emotion Discrimination - Median Reaction Time
C(gender)	1.029	1.000	1.176	0.280	Emotion Discrimination - Median Reaction Time
C(ed_bucket)	1.376	3.000	0.524	0.666	Emotion Discrimination - Median Reaction Time
PD	4.119	1.000	4.706	0.032	Emotion Discrimination - Median Reaction Time
RBD	1.586	1.000	1.812	0.180	Emotion Discrimination - Median Reaction Time
Residual	132.159	151.000			Emotion Discrimination - Median Reaction Time
C(age_bucket)	19.232	4.000	5.699	0.000	Blocks - Median Reaction Time
C(gender)	0.002	1.000	0.002	0.963	Blocks - Median Reaction Time
C(ed_bucket)	5.270	3.000	2.082	0.105	Blocks - Median Reaction Time
PD	5.399	1.000	6.400	0.012	Blocks - Median Reaction Time
RBD	2.336	1.000	2.769	0.098	Blocks - Median Reaction Time
Residual	126.545	150.000			Blocks - Median Reaction Time
C(age_bucket)	24.351	4.000	7.454	0.000	Target Detection - Mean Reaction Time
C(gender)	2.389	1.000	2.925	0.089	Target Detection - Mean Reaction Time
C(ed_bucket)	0.101	3.000	0.041	0.989	Target Detection - Mean Reaction Time
PD	8.524	1.000	10.436	0.002	Target Detection - Mean Reaction Time
RBD	0.502	1.000	0.615	0.434	Target Detection - Mean Reaction Time
Residual	123.331	151.000			Target Detection - Mean Reaction Time
C(age_bucket)	14.055	4.000	3.944	0.005	Trail Making - Mean Number Reaction Time
C(gender)	3.223	1.000	3.617	0.059	Trail Making - Mean Number Reaction Time
C(ed_bucket)	0.234	3.000	0.088	0.967	Trail Making - Mean Number Reaction Time
PD	0.584	1.000	0.655	0.420	Trail Making - Mean Number Reaction Time
RBD	0.139	1.000	0.156	0.694	Trail Making - Mean Number Reaction Time
Residual	130.974	147.000			Trail Making - Mean Number Reaction Time
C(age_bucket)	27.794	4.000	8.629	0.000	Trail Making - Mean Letter Reaction Time
C(gender)	0.308	1.000	0.383	0.537	Trail Making - Mean Letter Reaction Time
C(ed_bucket)	0.533	3.000	0.221	0.882	Trail Making - Mean Letter Reaction Time
PD	3.216	1.000	3.994	0.048	Trail Making - Mean Letter Reaction Time
RBD	2.680	1.000	3.328	0.070	Trail Making - Mean Letter Reaction Time

**Residual**	118.373	147.000			Trail Making - Mean Letter Reaction Time
**C(age_bucket)**	17.741	4.000	5.152	0.001	Trail Making - Number/Letter Cost
**C(gender)**	0.027	1.000	0.031	0.860	Trail Making - Number/Letter Cost
**C(ed_bucket)**	1.313	3.000	0.508	0.677	Trail Making - Number/Letter Cost
**PD**	2.661	1.000	3.091	0.081	Trail Making - Number/Letter Cost
**RBD**	4.772	1.000	5.544	0.020	Trail Making - Number/Letter Cost
**Residual**	126.538	147.000			Trail Making - Number/Letter Cost
**C(age_bucket)**	24.779	4.000	7.427	0.000	Switching Stroop - Median Reaction Time
**C(gender)**	0.792	1.000	0.950	0.331	Switching Stroop - Median Reaction Time
**C(ed_bucket)**	0.739	3.000	0.295	0.829	Switching Stroop - Median Reaction Time
**PD**	2.398	1.000	2.875	0.092	Switching Stroop - Median Reaction Time
**RBD**	0.400	1.000	0.479	0.490	Switching Stroop - Median Reaction Time
**Residual**	122.605	147.000			Switching Stroop - Median Reaction Time
**C(age_bucket)**	19.835	4.000	6.028	0.000	Switching Stroop - Mean Incongruent Accuracy
**C(gender)**	0.373	1.000	0.454	0.502	Switching Stroop - Mean Incongruent Accuracy
**C(ed_bucket)**	3.495	3.000	1.416	0.240	Switching Stroop - Mean Incongruent Accuracy
**PD**	9.047	1.000	10.997	0.001	Switching Stroop - Mean Incongruent Accuracy
**RBD**	2.117	1.000	2.574	0.111	Switching Stroop - Mean Incongruent Accuracy
**Residual**	120.931	147.000			Switching Stroop - Mean Incongruent Accuracy
**C(age_bucket)**	27.125	4.000	8.319	0.000	Switching Stroop - Median Incongruent Reaction Time
**C(gender)**	0.285	1.000	0.350	0.555	Switching Stroop - Median Incongruent Reaction Time
**C(ed_bucket)**	1.324	3.000	0.541	0.655	Switching Stroop - Median Incongruent Reaction Time
**PD**	3.681	1.000	4.516	0.035	Switching Stroop - Median Incongruent Reaction Time
**RBD**	1.204	1.000	1.477	0.226	Switching Stroop - Median Incongruent Reaction Time
**Residual**	119.833	147.000			Switching Stroop - Median Incongruent Reaction Time
**C(age_bucket)**	7.268	4.000	2.169	0.075	Switching Stroop - Mean Switch Accuracy
**C(gender)**	0.572	1.000	0.683	0.410	Switching Stroop - Mean Switch Accuracy
**C(ed_bucket)**	1.968	3.000	0.783	0.505	Switching Stroop - Mean Switch Accuracy
**PD**	1.545	1.000	1.844	0.177	Switching Stroop - Mean Switch Accuracy
**RBD**	1.684	1.000	2.010	0.158	Switching Stroop - Mean Switch Accuracy
**Residual**	123.167	147.000			Switching Stroop - Mean Switch Accuracy
**C(age_bucket)**	20.678	4.000	5.987	0.000	Switching Stroop - Median Switch Reaction Time
**C(gender)**	0.028	1.000	0.033	0.856	Switching Stroop - Median Switch Reaction Time
**C(ed_bucket)**	1.525	3.000	0.589	0.623	Switching Stroop - Median Switch Reaction Time
**PD**	2.228	1.000	2.581	0.110	Switching Stroop - Median Switch Reaction Time
**RBD**	0.426	1.000	0.493	0.484	Switching Stroop - Median Switch Reaction Time
**Residual**	126.920	147.000			Switching Stroop - Median Switch Reaction Time
**C(age_bucket)**	13.046	4.000	3.653	0.007	Spatial Span - Median Reaction Time
**C(gender)**	0.278	1.000	0.311	0.578	Spatial Span - Median Reaction Time

C(ed_bucket)	4.103	3.000	1.532	0.209	Spatial Span - Median Reaction Time
PD	0.897	1.000	1.005	0.318	Spatial Span - Median Reaction Time
RBD	0.138	1.000	0.155	0.694	Spatial Span - Median Reaction Time
Residual	133.919	150.000			Spatial Span - Median Reaction Time
C(age_bucket)	28.661	4.000	9.213	0.000	Verbal Analogies - Median Reaction Time
C(gender)	1.311	1.000	1.685	0.196	Verbal Analogies - Median Reaction Time
C(ed_bucket)	0.584	3.000	0.250	0.861	Verbal Analogies - Median Reaction Time
PD	5.623	1.000	7.230	0.008	Verbal Analogies - Median Reaction Time
RBD	4.235	1.000	5.445	0.021	Verbal Analogies - Median Reaction Time
Residual	116.654	150.000			Verbal Analogies - Median Reaction Time
C(age_bucket)	19.861	4.000	5.985	0.000	Digit Sapn - Median Reaction Time
C(gender)	2.556	1.000	3.081	0.081	Digit Sapn - Median Reaction Time
C(ed_bucket)	3.924	3.000	1.577	0.197	Digit Sapn - Median Reaction Time
PD	2.855	1.000	3.442	0.066	Digit Sapn - Median Reaction Time
RBD	0.260	1.000	0.314	0.576	Digit Sapn - Median Reaction Time
Residual	124.445	150.000			Digit Sapn - Median Reaction Time
C(age_bucket)	3.449	4.000	0.943	0.441	Word Definitions - Median Reaction Time
C(gender)	0.555	1.000	0.606	0.437	Word Definitions - Median Reaction Time
C(ed_bucket)	2.383	3.000	0.868	0.459	Word Definitions - Median Reaction Time
PD	2.517	1.000	2.751	0.099	Word Definitions - Median Reaction Time
RBD	6.950	1.000	7.597	0.007	Word Definitions - Median Reaction Time
Residual	137.229	150.000			Word Definitions - Median Reaction Time
C(age_bucket)	25.974	4.000	7.785	0.000	PAL - Median Reaction Time
C(gender)	0.002	1.000	0.002	0.965	PAL - Median Reaction Time
C(ed_bucket)	0.611	3.000	0.244	0.865	PAL - Median Reaction Time
PD	3.341	1.000	4.005	0.047	PAL - Median Reaction Time
RBD	0.170	1.000	0.204	0.652	PAL - Median Reaction Time
Residual	122.609	147.000			PAL - Median Reaction Time
C(age_bucket)	21.486	4.000	6.225	0.000	Card Pairs - Median Array Time
C(gender)	0.001	1.000	0.001	0.970	Card Pairs - Median Array Time
C(ed_bucket)	0.715	3.000	0.276	0.842	Card Pairs - Median Array Time
PD	0.762	1.000	0.883	0.349	Card Pairs - Median Array Time
RBD	0.590	1.000	0.684	0.409	Card Pairs - Median Array Time
Residual	125.984	146.000			Card Pairs - Median Array Time
C(age_bucket)	43.843	4.000	16.145	0.000	Picture Completion - Total Time Taken
C(gender)	3.096	1.000	4.560	0.034	Picture Completion - Total Time Taken
C(ed_bucket)	0.791	3.000	0.388	0.762	Picture Completion - Total Time Taken
PD	2.674	1.000	3.939	0.049	Picture Completion - Total Time Taken
RBD	0.015	1.000	0.022	0.881	Picture Completion - Total Time Taken

Residual	97.759	144.000			Picture Completion - Total Time Taken
C(age_bucket)	15.506	4.000	4.348	0.002	Four Towers - Median Reaction Time
C(gender)	0.248	1.000	0.278	0.599	Four Towers - Median Reaction Time
C(ed_bucket)	3.170	3.000	1.185	0.318	Four Towers - Median Reaction Time
PD	0.519	1.000	0.582	0.447	Four Towers - Median Reaction Time
RBD	0.225	1.000	0.253	0.616	Four Towers - Median Reaction Time
Residual	126.607	142.000			Four Towers - Median Reaction Time
C(age_bucket)	7.112	4.000	1.973	0.102	Tower of London - Median Reaction Time
C(gender)	4.461	1.000	4.951	0.028	Tower of London - Median Reaction Time
C(ed_bucket)	0.877	3.000	0.324	0.808	Tower of London - Median Reaction Time
PD	0.514	1.000	0.570	0.451	Tower of London - Median Reaction Time
RBD	0.061	1.000	0.068	0.795	Tower of London - Median Reaction Time
Residual	134.265	149.000			Tower of London - Median Reaction Time
C(age_bucket)	1.807	4.000	0.510	0.728	Motor Control - Mean Euclidean Distance from Target
C(gender)	0.000	1.000	0.000	0.987	Motor Control - Mean Euclidean Distance from Target
C(ed_bucket)	3.582	3.000	1.349	0.261	Motor Control - Mean Euclidean Distance from Target
PD	11.447	1.000	12.931	0.000	Motor Control - Mean Euclidean Distance from Target
RBD	0.717	1.000	0.809	0.370	Motor Control - Mean Euclidean Distance from Target
Residual	134.548	152.000			Motor Control - Mean Euclidean Distance from Target
C(age_bucket)	6.249	4.000	1.663	0.162	Simple Reaction Time - Median Reaction Time
C(gender)	0.340	1.000	0.362	0.548	Simple Reaction Time - Median Reaction Time
C(ed_bucket)	1.313	3.000	0.466	0.707	Simple Reaction Time - Median Reaction Time
PD	3.436	1.000	3.657	0.058	Simple Reaction Time - Median Reaction Time
RBD	2.893	1.000	3.079	0.081	Simple Reaction Time - Median Reaction Time
Residual	138.135	147.000			Simple Reaction Time - Median Reaction Time
C(age_bucket)	39.098	4.000	13.746	0.000	RT composite
C(gender)	2.498	1.000	3.513	0.063	RT composite
C(ed_bucket)	0.261	3.000	0.122	0.947	RT composite
PD	4.089	1.000	5.751	0.018	RT composite
RBD	0.562	1.000	0.790	0.376	RT composite
Residual	99.547	140.000			RT composite

Supplementary Table 10. Statistical significance of effect size deficits in the PD and RBD groups for secondary measures after the sensitivity analysis presented in FigureS5

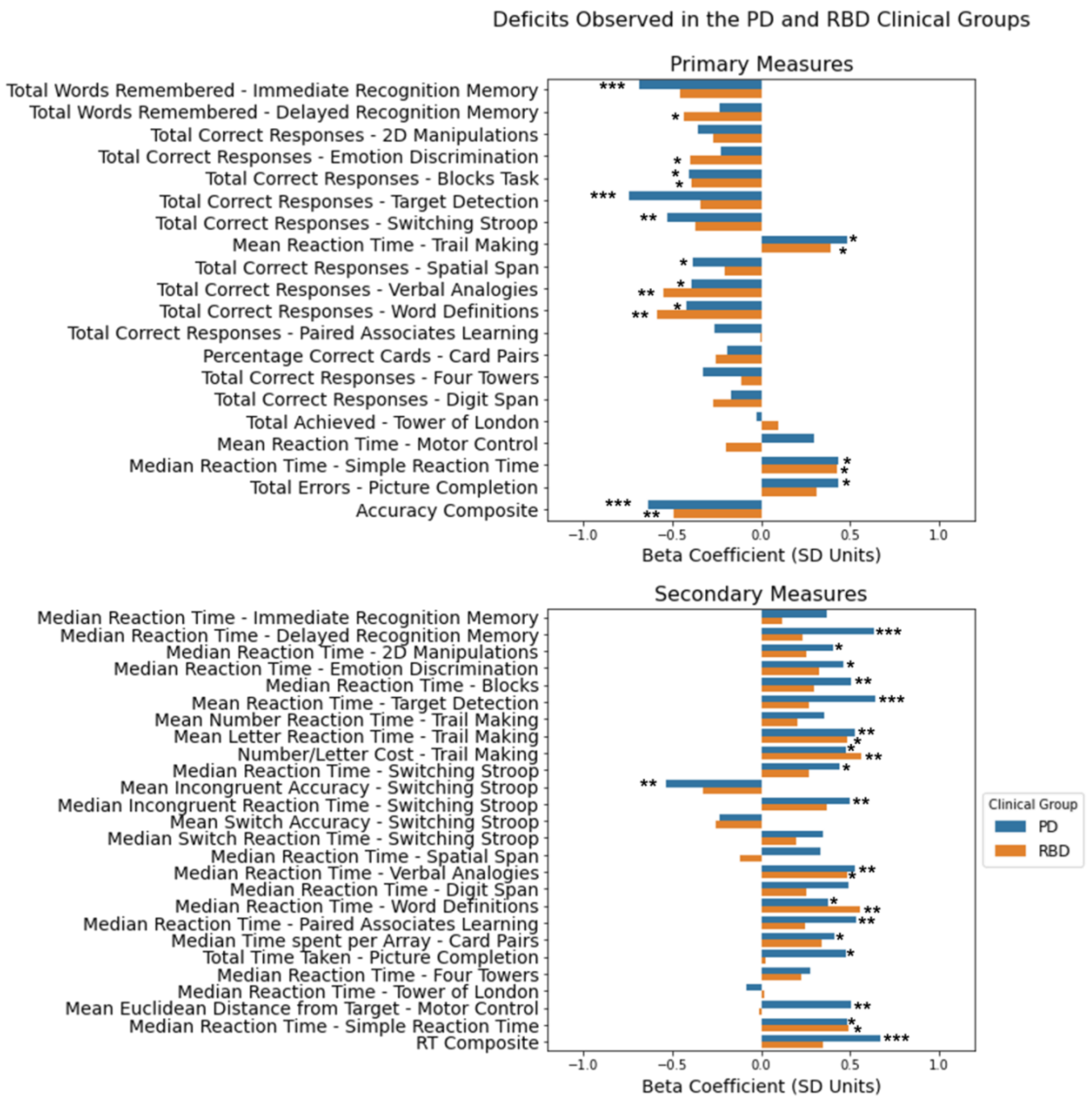

Supplementary Figure 6. Sensitivity analysis – replacing age decade with age, age-squared, age-cubed

	sum_sq	df	F	PR(>F)	task
**PD**	12.475	1.000	14.100	0.000	Total Words Remembered - Immediate Recognition Memory
**RBD**	5.373	1.000	6.073	0.015	Total Words Remembered - Immediate Recognition Memory
**Residual**	140.669	159.000			Total Words Remembered - Immediate Recognition Memory
**PD**	1.493	1.000	1.598	0.208	Total Words Remembered - Delayed Recognition Memory
**RBD**	4.729	1.000	5.061	0.026	Total Words Remembered - Delayed Recognition Memory
**Residual**	146.690	157.000			Total Words Remembered - Delayed Recognition Memory
**PD**	3.407	1.000	3.616	0.059	Total Correct Responses - 2D Manipulations Task
**RBD**	1.880	1.000	1.995	0.160	Total Correct Responses - 2D Manipulations Task
**Residual**	149.788	159.000			Total Correct Responses - 2D Manipulations Task
**PD**	1.345	1.000	1.433	0.233	Total Correct Responses - Emotion Discrimination Task
**RBD**	4.067	1.000	4.331	0.039	Total Correct Responses - Emotion Discrimination Task

Residual	149.314	159.000			Total Correct Responses - Emotion Discrimination Task
PD	4.346	1.000	4.668	0.032	Total Correct Responses - Blocks Task
RBD	3.840	1.000	4.125	0.044	Total Correct Responses - Blocks Task
Residual	147.085	158.000			Total Correct Responses - Blocks Task
PD	14.668	1.000	16.828	0.000	Total Correct Responses - Target Detection Task
RBD	2.997	1.000	3.439	0.066	Total Correct Responses - Target Detection Task
Residual	138.590	159.000			Total Correct Responses - Target Detection Task
PD	7.390	1.000	8.075	0.005	Total Correct Responses - Switching Stroop Task
RBD	3.367	1.000	3.680	0.057	Total Correct Responses - Switching Stroop Task
Residual	141.846	155.000			Total Correct Responses - Switching Stroop Task
PD	6.063	1.000	6.579	0.011	Total Correct Responses - Trail Making Task
RBD	3.710	1.000	4.025	0.047	Total Correct Responses - Trail Making Task
Residual	142.857	155.000			Total Correct Responses - Trail Making Task
PD	3.898	1.000	4.147	0.043	Total Correct Responses - Spatial Span Task
RBD	1.071	1.000	1.140	0.287	Total Correct Responses - Spatial Span Task
Residual	148.511	158.000			Total Correct Responses - Spatial Span Task
PD	4.019	1.000	4.401	0.038	Total Correct Responses - Verbal Analogies Task
RBD	7.755	1.000	8.492	0.004	Total Correct Responses - Verbal Analogies Task
Residual	144.286	158.000			Total Correct Responses - Verbal Analogies Task
PD	4.749	1.000	5.236	0.023	Total Words Remembered - Word Definitions Task
RBD	8.617	1.000	9.502	0.002	Total Words Remembered - Word Definitions Task
Residual	143.297	158.000			Total Words Remembered - Word Definitions Task
PD	1.863	1.000	1.966	0.163	Total Correct Responses - Paired Associates Learning Task
RBD	0.001	1.000	0.001	0.978	Total Correct Responses - Paired Associates Learning Task
Residual	146.894	155.000			Total Correct Responses - Paired Associates Learning Task
PD	0.961	1.000	1.009	0.317	Percentage Correct Cards - Card Pairs Task
RBD	1.647	1.000	1.729	0.190	Percentage Correct Cards - Card Pairs Task
Residual	146.664	154.000			Percentage Correct Cards - Card Pairs Task
PD	2.781	1.000	2.947	0.088	Total Achieved - Four Towers Task
RBD	0.318	1.000	0.337	0.562	Total Achieved - Four Towers Task
Residual	141.572	150.000			Total Achieved - Four Towers Task
PD	0.747	1.000	0.785	0.377	Total Correct Responses - Digit Span Task
RBD	1.844	1.000	1.937	0.166	Total Correct Responses - Digit Span Task
Residual	150.470	158.000			Total Correct Responses - Digit Span Task
PD	0.016	1.000	0.017	0.896	Total Achieved - Tower of London Task
RBD	0.238	1.000	0.247	0.620	Total Achieved - Tower of London Task
Residual	150.971	157.000			Total Achieved - Tower of London Task
PD	2.417	1.000	2.627	0.107	Mean Reaction Time - Motor Control Task

RBD	1.054	1.000	1.146	0.286	Mean Reaction Time - Motor Control Task
Residual	147.205	160.000			Mean Reaction Time - Motor Control Task
PD	4.803	1.000	5.192	0.024	Median Reaction Time - Simple Reaction Time Task
RBD	4.548	1.000	4.917	0.028	Median Reaction Time - Simple Reaction Time Task
Residual	143.375	155.000			Median Reaction Time - Simple Reaction Time Task
PD	4.823	1.000	5.182	0.024	Total Errors - Picture Completion Task
RBD	2.327	1.000	2.500	0.116	Total Errors - Picture Completion Task
Residual	141.469	152.000			Total Errors - Picture Completion Task
PD	10.103	1.000	13.256	0.000	Accuracy Composite
RBD	5.899	1.000	7.740	0.006	Accuracy Composite
Residual	113.557	149.000			Accuracy Composite

Supplementary Table 11. Statistical significance of effect size deficits in the PD and RBD groups for primary measures after the sensitivity analysis presented in FigureS6

	sum_sq	df	F	PR(>F)	task
PD	3.594	1.000	3.821	0.052	Immediate Prospective Memory - Median Reaction Time
RBD	0.362	1.000	0.385	0.536	Immediate Prospective Memory - Median Reaction Time
Residual	149.562	159.000			Immediate Prospective Memory - Median Reaction Time
PD	10.502	1.000	11.740	0.001	Delayed Prospective Memory - Median Reaction Time
RBD	1.363	1.000	1.523	0.219	Delayed Prospective Memory - Median Reaction Time
Residual	140.450	157.000			Delayed Prospective Memory - Median Reaction Time
PD	4.292	1.000	4.578	0.034	Manipulations 2D - Median Reaction Time
RBD	1.609	1.000	1.716	0.192	Manipulations 2D - Median Reaction Time
Residual	149.072	159.000			Manipulations 2D - Median Reaction Time
PD	5.726	1.000	6.173	0.014	Emotion Discrimination - Median Reaction Time
RBD	2.708	1.000	2.919	0.089	Emotion Discrimination - Median Reaction Time
Residual	147.488	159.000			Emotion Discrimination - Median Reaction Time
PD	6.668	1.000	7.230	0.008	Blocks - Median Reaction Time
RBD	2.212	1.000	2.398	0.123	Blocks - Median Reaction Time
Residual	145.728	158.000			Blocks - Median Reaction Time
PD	10.855	1.000	12.130	0.001	Target Detection - Mean Reaction Time
RBD	1.793	1.000	2.004	0.159	Target Detection - Mean Reaction Time
Residual	142.288	159.000			Target Detection - Mean Reaction Time
PD	3.281	1.000	3.479	0.064	Trail Making - Mean Number Reaction Time
RBD	0.997	1.000	1.057	0.305	Trail Making - Mean Number Reaction Time
Residual	146.164	155.000			Trail Making - Mean Number Reaction Time
PD	7.244	1.000	7.970	0.005	Trail Making - Mean Letter Reaction Time
RBD	5.817	1.000	6.400	0.012	Trail Making - Mean Letter Reaction Time

Residual	140.870	155.000			Trail Making - Mean Letter Reaction Time
PD	5.962	1.000	6.585	0.011	Trail Making - Number/Letter Cost
RBD	7.877	1.000	8.701	0.004	Trail Making - Number/Letter Cost
Residual	140.322	155.000			Trail Making - Number/Letter Cost
PD	5.118	1.000	5.497	0.020	Switching Stroop - Median Reaction Time
RBD	1.757	1.000	1.887	0.171	Switching Stroop - Median Reaction Time
Residual	144.305	155.000			Switching Stroop - Median Reaction Time
PD	7.461	1.000	8.149	0.005	Switching Stroop - Mean Incongruent Accuracy
RBD	2.735	1.000	2.987	0.086	Switching Stroop - Mean Incongruent Accuracy
Residual	141.931	155.000			Switching Stroop - Mean Incongruent Accuracy
PD	6.414	1.000	6.967	0.009	Switching Stroop - Median Incongruent Reaction Time
RBD	3.342	1.000	3.630	0.059	Switching Stroop - Median Incongruent Reaction Time
Residual	142.711	155.000			Switching Stroop - Median Incongruent Reaction Time
PD	1.427	1.000	1.500	0.223	Switching Stroop - Mean Switch Accuracy
RBD	1.631	1.000	1.715	0.192	Switching Stroop - Mean Switch Accuracy
Residual	147.444	155.000			Switching Stroop - Mean Switch Accuracy
PD	3.108	1.000	3.292	0.072	Switching Stroop - Median Switch Reaction Time
RBD	0.923	1.000	0.977	0.324	Switching Stroop - Median Switch Reaction Time
Residual	146.334	155.000			Switching Stroop - Median Switch Reaction Time
PD	2.858	1.000	3.086	0.081	Spatial Span - Median Reaction Time
RBD	0.353	1.000	0.381	0.538	Spatial Span - Median Reaction Time
Residual	146.342	158.000			Spatial Span - Median Reaction Time
PD	7.218	1.000	7.928	0.005	Verbal Analogies - Median Reaction Time
RBD	5.858	1.000	6.433	0.012	Verbal Analogies - Median Reaction Time
Residual	143.867	158.000			Verbal Analogies - Median Reaction Time
PD	6.350	1.000	6.870	0.010	Digit Span - Median Reaction Time
RBD	1.580	1.000	1.710	0.193	Digit Span - Median Reaction Time
Residual	146.045	158.000			Digit Span - Median Reaction Time
PD	3.729	1.000	4.081	0.045	Word Definitions - Median Reaction Time
RBD	7.789	1.000	8.524	0.004	Word Definitions - Median Reaction Time
Residual	144.369	158.000			Word Definitions - Median Reaction Time
PD	7.451	1.000	8.137	0.005	PAL - Median Reaction Time
RBD	1.539	1.000	1.681	0.197	PAL - Median Reaction Time
Residual	141.930	155.000			PAL - Median Reaction Time
PD	4.462	1.000	4.787	0.030	Card Pairs - Median Array Time
RBD	2.870	1.000	3.079	0.081	Card Pairs - Median Array Time
Residual	143.538	154.000			Card Pairs - Median Array Time
PD	5.825	1.000	6.380	0.013	Picture Completion - Total Time Taken

RBD	0.013	1.000	0.014	0.906	Picture Completion - Total Time Taken
Residual	138.788	152.000			Picture Completion - Total Time Taken
PD	1.963	1.000	2.069	0.152	Four Towers - Median Reaction Time
RBD	1.253	1.000	1.321	0.252	Four Towers - Median Reaction Time
Residual	142.322	150.000			Four Towers - Median Reaction Time
PD	0.178	1.000	0.185	0.668	Tower of London - Median Reaction Time
RBD	0.005	1.000	0.005	0.943	Tower of London - Median Reaction Time
Residual	151.120	157.000			Tower of London - Median Reaction Time
PD	6.850	1.000	7.575	0.007	Motor Control - Mean Euclidean Distance from Target
RBD	0.002	1.000	0.002	0.961	Motor Control - Mean Euclidean Distance from Target
Residual	144.674	160.000			Motor Control - Mean Euclidean Distance from Target
PD	6.003	1.000	6.565	0.011	Simple Reaction Time - Median Reaction Time
RBD	5.893	1.000	6.445	0.012	Simple Reaction Time - Median Reaction Time
Residual	141.726	155.000			Simple Reaction Time - Median Reaction Time
PD	11.138	1.000	12.920	0.000	RT composite
RBD	2.856	1.000	3.313	0.071	RT composite
Residual	127.580	148.000			RT composite

Supplementary Table 12. Statistical significance of effect size deficits in the PD and RBD groups for secondary measures after the sensitivity analysis presented in FigureS6

task	GROUP	beta_original	Beta_demographic_sensitivity	beta_age_curve
Total Words Remembered - Immediate Recognition Memory	PD	-0.676	-0.744	-0.683
Total Words Remembered - Immediate Recognition Memory	RBD	-0.447	-0.508	-0.457
Total Words Remembered - Delayed Recognition Memory	PD	-0.227	-0.246	-0.238
Total Words Remembered - Delayed Recognition Memory	RBD	-0.356	-0.397	-0.433
Total Correct Responses - 2D Manipulations	PD	-0.313	-0.327	-0.357
Total Correct Responses - 2D Manipulations	RBD	-0.195	-0.196	-0.270
Total Correct Responses - Emotion Discrimination	PD	-0.231	-0.252	-0.224
Total Correct Responses - Emotion Discrimination	RBD	-0.338	-0.391	-0.398
Total Correct Responses - Blocks Task	PD	-0.380	-0.394	-0.405
Total Correct Responses - Blocks Task	RBD	-0.360	-0.375	-0.389
Total Correct Responses - Target Detection	PD	-0.728	-0.713	-0.740
Total Correct Responses - Target Detection	RBD	-0.297	-0.234	-0.342
Total Correct Responses - Switching Stroop	PD	-0.529	-0.586	-0.530
Total Correct Responses - Switching Stroop	RBD	-0.323	-0.338	-0.367
Mean Reaction Time - Trail Making	PD	0.418	0.279	0.480

Mean Reaction Time - Trail Making	RBD	0.290	0.282	0.386
Total Correct Responses - Spatial Span	PD	-0.340	-0.282	-0.384
Total Correct Responses - Spatial Span	RBD	-0.176	-0.189	-0.205
Total Correct Responses - Verbal Analogies	PD	-0.350	-0.386	-0.390
Total Correct Responses - Verbal Analogies	RBD	-0.534	-0.540	-0.552
Total Correct Responses - Word Definitions	PD	-0.413	-0.394	-0.424
Total Correct Responses - Word Definitions	RBD	-0.572	-0.632	-0.582
Total Correct Responses - Paired Associates Learning	PD	-0.239	-0.238	-0.266
Total Correct Responses - Paired Associates Learning	RBD	0.012	0.045	-0.005
Percentage Correct Cards - Card Pairs	PD	-0.120	-0.083	-0.191
Percentage Correct Cards - Card Pairs	RBD	-0.180	-0.161	-0.258
Total Correct Responses - Four Towers	PD	-0.246	-0.274	-0.330
Total Correct Responses - Four Towers	RBD	-0.050	-0.001	-0.115
Total Correct Responses - DigitSpan	PD	-0.102	-0.003	-0.168
Total Correct Responses - DigitSpan	RBD	-0.161	-0.159	-0.269
Total Achieved - Tower of London	PD	0.001	-0.017	-0.025
Total Achieved - Tower of London	RBD	0.153	0.146	0.097
Mean Reaction Time - Motor Control	PD	0.236	0.199	0.299
Mean Reaction Time - Motor Control	RBD	-0.276	-0.305	-0.202
Median Reaction Time - Simple Reaction Time	PD	0.408	0.402	0.430
Median Reaction Time - Simple Reaction Time	RBD	0.403	0.410	0.427
Total Errors - Picture Completion	PD	0.444	0.442	0.430
Total Errors - Picture Completion	RBD	0.274	0.306	0.308
Accuracy Composite	PD	-0.645	-0.607	-0.632
Accuracy Composite	RBD	-0.445	-0.420	-0.496

Supplementary Table 13. Beta coefficients changes across models for primary measures

task	GROUP	beta_original	beta_demographic_sensitivity	beta_age_curve
Median Reaction Time - Immediate Recognition Memory	PD	0.309	0.289	0.366
Median Reaction Time - Immediate Recognition Memory	RBD	0.064	0.076	0.119
Median Reaction Time - Delayed Recognition Memory	PD	0.554	0.445	0.630
Median Reaction Time - Delayed Recognition Memory	RBD	0.139	0.165	0.233
Median Reaction Time - 2D Manipulations	PD	0.267	0.064	0.400
Median Reaction Time - 2D Manipulations	RBD	0.160	-0.015	0.250
Median Reaction Time - Emotion Discrimination	PD	0.408	0.416	0.463
Median Reaction Time - Emotion Discrimination	RBD	0.278	0.266	0.325
Median Reaction Time - Blocks	PD	0.481	0.478	0.502
Median Reaction Time - Blocks	RBD	0.235	0.324	0.295

Mean Reaction Time - Target Detection	PD	0.601	0.599	0.637
Mean Reaction Time - Target Detection	RBD	0.204	0.150	0.264
Mean Number Reaction Time - Trail Making	PD	0.282	0.159	0.353
Mean Number Reaction Time - Trail Making	RBD	0.120	0.079	0.200
Mean Letter Reaction Time - Trail Making	PD	0.464	0.374	0.525
Mean Letter Reaction Time - Trail Making	RBD	0.377	0.349	0.483
Number/Letter Cost - Trail Making	PD	0.445	0.340	0.476
Number/Letter Cost - Trail Making	RBD	0.432	0.465	0.562
Median Reaction Time - Switching Stroop	PD	0.416	0.323	0.441
Median Reaction Time - Switching Stroop	RBD	0.213	0.135	0.265
Mean Incongruent Accuracy - Switching Stroop	PD	-0.538	-0.627	-0.533
Mean Incongruent Accuracy - Switching Stroop	RBD	-0.283	-0.310	-0.331
Median Incongruent Reaction Time - Switching Stroop	PD	0.492	0.400	0.494
Median Incongruent Reaction Time - Switching Stroop	RBD	0.327	0.234	0.366
Mean Switch Accuracy - Switching Stroop	PD	-0.227	-0.259	-0.233
Mean Switch Accuracy - Switching Stroop	RBD	-0.235	-0.277	-0.256
Median Switch Reaction Time - Switching Stroop	PD	0.328	0.311	0.344
Median Switch Reaction Time - Switching Stroop	RBD	0.172	0.139	0.192
Median Reaction Time - Spatial Span	PD	0.265	0.195	0.329
Median Reaction Time - Spatial Span	RBD	-0.132	-0.079	-0.118
Median Reaction Time - Verbal Analogies	PD	0.489	0.487	0.522
Median Reaction Time - Verbal Analogies	RBD	0.427	0.436	0.480
Median Reaction Time - Word Definitions	PD	0.367	0.326	0.375
Median Reaction Time - Word Definitions	RBD	0.556	0.559	0.554
Median Reaction Time - Paired Associates Learning	PD	0.457	0.381	0.533
Median Reaction Time - Paired Associates Learning	RBD	0.177	0.088	0.248
Median Time spent per Array - Card Pairs	PD	0.290	0.182	0.412
Median Time spent per Array - Card Pairs	RBD	0.229	0.165	0.341
Total Time Taken - Picture Completion	PD	0.372	0.343	0.473
Total Time Taken - Picture Completion	RBD	-0.099	-0.027	0.023
Median Reaction Time - Four Towers	PD	0.216	0.152	0.277
Median Reaction Time - Four Towers	RBD	0.134	0.104	0.227
Median Reaction Time - Digit Span	PD	0.449	0.347	0.490
Median Reaction Time - Digit Span	RBD	0.268	0.108	0.249
Median Reaction Time - Tower of London	PD	-0.124	-0.147	-0.082
Median Reaction Time - Tower of London	RBD	-0.051	0.053	0.014
Mean Euclidean Distance from Target - Motor Control	PD	0.430	0.692	0.503
Mean Euclidean Distance from Target - Motor Control	RBD	0.027	0.178	-0.009

Median Reaction Time - Simple Reaction Time	PD	0.445	0.389	0.481
Median Reaction Time - Simple Reaction Time	RBD	0.440	0.363	0.486
RT composite	PD	0.606	0.431	0.668
RT composite	RBD	0.227	0.145	0.347

Supplementary Table 14. Beta coefficients changes across models for secondary measures

2. Estimating age-related deficits in normative data

Task	50s (N participants)	60s (N participants)	70s (N participants)	80s (N participants)
Four Towers	36,404	31,908	8,813	925
Pairs Associate Learning	2,427	2,321	807	91
Simple Reaction Time	2,676	2,480	829	61
Tower Of London	116,966	93,455	26,040	2,694
Clocks	117,543	94,192	26,358	2,732
Card Pais	4,371	4,298	1,433	157
Digit Span	116,184	92,539	25,549	2,573
Emotion Discrimination	82,127	59,292	14,181	1,404
2D manipulations	115,047	91,643	25,330	2,593
Motor Control	4,075	2,598	794	66
Picture Completion	1,713	1,877	564	67
Recognition Memory - delayed	26,806	22,432	5,337	563
Recognition Memory - immediate	27,523	23,254	5,698	596
Spatial Span	115,871	92,383	25,574	2,603
Switching Stroop	4,305	4,277	1,410	151
Target Detection	115,410	91,922	25,414	2,600
Trail Making	4,823	4,629	1,632	144
Verbal Analogies	115,840	92,382	25,615	2,634
Word Definitions	115,359	91,932	25,435	2,607

Supplementary Table 15. Number of participants in the normative data in certain age groups

Supplementary Model outputs including standard errors associated with each age decade per task for PRIMARY MEASURES

Task: motorControl

```
                    OLS Regression Results
==============================================================================
Dep. Variable:            value_z   R-squared:                       0.029
Model:                        OLS   Adj. R-squared:                  0.029
Method:             Least Squares   F-statistic:                     75.77
Date:            Tue, 09 Jan 2024   Prob (F-statistic):           2.79e-48
Time:                    21:34:50   Log-Likelihood:                -10577.
No. Observations:            7533   AIC:                         2.116e+04
Df Residuals:                7529   BIC:                         2.119e+04
Df Model:                       3
Covariance Type:        nonrobust
==============================================================================
              coef    std err          t      P>|t|      [0.025      0.975]
------------------------------------------------------------------------------
Intercept    -0.1446      0.015     -9.365      0.000      -0.175      -0.114
decade60s     0.2583      0.025     10.441      0.000       0.210       0.307
decade70s     0.4732      0.038     12.377      0.000       0.398       0.548
decade80s     0.6405      0.122      5.238      0.000       0.401       0.880
==============================================================================
Omnibus:                 1244.885   Durbin-Watson:                   1.746
Prob(Omnibus):              0.000   Jarque-Bera (JB):             2098.005
Skew:                       1.093   Prob(JB):                         0.00
Kurtosis:                   4.380   Cond. No.                         11.6
==============================================================================
```

Task: recognitionMemory_immediate

```
                    OLS Regression Results
==============================================================================
Dep. Variable:            value_z   R-squared:                       0.008
Model:                        OLS   Adj. R-squared:                  0.008
Method:             Least Squares   F-statistic:                     158.7
Date:            Tue, 09 Jan 2024   Prob (F-statistic):          1.95e-102
Time:                    21:34:50   Log-Likelihood:                -80743.
No. Observations:           57071   AIC:                         1.615e+05
Df Residuals:               57067   BIC:                         1.615e+05
Df Model:                       3
Covariance Type:        nonrobust
```

```
==============================================================================
===
             coef    std err          t      P>|t|      [0.025      0.975]
------------------------------------------------------------------------------
Intercept      0.0804      0.006     13.399      0.000       0.069       0.092
decade60s     -0.1261      0.009    -14.218      0.000      -0.144      -0.109
decade70s     -0.2393      0.014    -16.507      0.000      -0.268      -0.211
decade80s     -0.4938      0.041    -11.976      0.000      -0.575      -0.413
==============================================================================
===
Omnibus:                    13323.325   Durbin-Watson:                   1.194
Prob(Omnibus):                  0.000   Jarque-Bera (JB):            34833.424
Skew:                          -1.265   Prob(JB):                         0.00
Kurtosis:                       5.872   Cond. No.                         10.9
==============================================================================
===
```

Task: recognitionMemory_delayed

```
                            OLS Regression Results
==============================================================================
===
Dep. Variable:                value_z   R-squared:                       0.009
Model:                            OLS   Adj. R-squared:                  0.009
Method:                 Least Squares   F-statistic:                     173.2
Date:                Tue, 09 Jan 2024   Prob (F-statistic):         8.60e-112
Time:                        21:34:50   Log-Likelihood:                -77979.
No. Observations:               55138   AIC:                         1.560e+05
Df Residuals:                   55134   BIC:                         1.560e+05
Df Model:                           3
Covariance Type:            nonrobust
==============================================================================
===
             coef    std err          t      P>|t|      [0.025      0.975]
------------------------------------------------------------------------------
Intercept      0.0883      0.006     14.518      0.000       0.076       0.100
decade60s     -0.1460      0.009    -16.214      0.000      -0.164      -0.128
decade70s     -0.2438      0.015    -16.340      0.000      -0.273      -0.215
decade80s     -0.5141      0.042    -12.129      0.000      -0.597      -0.431
==============================================================================
===
Omnibus:                     5796.952   Durbin-Watson:                   1.167
Prob(Omnibus):                  0.000   Jarque-Bera (JB):             8368.337
Skew:                          -0.814   Prob(JB):                         0.00
Kurtosis:                       3.997   Cond. No.                         11.0
==============================================================================
===
```

```
Task: targetDetection
                    OLS Regression Results
==============================================================================
===
Dep. Variable:            value_z   R-squared:                  0.048
Model:                        OLS   Adj. R-squared:             0.048
Method:             Least Squares   F-statistic:                3988.
Date:            Tue, 09 Jan 2024   Prob (F-statistic):          0.00
Time:                    21:34:51   Log-Likelihood:       -3.2811e+05
No. Observations:          235346   AIC:                    6.562e+05
Df Residuals:              235342   BIC:                    6.563e+05
Df Model:                       3
Covariance Type:        nonrobust
==============================================================================
===
              coef    std err          t      P>|t|      [0.025      0.975]
------------------------------------------------------------------------------
Intercept     0.1953      0.003     68.017      0.000       0.190       0.201
decade60s    -0.3065      0.004    -71.080      0.000      -0.315      -0.298
decade70s    -0.5953      0.007    -88.075      0.000      -0.609      -0.582
decade80s    -1.0225      0.019    -52.853      0.000      -1.060      -0.985
==============================================================================
===
Omnibus:                15931.206   Durbin-Watson:              1.313
Prob(Omnibus):              0.000   Jarque-Bera (JB):       19437.562
Skew:                      -0.704   Prob(JB):                    0.00
Kurtosis:                   2.981   Cond. No.                    10.5
==============================================================================
===

Task: emotionDiscrim
                    OLS Regression Results
==============================================================================
===
Dep. Variable:            value_z   R-squared:                  0.010
Model:                        OLS   Adj. R-squared:             0.010
Method:             Least Squares   F-statistic:                545.9
Date:            Tue, 09 Jan 2024   Prob (F-statistic):          0.00
Time:                    21:34:51   Log-Likelihood:       -2.2196e+05
No. Observations:          157004   AIC:                    4.439e+05
Df Residuals:              157000   BIC:                    4.440e+05
Df Model:                       3
Covariance Type:        nonrobust
==============================================================================
===
              coef    std err          t      P>|t|      [0.025      0.975]
------------------------------------------------------------------------------
```

```
Intercept     0.0752      0.003     21.657      0.000       0.068       0.082
decade60s    -0.1129      0.005    -21.057      0.000      -0.123      -0.102
decade70s    -0.3058      0.009    -33.800      0.000      -0.324      -0.288
decade80s    -0.5512      0.027    -20.587      0.000      -0.604      -0.499
==============================================================================
===
Omnibus:                20819.848   Durbin-Watson:                   1.652
Prob(Omnibus):              0.000   Jarque-Bera (JB):            38901.907
Skew:                      -0.858   Prob(JB):                         0.00
Kurtosis:                   4.732   Cond. No.                         11.6
==============================================================================
===

Task: manipulations2D
                            OLS Regression Results
==============================================================================
===
Dep. Variable:                value_z   R-squared:                       0.077
Model:                            OLS   Adj. R-squared:                  0.077
Method:                 Least Squares   F-statistic:                     6535.
Date:                Tue, 09 Jan 2024   Prob (F-statistic):               0.00
Time:                        21:34:51   Log-Likelihood:            -3.2349e+05
No. Observations:              234613   AIC:                         6.470e+05
Df Residuals:                  234609   BIC:                         6.470e+05
Df Model:                           3
Covariance Type:            nonrobust
==============================================================================
===
                 coef    std err          t      P>|t|      [0.025      0.975]
------------------------------------------------------------------------------
Intercept     0.2568      0.003     90.676      0.000       0.251       0.262
decade60s    -0.4179      0.004    -98.253      0.000      -0.426      -0.410
decade70s    -0.7531      0.007   -112.942      0.000      -0.766      -0.740
decade80s    -1.1104      0.019    -58.203      0.000      -1.148      -1.073
==============================================================================
===
Omnibus:                 5965.628   Durbin-Watson:                   1.191
Prob(Omnibus):              0.000   Jarque-Bera (JB):             7665.861
Skew:                       0.316   Prob(JB):                         0.00
Kurtosis:                   3.619   Cond. No.                         10.5
==============================================================================
===

Task: digitSpan
                            OLS Regression Results
==============================================================================
===
```

```
Dep. Variable:             value_z   R-squared:                   0.006
Model:                        OLS   Adj. R-squared:              0.006
Method:               Least Squares   F-statistic:                 481.0
Date:             Tue, 09 Jan 2024   Prob (F-statistic):        1.27e-311
Time:                    21:34:51   Log-Likelihood:          -3.3535e+05
No. Observations:          236845   AIC:                     6.707e+05
Df Residuals:              236841   BIC:                     6.707e+05
Df Model:                       3
Covariance Type:          nonrobust
===============================================================================
===
             coef    std err          t      P>|t|      [0.025      0.975]
-------------------------------------------------------------------------------
Intercept      0.0634      0.003     21.684      0.000       0.058       0.069
decade60s     -0.0952      0.004    -21.662      0.000      -0.104      -0.087
decade70s     -0.1960      0.007    -28.452      0.000      -0.210      -0.183
decade80s     -0.4696      0.020    -23.634      0.000      -0.509      -0.431
===============================================================================
===
Omnibus:                101617.651   Durbin-Watson:                 1.150
Prob(Omnibus):                0.000   Jarque-Bera (JB):        1150938.400
Skew:                         1.759   Prob(JB):                       0.00
Kurtosis:                    13.210   Cond. No.                       10.6
===============================================================================
===

Task: spatialSpan
                            OLS Regression Results
===============================================================================
===
Dep. Variable:             value_z   R-squared:                   0.047
Model:                        OLS   Adj. R-squared:              0.047
Method:               Least Squares   F-statistic:                 3867.
Date:             Tue, 09 Jan 2024   Prob (F-statistic):             0.00
Time:                    21:34:52   Log-Likelihood:          -3.2982e+05
No. Observations:          236431   AIC:                     6.596e+05
Df Residuals:              236427   BIC:                     6.597e+05
Df Model:                       3
Covariance Type:          nonrobust
===============================================================================
===
             coef    std err          t      P>|t|      [0.025      0.975]
-------------------------------------------------------------------------------
Intercept      0.2013      0.003     70.173      0.000       0.196       0.207
decade60s     -0.3306      0.004    -76.772      0.000      -0.339      -0.322
decade70s     -0.5761      0.007    -85.412      0.000      -0.589      -0.563
decade80s     -0.8876      0.019    -45.868      0.000      -0.925      -0.850
```

```
==============================================================================
===
Omnibus:                27522.498   Durbin-Watson:                   1.242
Prob(Omnibus):              0.000   Jarque-Bera (JB):           130097.218
Skew:                       0.483   Prob(JB):                         0.00
Kurtosis:                   6.503   Cond. No.                         10.6
==============================================================================
===
```

Task: blocks

```
                            OLS Regression Results
==============================================================================
===
Dep. Variable:                value_z   R-squared:                       0.039
Model:                            OLS   Adj. R-squared:                  0.039
Method:                 Least Squares   F-statistic:                     3280.
Date:                Tue, 09 Jan 2024   Prob (F-statistic):               0.00
Time:                        21:34:52   Log-Likelihood:            -3.3689e+05
No. Observations:              240825   AIC:                         6.738e+05
Df Residuals:                  240821   BIC:                         6.738e+05
Df Model:                           3
Covariance Type:            nonrobust
==============================================================================
===
                 coef    std err          t      P>|t|      [0.025      0.975]
------------------------------------------------------------------------------
Intercept      0.1806      0.003     63.183      0.000       0.175       0.186
decade60s     -0.2864      0.004    -66.819      0.000      -0.295      -0.278
decade70s     -0.5414      0.007    -81.042      0.000      -0.554      -0.528
decade80s     -0.8252      0.019    -43.499      0.000      -0.862      -0.788
==============================================================================
===
Omnibus:                15522.044   Durbin-Watson:                   1.225
Prob(Omnibus):              0.000   Jarque-Bera (JB):            18681.776
Skew:                      -0.668   Prob(JB):                         0.00
Kurtosis:                   3.273   Cond. No.                         10.4
==============================================================================
===
```

Task: TOL

```
                            OLS Regression Results
==============================================================================
===
Dep. Variable:                value_z   R-squared:                       0.018
Model:                            OLS   Adj. R-squared:                  0.018
Method:                 Least Squares   F-statistic:                     1484.
Date:                Tue, 09 Jan 2024   Prob (F-statistic):               0.00
```

```
Time:                  21:34:52   Log-Likelihood:          -3.3714e+05
No. Observations:            239155   AIC:                    6.743e+05
Df Residuals:              239151   BIC:                   6.743e+05
Df Model:                    3
Covariance Type:          nonrobust
==============================================================================
===
             coef    std err          t      P>|t|      [0.025      0.975]
------------------------------------------------------------------------------
Intercept      0.1171      0.003     40.409      0.000       0.111       0.123
decade60s     -0.1768      0.004    -40.666      0.000      -0.185      -0.168
decade70s     -0.3767      0.007    -55.480      0.000      -0.390      -0.363
decade80s     -0.6191      0.019    -32.066      0.000      -0.657      -0.581
==============================================================================
===
Omnibus:                 19890.205   Durbin-Watson:                1.247
Prob(Omnibus):                0.000   Jarque-Bera (JB):          19327.141
Skew:                        -0.639   Prob(JB):                       0.00
Kurtosis:                     2.445   Cond. No.                       10.4
==============================================================================
===

Task: verbalAnalogies
                            OLS Regression Results
==============================================================================
===
Dep. Variable:               value_z   R-squared:                       0.017
Model:                           OLS   Adj. R-squared:                  0.017
Method:                 Least Squares   F-statistic:                     1331.
Date:               Tue, 09 Jan 2024   Prob (F-statistic):               0.00
Time:                  21:34:52   Log-Likelihood:          -3.3356e+05
No. Observations:            236471   AIC:                    6.671e+05
Df Residuals:              236467   BIC:                   6.672e+05
Df Model:                    3
Covariance Type:          nonrobust
==============================================================================
===
             coef    std err          t      P>|t|      [0.025      0.975]
------------------------------------------------------------------------------
Intercept      0.0999      0.003     34.277      0.000       0.094       0.106
decade60s     -0.1367      0.004    -31.241      0.000      -0.145      -0.128
decade70s     -0.3571      0.007    -52.149      0.000      -0.370      -0.344
decade80s     -0.7009      0.020    -35.867      0.000      -0.739      -0.663
==============================================================================
===
Omnibus:                   620.657   Durbin-Watson:                1.127
Prob(Omnibus):                0.000   Jarque-Bera (JB):            654.047
```

```
Skew:                    -0.104   Prob(JB):                 9.45e-143
Kurtosis:                 3.153   Cond. No.                      10.5
===========================================================================
===

Task: wordDefinitions
                  OLS Regression Results
===========================================================================
===
Dep. Variable:              value_z   R-squared:                    0.032
Model:                          OLS   Adj. R-squared:               0.032
Method:               Least Squares   F-statistic:                  2604.
Date:              Tue, 09 Jan 2024   Prob (F-statistic):            0.00
Time:                      21:34:52   Log-Likelihood:         -3.3008e+05
No. Observations:            235333   AIC:                      6.602e+05
Df Residuals:                235329   BIC:                      6.602e+05
Df Model:                         3
Covariance Type:          nonrobust
===========================================================================
===
             coef    std err          t      P>|t|      [0.025      0.975]
------------------------------------------------------------------------------
Intercept    -0.1796      0.003    -61.990      0.000      -0.185      -0.174
decade60s     0.3268      0.004     75.130      0.000       0.318       0.335
decade70s     0.4294      0.007     63.007      0.000       0.416       0.443
decade80s     0.4960      0.019     25.455      0.000       0.458       0.534
===========================================================================
===
Omnibus:                  49783.666   Durbin-Watson:                1.355
Prob(Omnibus):                0.000   Jarque-Bera (JB):        118655.904
Skew:                        -1.187   Prob(JB):                      0.00
Kurtosis:                     5.543   Cond. No.                      10.5
===========================================================================
===

Task: fourTowers
                  OLS Regression Results
===========================================================================
===
Dep. Variable:              value_z   R-squared:                    0.019
Model:                          OLS   Adj. R-squared:               0.019
Method:               Least Squares   F-statistic:                  511.8
Date:              Tue, 09 Jan 2024   Prob (F-statistic):            0.00
Time:                      21:34:53   Log-Likelihood:         -1.0999e+05
No. Observations:             78050   AIC:                      2.200e+05
Df Residuals:                 78046   BIC:                      2.200e+05
Df Model:                         3
```

```
Covariance Type:          nonrobust
===========================================================================
===
              coef    std err          t      P>|t|      [0.025      0.975]
------------------------------------------------------------------------------
Intercept      0.1234      0.005     23.783      0.000       0.113       0.134
decade60s     -0.1754      0.008    -23.097      0.000      -0.190      -0.161
decade70s     -0.3941      0.012    -33.517      0.000      -0.417      -0.371
decade80s     -0.6106      0.033    -18.520      0.000      -0.675      -0.546
===========================================================================
===
Omnibus:                     9200.249   Durbin-Watson:                   0.936
Prob(Omnibus):                  0.000   Jarque-Bera (JB):            12912.488
Skew:                          -0.994   Prob(JB):                         0.00
Kurtosis:                       3.124   Cond. No.                         10.3
===========================================================================
===

Task: SRT
                            OLS Regression Results
===========================================================================
===
Dep. Variable:                value_z   R-squared:                       0.040
Model:                            OLS   Adj. R-squared:                  0.040
Method:                 Least Squares   F-statistic:                     84.47
Date:                Tue, 09 Jan 2024   Prob (F-statistic):           1.55e-53
Time:                        21:34:53   Log-Likelihood:                -8454.7
No. Observations:                6046   AIC:                         1.692e+04
Df Residuals:                    6042   BIC:                         1.694e+04
Df Model:                           3
Covariance Type:            nonrobust
===========================================================================
===
              coef    std err          t      P>|t|      [0.025      0.975]
------------------------------------------------------------------------------
Intercept     -0.1903      0.019    -10.047      0.000      -0.227      -0.153
decade60s      0.2659      0.027      9.734      0.000       0.212       0.319
decade70s      0.5159      0.039     13.245      0.000       0.440       0.592
decade80s      1.0434      0.127      8.222      0.000       0.795       1.292
===========================================================================
===
Omnibus:                     1771.310   Durbin-Watson:                   0.758
Prob(Omnibus):                  0.000   Jarque-Bera (JB):             6437.964
Skew:                           1.437   Prob(JB):                         0.00
Kurtosis:                       7.159   Cond. No.                         11.1
===========================================================================
===
```

Task: switchingStroop

OLS Regression Results

==

Dep. Variable:	value_z	R-squared:	0.064
Model:	OLS	Adj. R-squared:	0.064
Method:	Least Squares	F-statistic:	232.0
Date:	Tue, 09 Jan 2024	Prob (F-statistic):	1.29e-145
Time:	21:34:53	Log-Likelihood:	-14056.
No. Observations:	10143	AIC:	2.812e+04
Df Residuals:	10139	BIC:	2.815e+04
Df Model:	3		
Covariance Type:	nonrobust		

==

	coef	std err	t	P>|t|	[0.025	0.975]
Intercept	0.2725	0.015	18.482	0.000	0.244	0.301
decade60s	-0.4021	0.021	-19.249	0.000	-0.443	-0.361
decade70s	-0.6377	0.030	-21.482	0.000	-0.696	-0.580
decade80s	-0.9633	0.080	-12.026	0.000	-1.120	-0.806

==

Omnibus:	785.034	Durbin-Watson:	0.718
Prob(Omnibus):	0.000	Jarque-Bera (JB):	976.607
Skew:	-0.749	Prob(JB):	8.56e-213
Kurtosis:	3.262	Cond. No.	9.29

==

Task: cardPaiprocessed

OLS Regression Results

==

Dep. Variable:	value_z	R-squared:	0.061
Model:	OLS	Adj. R-squared:	0.061
Method:	Least Squares	F-statistic:	223.9
Date:	Tue, 09 Jan 2024	Prob (F-statistic):	1.10e-140
Time:	21:34:53	Log-Likelihood:	-14231.
No. Observations:	10259	AIC:	2.847e+04
Df Residuals:	10255	BIC:	2.850e+04
Df Model:	3		
Covariance Type:	nonrobust		

==

	coef	std err	t	P>|t|	[0.025	0.975]

```
-----------------------------------------------------------------------------
Intercept     0.2473     0.015    16.872     0.000      0.219      0.276
decade60s    -0.3375     0.021   -16.213     0.000     -0.378     -0.297
decade70s    -0.6409     0.029   -21.728     0.000     -0.699     -0.583
decade80s    -1.0702     0.079   -13.597     0.000     -1.225     -0.916
==============================================================================
===
Omnibus:                   50.543   Durbin-Watson:                 0.707
Prob(Omnibus):              0.000   Jarque-Bera (JB):             50.540
Skew:                      -0.162   Prob(JB):                   1.06e-11
Kurtosis:                   2.887   Cond. No.                       9.16
==============================================================================
===
```

Task: trailMaking

```
                     OLS Regression Results
==============================================================================
===
Dep. Variable:                value_z   R-squared:                      0.123
Model:                            OLS   Adj. R-squared:                 0.123
Method:                 Least Squares   F-statistic:                    523.9
Date:                Tue, 09 Jan 2024   Prob (F-statistic):         1.09e-318
Time:                        21:34:53   Log-Likelihood:                -15196.
No. Observations:               11228   AIC:                        3.040e+04
Df Residuals:                   11224   BIC:                        3.043e+04
Df Model:                           3
Covariance Type:            nonrobust
==============================================================================
===
                 coef    std err          t      P>|t|      [0.025      0.975]
-----------------------------------------------------------------------------
Intercept    -0.3346     0.013   -24.805     0.000     -0.361     -0.308
decade60s     0.4298     0.019    22.298     0.000      0.392      0.468
decade70s     0.9614     0.027    35.840     0.000      0.909      1.014
decade80s     1.3758     0.079    17.368     0.000      1.221      1.531
==============================================================================
===
Omnibus:                 1308.531   Durbin-Watson:                 0.694
Prob(Omnibus):              0.000   Jarque-Bera (JB):           1881.250
Skew:                       0.890   Prob(JB):                       0.00
Kurtosis:                   3.922   Cond. No.                       9.94
==============================================================================
===
```

Task: PAL

```
                     OLS Regression Results
```

```
==============================================================================
===
Dep. Variable:            value_z   R-squared:                   0.035
Model:                        OLS   Adj. R-squared:              0.034
Method:             Least Squares   F-statistic:                 67.38
Date:            Tue, 09 Jan 2024   Prob (F-statistic):       8.37e-43
Time:                    21:34:53   Log-Likelihood:            -7912.0
No. Observations:            5646   AIC:                     1.583e+04
Df Residuals:                5642   BIC:                     1.586e+04
Df Model:                       3
Covariance Type:        nonrobust
==============================================================================
===
              coef    std err          t      P>|t|      [0.025      0.975]
------------------------------------------------------------------------------
Intercept     0.1788      0.020      8.960      0.000       0.140       0.218
decade60s    -0.2409      0.029     -8.441      0.000      -0.297      -0.185
decade70s    -0.4575      0.040    -11.455      0.000      -0.536      -0.379
decade80s    -0.8903      0.105     -8.483      0.000      -1.096      -0.685
==============================================================================
===
Omnibus:                 1121.335   Durbin-Watson:               0.652
Prob(Omnibus):              0.000   Jarque-Bera (JB):         6566.161
Skew:                       0.821   Prob(JB):                     0.00
Kurtosis:                   8.021   Cond. No.                     8.91
==============================================================================
===

Task: pictureCompletion
                            OLS Regression Results
==============================================================================
===
Dep. Variable:            value_z   R-squared:                   0.021
Model:                        OLS   Adj. R-squared:              0.021
Method:             Least Squares   F-statistic:                 30.79
Date:            Tue, 09 Jan 2024   Prob (F-statistic):       1.11e-19
Time:                    21:34:54   Log-Likelihood:            -5943.6
No. Observations:            4221   AIC:                     1.190e+04
Df Residuals:                4217   BIC:                     1.192e+04
Df Model:                       3
Covariance Type:        nonrobust
==============================================================================
===
              coef    std err          t      P>|t|      [0.025      0.975]
------------------------------------------------------------------------------
Intercept    -0.0973      0.024     -4.068      0.000      -0.144      -0.050
decade60s     0.0720      0.033      2.178      0.029       0.007       0.137
```

decade70s 0.4266 0.048 8.878 0.000 0.332 0.521
decade80s 0.5190 0.123 4.211 0.000 0.277 0.761
==
===
Omnibus: 965.004 Durbin-Watson: 0.623
Prob(Omnibus): 0.000 Jarque-Bera (JB): 2567.760
Skew: 1.219 Prob(JB): 0.00
Kurtosis: 5.943 Cond. No. 9.10
==
===

Supplementary Model outputs including standard errors associated with each age decade per task for SECONDARY MEASURES

Task: target_correct_medianRT_recognitionMemory_immediate

OLS Regression Results
==
===
Dep. Variable: value_z R-squared: 0.042
Model: OLS Adj. R-squared: 0.042
Method: Least Squares F-statistic: 844.0
Date: Wed, 20 Dec 2023 Prob (F-statistic): 0.00
Time: 15:15:22 Log-Likelihood: -79720.
No. Observations: 57056 AIC: 1.594e+05
Df Residuals: 57052 BIC: 1.595e+05
Df Model: 3
Covariance Type: nonrobust
==
===
coef std err t P>|t| [0.025 0.975]

Intercept -0.1929 0.006 -32.695 0.000 -0.204 -0.181
decade60s 0.3180 0.009 36.479 0.000 0.301 0.335
decade70s 0.5294 0.014 37.160 0.000 0.501 0.557
decade80s 1.0059 0.041 24.767 0.000 0.926 1.086
==
===
Omnibus: 37177.859 Durbin-Watson: 1.169
Prob(Omnibus): 0.000 Jarque-Bera (JB): 1827680.903
Skew: 2.517 Prob(JB): 0.00
Kurtosis: 30.266 Cond. No. 10.9
==
===

Task: target_correct_medianRT_recognitionMemory_delayed

```
                    OLS Regression Results
==============================================================================
===
Dep. Variable:           value_z   R-squared:                 0.039
Model:                       OLS   Adj. R-squared:            0.039
Method:            Least Squares   F-statistic:               750.4
Date:           Wed, 20 Dec 2023   Prob (F-statistic):         0.00
Time:                   15:15:22   Log-Likelihood:           -77060.
No. Observations:          55086   AIC:                   1.541e+05
Df Residuals:              55082   BIC:                   1.542e+05
Df Model:                      3
Covariance Type:       nonrobust
==============================================================================
===
             coef    std err       t     P>|t|     [0.025     0.975]
------------------------------------------------------------------------------
Intercept   -0.1893    0.006   -31.610    0.000     -0.201     -0.178
decade60s    0.3217    0.009    36.261    0.000      0.304      0.339
decade70s    0.5137    0.015    34.922    0.000      0.485      0.542
decade80s    0.8563    0.042    20.495    0.000      0.774      0.938
==============================================================================
===
Omnibus:               36907.633   Durbin-Watson:             1.207
Prob(Omnibus):             0.000   Jarque-Bera (JB):    1499433.743
Skew:                      2.693   Prob(JB):                   0.00
Kurtosis:                 27.985   Cond. No.                   11.0
==============================================================================
===
```

Task: meanRT_targetDetection

```
                    OLS Regression Results
==============================================================================
===
Dep. Variable:           value_z   R-squared:                 0.050
Model:                       OLS   Adj. R-squared:            0.050
Method:            Least Squares   F-statistic:               4087.
Date:           Wed, 20 Dec 2023   Prob (F-statistic):         0.00
Time:                   15:15:22   Log-Likelihood:       -3.2796e+05
No. Observations:         235340   AIC:                   6.559e+05
Df Residuals:             235336   BIC:                   6.560e+05
Df Model:                      3
Covariance Type:       nonrobust
==============================================================================
===
             coef    std err       t     P>|t|     [0.025     0.975]
------------------------------------------------------------------------------
Intercept   -0.2105    0.003   -73.347    0.000     -0.216     -0.205
```

```
decade60s     0.3529     0.004    81.883     0.000      0.344      0.361
decade70s     0.5835     0.007    86.370     0.000      0.570      0.597
decade80s     0.8730     0.019    45.154     0.000      0.835      0.911
==============================================================================
===
Omnibus:                 131173.024   Durbin-Watson:                 1.312
Prob(Omnibus):                0.000   Jarque-Bera (JB):       26491590.425
Skew:                         1.588   Prob(JB):                       0.00
Kurtosis:                    54.880   Cond. No.                       10.5
==============================================================================
===
```

Task: medianRT_emotionDiscrim

```
                            OLS Regression Results
==============================================================================
===
Dep. Variable:                value_z   R-squared:                       0.035
Model:                            OLS   Adj. R-squared:                  0.035
Method:                 Least Squares   F-statistic:                     1920.
Date:                Wed, 20 Dec 2023   Prob (F-statistic):               0.00
Time:                        15:15:22   Log-Likelihood:            -2.1995e+05
No. Observations:              157000   AIC:                         4.399e+05
Df Residuals:                  156996   BIC:                         4.399e+05
Df Model:                           3
Covariance Type:            nonrobust
==============================================================================
===
                 coef    std err          t      P>|t|      [0.025      0.975]
------------------------------------------------------------------------------
Intercept     -0.1585      0.003    -46.248      0.000      -0.165      -0.152
decade60s      0.2729      0.005     51.566      0.000       0.263       0.283
decade70s      0.5207      0.009     58.295      0.000       0.503       0.538
decade80s      0.9395      0.026     35.539      0.000       0.888       0.991
==============================================================================
===
Omnibus:                 143236.177   Durbin-Watson:                 1.664
Prob(Omnibus):                0.000   Jarque-Bera (JB):       23088698.366
Skew:                         3.867   Prob(JB):                       0.00
Kurtosis:                    61.904   Cond. No.                       11.6
==============================================================================
===
```

Task: medRT_manipulations2D

```
                            OLS Regression Results
==============================================================================
===
Dep. Variable:                value_z   R-squared:                       0.045
```

```
Model:                    OLS   Adj. R-squared:              0.045
Method:             Least Squares   F-statistic:                3718.
Date:             Wed, 20 Dec 2023   Prob (F-statistic):           0.00
Time:                  15:15:22   Log-Likelihood:         -3.2736e+05
No. Observations:           234549   AIC:                  6.547e+05
Df Residuals:              234545   BIC:                6.548e+05
Df Model:                    3
Covariance Type:          nonrobust
==============================================================================
===
            coef    std err        t     P>|t|     [0.025     0.975]
------------------------------------------------------------------------------
Intercept    -0.1898     0.003   -65.874     0.000     -0.195     -0.184
decade60s     0.2977     0.004    68.799     0.000      0.289      0.306
decade70s     0.5830     0.007    85.959     0.000      0.570      0.596
decade80s     0.9563     0.019    49.271     0.000      0.918      0.994
==============================================================================
===
Omnibus:              122773.253   Durbin-Watson:               1.280
Prob(Omnibus):              0.000   Jarque-Bera (JB):       2569083.828
Skew:                    2.063   Prob(JB):                   0.00
Kurtosis:                18.680   Cond. No.                   10.5
==============================================================================
===
```

Task: medRT_spatialSpan

```
                    OLS Regression Results
==============================================================================
===
Dep. Variable:            value_z   R-squared:                  0.009
Model:                    OLS   Adj. R-squared:              0.009
Method:             Least Squares   F-statistic:                699.4
Date:             Wed, 20 Dec 2023   Prob (F-statistic):           0.00
Time:                  15:15:23   Log-Likelihood:         -3.3444e+05
No. Observations:           236431   AIC:                  6.689e+05
Df Residuals:              236427   BIC:                6.689e+05
Df Model:                    3
Covariance Type:          nonrobust
==============================================================================
===
            coef    std err        t     P>|t|     [0.025     0.975]
------------------------------------------------------------------------------
Intercept    -0.0878     0.003   -30.031     0.000     -0.094     -0.082
decade60s     0.1485     0.004    33.809     0.000      0.140      0.157
decade70s     0.2309     0.007    33.571     0.000      0.217      0.244
decade80s     0.4399     0.020    22.294     0.000      0.401      0.479
```

==
===
Omnibus: 215639.525 Durbin-Watson: 1.243
Prob(Omnibus): 0.000 Jarque-Bera (JB): 40997413.852
Skew: 3.809 Prob(JB): 0.00
Kurtosis: 67.059 Cond. No. 10.6
==
===

Task: medRT_blocks

OLS Regression Results

==
===

Dep. Variable:	value_z	R-squared:	0.038
Model:	OLS	Adj. R-squared:	0.038
Method:	Least Squares	F-statistic:	3171.
Date:	Wed, 20 Dec 2023	Prob (F-statistic):	0.00
Time:	15:15:23	Log-Likelihood:	-3.3704e+05
No. Observations:	240820	AIC:	6.741e+05
Df Residuals:	240816	BIC:	6.741e+05
Df Model:	3		
Covariance Type:	nonrobust		

==
===

	coef	std err	t	P>|t|	[0.025	0.975]
Intercept	-0.1799	0.003	-62.886	0.000	-0.186	-0.174
decade60s	0.2908	0.004	67.786	0.000	0.282	0.299
decade70s	0.5181	0.007	77.502	0.000	0.505	0.531
decade80s	0.8363	0.019	44.056	0.000	0.799	0.873

==
===

Omnibus:	121863.800	Durbin-Watson:	1.304
Prob(Omnibus):	0.000	Jarque-Bera (JB):	1278988.034
Skew:	2.207	Prob(JB):	0.00
Kurtosis:	13.391	Cond. No.	10.4

==
===

Task: medRT_verbalAnalogies

OLS Regression Results

==
===

Dep. Variable:	value_z	R-squared:	0.041
Model:	OLS	Adj. R-squared:	0.041
Method:	Least Squares	F-statistic:	3412.
Date:	Wed, 20 Dec 2023	Prob (F-statistic):	0.00

```
Time:                    15:15:23   Log-Likelihood:          -3.3053e+05
No. Observations:           236471   AIC:                     6.611e+05
Df Residuals:               236467   BIC:                     6.611e+05
Df Model:                        3
Covariance Type:         nonrobust
==============================================================================
===
                 coef    std err          t      P>|t|      [0.025      0.975]
------------------------------------------------------------------------------
Intercept     -0.1645      0.003    -57.186      0.000      -0.170      -0.159
decade60s      0.2357      0.004     54.582      0.000       0.227       0.244
decade70s      0.5557      0.007     82.211      0.000       0.542       0.569
decade80s      1.0963      0.019     56.827      0.000       1.058       1.134
==============================================================================
===
Omnibus:                104796.199   Durbin-Watson:                   1.274
Prob(Omnibus):               0.000   Jarque-Bera (JB):          2087512.245
Skew:                        1.653   Prob(JB):                         0.00
Kurtosis:                   17.175   Cond. No.                         10.5
==============================================================================
===
```

Task: medRT_median_wordDefinitions

```
                            OLS Regression Results
==============================================================================
===
Dep. Variable:                value_z   R-squared:                       0.002
Model:                            OLS   Adj. R-squared:                  0.002
Method:                 Least Squares   F-statistic:                     74.50
Date:                Wed, 20 Dec 2023   Prob (F-statistic):           3.92e-48
Time:                        15:15:23   Log-Likelihood:            -1.5630e+05
No. Observations:              110234   AIC:                         3.126e+05
Df Residuals:                  110230   BIC:                         3.127e+05
Df Model:                           3
Covariance Type:            nonrobust
==============================================================================
===
                 coef    std err          t      P>|t|      [0.025      0.975]
------------------------------------------------------------------------------
Intercept      0.0461      0.004     10.358      0.000       0.037       0.055
decade60s     -0.0873      0.006    -13.482      0.000      -0.100      -0.075
decade70s     -0.0908      0.010     -9.329      0.000      -0.110      -0.072
decade80s      0.0502      0.027      1.840      0.066      -0.003       0.104
==============================================================================
===
Omnibus:                 37042.942   Durbin-Watson:                   0.969
Prob(Omnibus):               0.000   Jarque-Bera (JB):           150313.340
```

Skew: 1.634 Prob(JB): 0.00
Kurtosis: 7.695 Cond. No. 10.0
==
===

Task: medRT_digitSpan

OLS Regression Results
==
===
Dep. Variable: value_z R-squared: 0.023
Model: OLS Adj. R-squared: 0.023
Method: Least Squares F-statistic: 1818.
Date: Wed, 20 Dec 2023 Prob (F-statistic): 0.00
Time: 15:15:24 Log-Likelihood: -3.3337e+05
No. Observations: 236845 AIC: 6.668e+05
Df Residuals: 236841 BIC: 6.668e+05
Df Model: 3
Covariance Type: nonrobust
==
===

	coef	std err	t	P>|t|	[0.025	0.975]
Intercept	-0.1357	0.003	-46.796	0.000	-0.141	-0.130
decade60s	0.2182	0.004	50.096	0.000	0.210	0.227
decade70s	0.3978	0.007	58.226	0.000	0.384	0.411
decade80s	0.6960	0.020	35.318	0.000	0.657	0.735

==
===
Omnibus: 206232.116 Durbin-Watson: 1.173
Prob(Omnibus): 0.000 Jarque-Bera (JB): 27083702.189
Skew: 3.633 Prob(JB): 0.00
Kurtosis: 54.881 Cond. No. 10.6
==
===

Task: medianRT_TOL

OLS Regression Results
==
===
Dep. Variable: value_z R-squared: 0.018
Model: OLS Adj. R-squared: 0.018
Method: Least Squares F-statistic: 1442.
Date: Wed, 20 Dec 2023 Prob (F-statistic): 0.00
Time: 15:15:24 Log-Likelihood: -3.3715e+05
No. Observations: 239116 AIC: 6.743e+05
Df Residuals: 239112 BIC: 6.743e+05
Df Model: 3

```
Covariance Type:          nonrobust
==============================================================================
===
            coef    std err        t     P>|t|     [0.025     0.975]
------------------------------------------------------------------------------
Intercept    -0.1165     0.003    -40.186     0.000     -0.122     -0.111
decade60s     0.1805     0.004     41.512     0.000      0.172      0.189
decade70s     0.3522     0.007     51.859     0.000      0.339      0.366
decade80s     0.6719     0.019     34.791     0.000      0.634      0.710
==============================================================================
===
Omnibus:                108713.913   Durbin-Watson:               1.325
Prob(Omnibus):               0.000   Jarque-Bera (JB):      1261799.747
Skew:                        1.886   Prob(JB):                     0.00
Kurtosis:                   13.602   Cond. No.                     10.4
==============================================================================
===
```

Task: medRT_DRI_fourToweprocessed

```
                    OLS Regression Results
==============================================================================
===
Dep. Variable:             value_z   R-squared:                   0.039
Model:                         OLS   Adj. R-squared:              0.039
Method:              Least Squares   F-statistic:                 1054.
Date:             Wed, 20 Dec 2023   Prob (F-statistic):           0.00
Time:                     15:15:24   Log-Likelihood:        -1.0920e+05
No. Observations:            78049   AIC:                    2.184e+05
Df Residuals:                78045   BIC:                    2.184e+05
Df Model:                        3
Covariance Type:         nonrobust
==============================================================================
===
            coef    std err        t     P>|t|     [0.025     0.975]
------------------------------------------------------------------------------
Intercept    -0.1950     0.005    -37.957     0.000     -0.205     -0.185
decade60s     0.3101     0.008     41.250     0.000      0.295      0.325
decade70s     0.5301     0.012     45.547     0.000      0.507      0.553
decade80s     0.7079     0.033     21.687     0.000      0.644      0.772
==============================================================================
===
Omnibus:                 24790.323   Durbin-Watson:               1.048
Prob(Omnibus):               0.000   Jarque-Bera (JB):       133305.565
Skew:                        1.435   Prob(JB):                     0.00
Kurtosis:                    8.724   Cond. No.                     10.3
==============================================================================
===
```

Task: medRT_switchingStroop

```
                            OLS Regression Results
===========================================================================
===
Dep. Variable:                value_z   R-squared:                       0.144
Model:                            OLS   Adj. R-squared:                  0.143
Method:                 Least Squares   F-statistic:                     567.3
Date:                Wed, 20 Dec 2023   Prob (F-statistic):               0.00
Time:                        15:15:24   Log-Likelihood:                -13605.
No. Observations:               10143   AIC:                         2.722e+04
Df Residuals:                   10139   BIC:                         2.725e+04
Df Model:                           3
Covariance Type:            nonrobust
===========================================================================
===
                 coef    std err          t      P>|t|      [0.025      0.975]
------------------------------------------------------------------------------
Intercept     -0.3782      0.014    -26.813      0.000      -0.406      -0.351
decade60s      0.5194      0.020     25.995      0.000       0.480       0.559
decade70s      0.9583      0.028     33.745      0.000       0.903       1.014
decade80s      1.7454      0.077     22.777      0.000       1.595       1.896
===========================================================================
===
Omnibus:                     1906.158   Durbin-Watson:                   0.710
Prob(Omnibus):                  0.000   Jarque-Bera (JB):             4384.727
Skew:                           1.070   Prob(JB):                         0.00
Kurtosis:                       5.407   Cond. No.                         9.29
===========================================================================
===
```

Task: meanIncongAcc_switchingStroop

```
                            OLS Regression Results
===========================================================================
===
Dep. Variable:                value_z   R-squared:                       0.068
Model:                            OLS   Adj. R-squared:                  0.067
Method:                 Least Squares   F-statistic:                     245.5
Date:                Wed, 20 Dec 2023   Prob (F-statistic):          8.57e-154
Time:                        15:15:24   Log-Likelihood:                -14037.
No. Observations:               10143   AIC:                         2.808e+04
Df Residuals:                   10139   BIC:                         2.811e+04
Df Model:                           3
Covariance Type:            nonrobust
===========================================================================
===
                 coef    std err          t      P>|t|      [0.025      0.975]
```

```
------------------------------------------------------------------------------
Intercept     0.2760     0.015    18.751     0.000     0.247     0.305
decade60s    -0.4023     0.021   -19.296     0.000    -0.443    -0.361
decade70s    -0.6523     0.030   -22.014     0.000    -0.710    -0.594
decade80s    -1.0525     0.080   -13.163     0.000    -1.209    -0.896
==============================================================================
===
Omnibus:                  558.924   Durbin-Watson:                 0.708
Prob(Omnibus):              0.000   Jarque-Bera (JB):            597.538
Skew:                      -0.565   Prob(JB):                 1.76e-130
Kurtosis:                   2.631   Cond. No.                       9.29
==============================================================================
===
```

Task: medIncongRT_switchingStroop

```
                  OLS Regression Results
==============================================================================
===
Dep. Variable:              value_z   R-squared:                     0.152
Model:                          OLS   Adj. R-squared:                0.152
Method:               Least Squares   F-statistic:                   605.5
Date:              Wed, 20 Dec 2023   Prob (F-statistic):             0.00
Time:                      15:15:24   Log-Likelihood:               -13551.
No. Observations:             10139   AIC:                       2.711e+04
Df Residuals:                 10135   BIC:                       2.714e+04
Df Model:                         3
Covariance Type:          nonrobust
==============================================================================
===
              coef    std err          t      P>|t|      [0.025      0.975]
------------------------------------------------------------------------------
Intercept    -0.3839     0.014   -27.345     0.000    -0.411    -0.356
decade60s     0.5213     0.020    26.209     0.000     0.482     0.560
decade70s     0.9840     0.028    34.816     0.000     0.929     1.039
decade80s     1.8354     0.076    24.069     0.000     1.686     1.985
==============================================================================
===
Omnibus:                 2169.846   Durbin-Watson:                 0.695
Prob(Omnibus):              0.000   Jarque-Bera (JB):           5446.600
Skew:                       1.173   Prob(JB):                       0.00
Kurtosis:                   5.718   Cond. No.                       9.29
==============================================================================
===
```

Task: meanSwitchAcc_switchingStroop

```
                  OLS Regression Results
```

==
Dep. Variable: value_z R-squared: 0.041
Model: OLS Adj. R-squared: 0.040
Method: Least Squares F-statistic: 143.0
Date: Wed, 20 Dec 2023 Prob (F-statistic): 9.76e-91
Time: 15:15:25 Log-Likelihood: -14182.
No. Observations: 10143 AIC: 2.837e+04
Df Residuals: 10139 BIC: 2.840e+04
Df Model: 3
Covariance Type: nonrobust
==

	coef	std err	t	P>|t|	[0.025	0.975]
Intercept	0.2189	0.015	14.657	0.000	0.190	0.248
decade60s	-0.3355	0.021	-15.863	0.000	-0.377	-0.294
decade70s	-0.4644	0.030	-15.448	0.000	-0.523	-0.405
decade80s	-0.8609	0.081	-10.614	0.000	-1.020	-0.702

==
Omnibus: 1397.407 Durbin-Watson: 0.724
Prob(Omnibus): 0.000 Jarque-Bera (JB): 2061.224
Skew: -1.027 Prob(JB): 0.00
Kurtosis: 3.812 Cond. No. 9.29
==

Task: medSwitchRT_switchingStroop

OLS Regression Results
==
Dep. Variable: value_z R-squared: 0.100
Model: OLS Adj. R-squared: 0.100
Method: Least Squares F-statistic: 375.4
Date: Wed, 20 Dec 2023 Prob (F-statistic): 3.25e-231
Time: 15:15:25 Log-Likelihood: -13858.
No. Observations: 10143 AIC: 2.772e+04
Df Residuals: 10139 BIC: 2.775e+04
Df Model: 3
Covariance Type: nonrobust
==

	coef	std err	t	P>|t|	[0.025	0.975]
Intercept	-0.3100	0.014	-21.435	0.000	-0.338	-0.282
decade60s	0.4275	0.020	20.868	0.000	0.387	0.468

```
decade70s     0.7611     0.029    26.140     0.000      0.704      0.818
decade80s     1.6079     0.079    20.466     0.000      1.454      1.762
==============================================================================
===
Omnibus:                  2265.842   Durbin-Watson:                 0.705
Prob(Omnibus):               0.000   Jarque-Bera (JB):           6279.355
Skew:                        1.184   Prob(JB):                       0.00
Kurtosis:                    6.042   Cond. No.                       9.29
==============================================================================
===
```

Task: totalTimeTaken_pictureCompletion

```
                            OLS Regression Results
==============================================================================
===
Dep. Variable:                value_z   R-squared:                       0.001
Model:                            OLS   Adj. R-squared:                 -0.000
Method:                 Least Squares   F-statistic:                    0.8076
Date:                Wed, 20 Dec 2023   Prob (F-statistic):              0.489
Time:                        15:15:25   Log-Likelihood:                -5988.1
No. Observations:                4221   AIC:                         1.198e+04
Df Residuals:                    4217   BIC:                         1.201e+04
Df Model:                           3
Covariance Type:            nonrobust
==============================================================================
===
                 coef    std err          t      P>|t|      [0.025      0.975]
------------------------------------------------------------------------------
Intercept      0.0276      0.024      1.141      0.254      -0.020      0.075
decade60s     -0.0512      0.033     -1.533      0.125      -0.117      0.014
decade70s     -0.0354      0.049     -0.729      0.466      -0.131      0.060
decade80s     -0.0040      0.125     -0.032      0.974      -0.248      0.240
==============================================================================
===
Omnibus:                 11266.859   Durbin-Watson:                 0.503
Prob(Omnibus):               0.000   Jarque-Bera (JB):      187422965.888
Skew:                       31.996   Prob(JB):                       0.00
Kurtosis:                 1033.323   Cond. No.                       9.10
==============================================================================
===
```

Task: medArrayTime_cardPaiprocessed

```
                            OLS Regression Results
==============================================================================
===
Dep. Variable:                value_z   R-squared:                       0.112
Model:                            OLS   Adj. R-squared:                  0.112
```

```
Method:              Least Squares   F-statistic:                 431.1
Date:             Wed, 20 Dec 2023   Prob (F-statistic):      8.95e-264
Time:                     15:15:25   Log-Likelihood:             -13948.
No. Observations:            10259   AIC:                     2.790e+04
Df Residuals:                10255   BIC:                     2.793e+04
Df Model:                        3
Covariance Type:         nonrobust
==============================================================================
===
              coef    std err          t      P>|t|      [0.025      0.975]
------------------------------------------------------------------------------
Intercept    -0.3317      0.014    -23.267      0.000      -0.360      -0.304
decade60s     0.4472      0.020     22.089      0.000       0.408       0.487
decade70s     0.8793      0.029     30.648      0.000       0.823       0.936
decade80s     1.4050      0.077     18.351      0.000       1.255       1.555
==============================================================================
===
Omnibus:                     2353.948   Durbin-Watson:                   0.682
Prob(Omnibus):                  0.000   Jarque-Bera (JB):             6336.699
Skew:                           1.227   Prob(JB):                         0.00
Kurtosis:                       5.967   Cond. No.                         9.16
==============================================================================
===
```

Task: medRT_PAL

```
                            OLS Regression Results
==============================================================================
===
Dep. Variable:                value_z   R-squared:                       0.048
Model:                            OLS   Adj. R-squared:                  0.048
Method:                 Least Squares   F-statistic:                     95.58
Date:                Wed, 20 Dec 2023   Prob (F-statistic):           2.45e-60
Time:                        15:15:25   Log-Likelihood:                -7871.4
No. Observations:                5646   AIC:                         1.575e+04
Df Residuals:                    5642   BIC:                         1.578e+04
Df Model:                           3
Covariance Type:            nonrobust
==============================================================================
===
              coef    std err          t      P>|t|      [0.025      0.975]
------------------------------------------------------------------------------
Intercept    -0.1934      0.020     -9.761      0.000      -0.232      -0.155
decade60s     0.2251      0.028      7.944      0.000       0.170       0.281
decade70s     0.6074      0.040     15.317      0.000       0.530       0.685
decade80s     0.8699      0.104      8.348      0.000       0.666       1.074
==============================================================================
===
```

```
Omnibus:                2798.491   Durbin-Watson:               0.670
Prob(Omnibus):              0.000   Jarque-Bera (JB):          34788.874
Skew:                   2.064   Prob(JB):                   0.00
Kurtosis:               14.439   Cond. No.                    8.91
==============================================================================
===
```

Task: medianRT_SRT

```
                   OLS Regression Results
==============================================================================
===
Dep. Variable:              value_z   R-squared:                   0.033
Model:                          OLS   Adj. R-squared:              0.032
Method:               Least Squares   F-statistic:                 67.80
Date:              Wed, 20 Dec 2023   Prob (F-statistic):          4.05e-43
Time:                      15:15:25   Log-Likelihood:              -8478.8
No. Observations:              6046   AIC:                    1.697e+04
Df Residuals:                  6042   BIC:                    1.699e+04
Df Model:                         3
Covariance Type:          nonrobust
==============================================================================
===
             coef    std err        t      P>|t|      [0.025      0.975]
------------------------------------------------------------------------------
Intercept    -0.1745     0.019    -9.172     0.000     -0.212     -0.137
decade60s     0.2492     0.027     9.085     0.000      0.195      0.303
decade70s     0.4586     0.039    11.727     0.000      0.382      0.535
decade80s     0.9285     0.127     7.288     0.000      0.679      1.178
==============================================================================
===
Omnibus:                2052.972   Durbin-Watson:               0.750
Prob(Omnibus):              0.000   Jarque-Bera (JB):           9798.221
Skew:                   1.576   Prob(JB):                   0.00
Kurtosis:                8.381   Cond. No.                    11.1
==============================================================================
===
```

Task: meanNumberRT_trailMaking

```
                   OLS Regression Results
==============================================================================
===
Dep. Variable:              value_z   R-squared:                   0.106
Model:                          OLS   Adj. R-squared:              0.106
Method:               Least Squares   F-statistic:                 443.2
Date:              Wed, 20 Dec 2023   Prob (F-statistic):          3.90e-272
Time:                      15:15:26   Log-Likelihood:              -15303.
No. Observations:             11228   AIC:                    3.061e+04
```

```
Df Residuals:              11224   BIC:                 3.064e+04
Df Model:                      3
Covariance Type:           nonrobust
==============================================================================
===
              coef    std err          t      P>|t|      [0.025      0.975]
------------------------------------------------------------------------------
Intercept    -0.2921      0.014    -21.450      0.000      -0.319      -0.265
decade60s     0.3505      0.019     18.014      0.000       0.312       0.389
decade70s     0.8931      0.027     32.977      0.000       0.840       0.946
decade80s     1.3860      0.080     17.330      0.000       1.229       1.543
==============================================================================
===
Omnibus:                 4106.843   Durbin-Watson:                  0.702
Prob(Omnibus):              0.000   Jarque-Bera (JB):           22387.657
Skew:                       1.672   Prob(JB):                        0.00
Kurtosis:                   9.056   Cond. No.                        9.94
==============================================================================
===
```

Task: meanLetterRT_trailMaking

```
                            OLS Regression Results
==============================================================================
===
Dep. Variable:                value_z   R-squared:                       0.090
Model:                            OLS   Adj. R-squared:                  0.090
Method:                 Least Squares   F-statistic:                     372.1
Date:                Wed, 20 Dec 2023   Prob (F-statistic):          2.15e-230
Time:                        15:15:26   Log-Likelihood:                -15400.
No. Observations:               11228   AIC:                         3.081e+04
Df Residuals:                   11224   BIC:                         3.084e+04
Df Model:                           3
Covariance Type:            nonrobust
==============================================================================
===
              coef    std err          t      P>|t|      [0.025      0.975]
------------------------------------------------------------------------------
Intercept    -0.2973      0.014    -21.646      0.000      -0.324      -0.270
decade60s     0.3970      0.020     20.229      0.000       0.359       0.435
decade70s     0.8214      0.027     30.071      0.000       0.768       0.875
decade80s     1.1094      0.081     13.753      0.000       0.951       1.268
==============================================================================
===
Omnibus:                 2316.655   Durbin-Watson:                  0.695
Prob(Omnibus):              0.000   Jarque-Bera (JB):            5055.977
Skew:                       1.193   Prob(JB):                        0.00
Kurtosis:                   5.262   Cond. No.                        9.94
```

==
===

Task: nlCost_trailMaking

```
                            OLS Regression Results
==============================================================================
===
Dep. Variable:                value_z   R-squared:                       0.016
Model:                            OLS   Adj. R-squared:                  0.016
Method:                 Least Squares   F-statistic:                     60.45
Date:                Wed, 20 Dec 2023   Prob (F-statistic):           9.20e-39
Time:                        15:15:26   Log-Likelihood:                -15842.
No. Observations:               11228   AIC:                         3.169e+04
Df Residuals:                   11224   BIC:                         3.172e+04
Df Model:                           3
Covariance Type:            nonrobust
==============================================================================
===
                 coef    std err          t      P>|t|      [0.025      0.975]
------------------------------------------------------------------------------
Intercept     -0.1392      0.014     -9.742      0.000      -0.167      -0.111
decade60s      0.2143      0.020     10.498      0.000       0.174       0.254
decade70s      0.3227      0.028     11.356      0.000       0.267       0.378
decade80s      0.3065      0.084      3.652      0.000       0.142       0.471
==============================================================================
===
Omnibus:                     2877.716   Durbin-Watson:                   0.698
Prob(Omnibus):                  0.000   Jarque-Bera (JB):            14342.386
Skew:                           1.151   Prob(JB):                         0.00
Kurtosis:                       8.036   Cond. No.                         9.94
==============================================================================
===
```

Task: meanRT_motorControl

```
                            OLS Regression Results
==============================================================================
===
Dep. Variable:                value_z   R-squared:                       0.029
Model:                            OLS   Adj. R-squared:                  0.029
Method:                 Least Squares   F-statistic:                     75.77
Date:                Wed, 20 Dec 2023   Prob (F-statistic):           2.79e-48
Time:                        15:15:26   Log-Likelihood:                -10577.
No. Observations:                7533   AIC:                         2.116e+04
Df Residuals:                    7529   BIC:                         2.119e+04
Df Model:                           3
Covariance Type:            nonrobust
```

```
===========================================================================
===
             coef    std err          t      P>|t|      [0.025      0.975]
------------------------------------------------------------------------------
Intercept    -0.1446      0.015     -9.365      0.000      -0.175      -0.114
decade60s     0.2583      0.025     10.441      0.000       0.210       0.307
decade70s     0.4732      0.038     12.377      0.000       0.398       0.548
decade80s     0.6405      0.122      5.238      0.000       0.401       0.880
===========================================================================
===
Omnibus:                     1244.885   Durbin-Watson:                   1.750
Prob(Omnibus):                  0.000   Jarque-Bera (JB):             2098.005
Skew:                           1.093   Prob(JB):                         0.00
Kurtosis:                       4.380   Cond. No.                         11.6
===========================================================================
===
```

3. Divergent outcomes in patients with PD&pRBD (probable RBD) relative to PD only

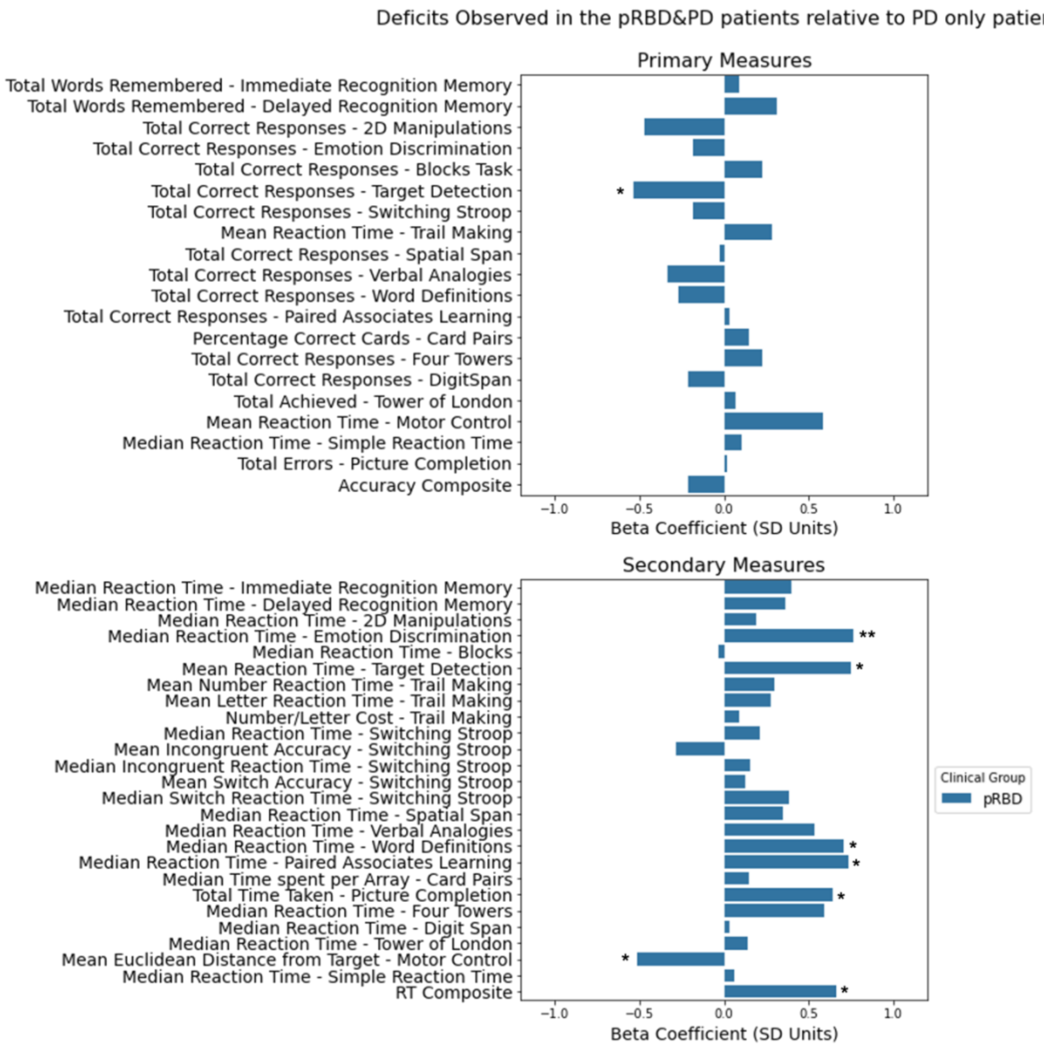

Supplementary Figure 7. Effect size differences in primary and secondary measures of task performance in patients with PD and pRBD relative to patients with PD only

index	params	task
pRBD	0.091	Total Words Remembered - Immediate Recognition Memory
pRBD	0.309	Total Words Remembered - Delayed Recognition Memory
pRBD	-0.470	Total Correct Responses - 2D Manipulations
pRBD	-0.185	Total Correct Responses - Emotion Discrimination
pRBD	0.226	Total Correct Responses - Blocks Task
pRBD	-0.534	Total Correct Responses - Target Detection
pRBD	-0.187	Total Correct Responses - Switching Stroop
pRBD	0.284	Mean Reaction Time - Trail Making
pRBD	-0.030	Total Correct Responses - Spatial Span
pRBD	-0.334	Total Correct Responses - Verbal Analogies
pRBD	-0.271	Total Correct Responses - Word Definitions

pRBD	0.034	Total Correct Responses - Paired Associates Learning
pRBD	0.143	Percentage Correct Cards - Card Pairs
pRBD	0.221	Total Correct Responses - Four Towers
pRBD	-0.212	Total Correct Responses - DigitSpan
pRBD	0.067	Total Achieved - Tower of London
pRBD	0.585	Mean Reaction Time - Motor Control
pRBD	0.100	Median Reaction Time - Simple Reaction Time
pRBD	0.013	Total Errors - Picture Completion
pRBD	-0.215	Accuracy Composite
pRBD	0.399	Median Reaction Time - Immediate Recognition Memory
pRBD	0.364	Median Reaction Time - Delayed Recognition Memory
pRBD	0.189	Median Reaction Time - 2D Manipulations
pRBD	0.765	Median Reaction Time - Emotion Discrimination
pRBD	-0.031	Median Reaction Time - Blocks
pRBD	0.747	Mean Reaction Time - Target Detection
pRBD	0.294	Mean Number Reaction Time - Trail Making
pRBD	0.277	Mean Letter Reaction Time - Trail Making
pRBD	0.088	Number/Letter Cost - Trail Making
pRBD	0.212	Median Reaction Time - Switching Stroop
pRBD	-0.284	Mean Incongruent Accuracy - Switching Stroop
pRBD	0.150	Median Incongruent Reaction Time - Switching Stroop
pRBD	0.124	Mean Switch Accuracy - Switching Stroop
pRBD	0.382	Median Switch Reaction Time - Switching Stroop
pRBD	0.345	Median Reaction Time - Spatial Span
pRBD	0.530	Median Reaction Time - Verbal Analogies
pRBD	0.706	Median Reaction Time - Word Definitions
pRBD	0.736	Median Reaction Time - Paired Associates Learning
pRBD	0.148	Median Time spent per Array - Card Pairs
pRBD	0.641	Total Time Taken - Picture Completion
pRBD	0.591	Median Reaction Time - Four Towers
pRBD	0.029	Median Reaction Time - Digit Span
pRBD	0.138	Median Reaction Time - Tower of London
pRBD	-0.515	Mean Euclidean Distance from Target - Motor Control
pRBD	0.056	Median Reaction Time - Simple Reaction Time
pRBD	0.661	RT Composite

Supplementary Table 15. Beta coefficients representing the effect size differences in patients with PD&pRBD relative to PD only patients

	sum_sq	df	F	PR(>F)	task
pRBD	0.091	1.000	0.103	0.749	Total Words Remembered - Immediate Recognition Memory
Residual	46.627	53.000			Total Words Remembered - Immediate Recognition Memory

pRBD	1.044	1.000	1.595	0.212	Total Words Remembered - Delayed Recognition Memory
Residual	34.687	53.000			Total Words Remembered - Delayed Recognition Memory
pRBD	2.415	1.000	2.274	0.137	Total Correct Responses - 2D Manipulations Task
Residual	56.274	53.000			Total Correct Responses - 2D Manipulations Task
pRBD	0.373	1.000	0.309	0.581	Total Correct Responses - Emotion Discrimination Task
Residual	64.117	53.000			Total Correct Responses - Emotion Discrimination Task
pRBD	0.560	1.000	0.481	0.491	Total Correct Responses - Blocks Task
Residual	61.611	53.000			Total Correct Responses - Blocks Task
pRBD	3.113	1.000	4.241	0.044	Total Correct Responses - Target Detection Task
Residual	38.895	53.000			Total Correct Responses - Target Detection Task
pRBD	0.377	1.000	0.455	0.503	Total Correct Responses - Switching Stroop Task
Residual	43.067	52.000			Total Correct Responses - Switching Stroop Task
pRBD	0.876	1.000	0.973	0.329	Total Correct Responses - Trail Making Task
Residual	46.836	52.000			Total Correct Responses - Trail Making Task
pRBD	0.010	1.000	0.011	0.916	Total Correct Responses - Spatial Span Task
Residual	46.342	53.000			Total Correct Responses - Spatial Span Task
pRBD	1.220	1.000	1.245	0.270	Total Correct Responses - Verbal Analogies Task
Residual	51.942	53.000			Total Correct Responses - Verbal Analogies Task
pRBD	0.804	1.000	1.096	0.300	Total Words Remembered - Word Definitions Task
Residual	38.861	53.000			Total Words Remembered - Word Definitions Task
pRBD	0.012	1.000	0.012	0.914	Total Correct Responses - Paired Associates Learning Task
Residual	54.577	52.000			Total Correct Responses - Paired Associates Learning Task
pRBD	0.220	1.000	0.180	0.674	Percentage Correct Cards - Card Pairs Task
Residual	63.876	52.000			Percentage Correct Cards - Card Pairs Task
pRBD	0.526	1.000	0.471	0.496	Total Achieved - Four Towers Task
Residual	56.954	51.000			Total Achieved - Four Towers Task
pRBD	0.491	1.000	0.501	0.482	Total Correct Responses - Digit Span Task
Residual	51.984	53.000			Total Correct Responses - Digit Span Task
pRBD	0.049	1.000	0.054	0.818	Total Achieved - Tower of London Task
Residual	48.483	53.000			Total Achieved - Tower of London Task
pRBD	3.739	1.000	3.545	0.065	Mean Reaction Time - Motor Control Task
Residual	55.890	53.000			Mean Reaction Time - Motor Control Task
pRBD	0.108	1.000	0.118	0.733	Median Reaction Time - Simple Reaction Time Task
Residual	47.948	52.000			Median Reaction Time - Simple Reaction Time Task
pRBD	0.002	1.000	0.002	0.962	Total Errors - Picture Completion Task
Residual	42.209	52.000			Total Errors - Picture Completion Task

**pRBD**	0.496	1.000	0.684	0.412	Accuracy Composite
**Residual**	37.032	51.000			Accuracy Composite
**pRBD**	1.738	1.000	1.615	0.209	Immediate Prospective Memory - Median Reaction Time
**Residual**	57.020	53.000			Immediate Prospective Memory - Median Reaction Time
**pRBD**	1.443	1.000	1.796	0.186	Delayed Prospective Memory - Median Reaction Time
**Residual**	42.587	53.000			Delayed Prospective Memory - Median Reaction Time
**pRBD**	0.388	1.000	0.496	0.484	Manipulations 2D - Median Reaction Time
**Residual**	41.506	53.000			Manipulations 2D - Median Reaction Time
**pRBD**	6.386	1.000	9.749	0.003	Emotion Discrimination - Median Reaction Time
**Residual**	34.718	53.000			Emotion Discrimination - Median Reaction Time
**pRBD**	0.011	1.000	0.014	0.908	Blocks - Median Reaction Time
**Residual**	42.359	53.000			Blocks - Median Reaction Time
**pRBD**	6.082	1.000	7.142	0.010	Target Detection - Mean Reaction Time
**Residual**	45.132	53.000			Target Detection - Mean Reaction Time
**pRBD**	0.937	1.000	0.899	0.347	Trail Making - Mean Number Reaction Time
**Residual**	54.234	52.000			Trail Making - Mean Number Reaction Time
**pRBD**	0.831	1.000	0.998	0.322	Trail Making - Mean Letter Reaction Time
**Residual**	43.288	52.000			Trail Making - Mean Letter Reaction Time
**pRBD**	0.083	1.000	0.098	0.756	Trail Making - Number/Letter Cost
**Residual**	44.449	52.000			Trail Making - Number/Letter Cost
**pRBD**	0.487	1.000	0.474	0.494	Switching Stroop - Median Reaction Time
**Residual**	53.442	52.000			Switching Stroop - Median Reaction Time
**pRBD**	0.874	1.000	1.130	0.293	Switching Stroop - Mean Incongruent Accuracy
**Residual**	40.230	52.000			Switching Stroop - Mean Incongruent Accuracy
**pRBD**	0.243	1.000	0.254	0.617	Switching Stroop - Median Incongruent Reaction Time
**Residual**	49.862	52.000			Switching Stroop - Median Incongruent Reaction Time
**pRBD**	0.165	1.000	0.215	0.644	Switching Stroop - Mean Switch Accuracy
**Residual**	39.938	52.000			Switching Stroop - Mean Switch Accuracy
**pRBD**	1.578	1.000	1.223	0.274	Switching Stroop - Median Switch Reaction Time
**Residual**	67.087	52.000			Switching Stroop - Median Switch Reaction Time
**pRBD**	1.298	1.000	1.600	0.211	Spatial Span - Median Reaction Time
**Residual**	42.980	53.000			Spatial Span - Median Reaction Time
**pRBD**	3.064	1.000	3.956	0.052	Verbal Analogies - Median Reaction Time
**Residual**	41.059	53.000			Verbal Analogies - Median Reaction Time
**pRBD**	5.434	1.000	6.560	0.013	Word Definitions - Median Reaction Time
**Residual**	43.900	53.000			Word Definitions - Median Reaction Time

**pRBD**	5.867	1.000	6.999	0.011	PAL - Median Reaction Time
**Residual**	43.587	52.000			PAL - Median Reaction Time
**pRBD**	0.238	1.000	0.222	0.639	Card Pairs - Median Array Time
**Residual**	55.575	52.000			Card Pairs - Median Array Time
**pRBD**	4.446	1.000	5.116	0.028	Picture Completion - Total Time Taken
**Residual**	45.186	52.000			Picture Completion - Total Time Taken
**pRBD**	3.763	1.000	4.658	0.036	Four Towers - Median Reaction Time
**Residual**	41.194	51.000			Four Towers - Median Reaction Time
**pRBD**	0.009	1.000	0.011	0.917	Digit Span - Median Reaction Time
**Residual**	43.564	53.000			Digit Span - Median Reaction Time
**pRBD**	0.206	1.000	0.243	0.624	Tower of London - Median Reaction Time
**Residual**	44.951	53.000			Tower of London - Median Reaction Time
**pRBD**	2.895	1.000	2.522	0.118	Motor Control - Mean Euclidean Distance from Target
**Residual**	60.853	53.000			Motor Control - Mean Euclidean Distance from Target
**pRBD**	0.034	1.000	0.038	0.847	Simple Reaction Time - Median Reaction Time
**Residual**	46.595	52.000			Simple Reaction Time - Median Reaction Time
**pRBD**	4.699	1.000	5.394	0.024	RT Composite
**Residual**	44.437	51.000			RT Composite

Supplementary **Table 16. ANOVA results looking at the significance of effect size differences in patients with PD&pRBD relative to PD only patients**

4. Divergent outcomes in patients with PD&RBD vs patients with idiopathic RBD

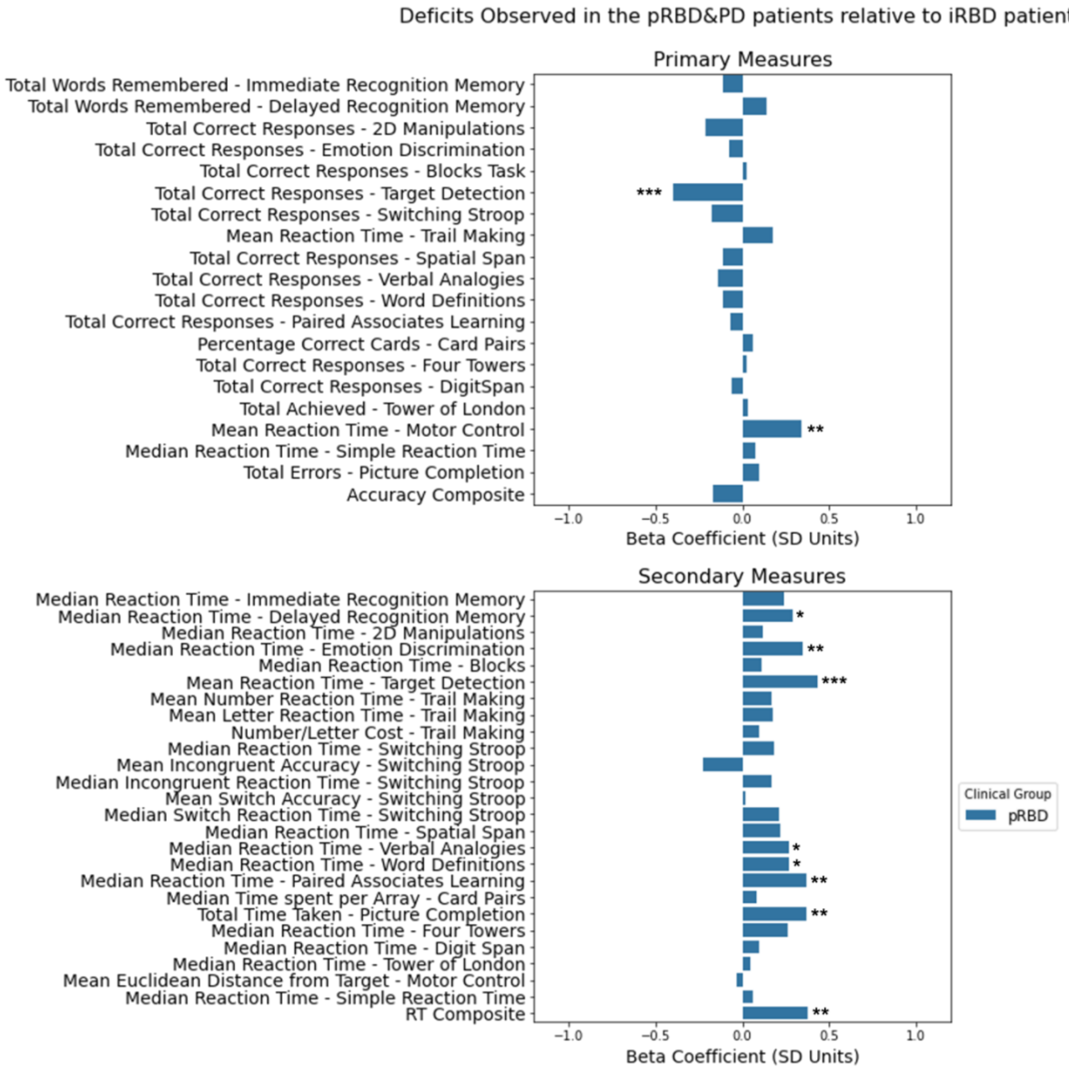

Supplementary Figure 8. Effect size differences in primary and secondary measures of task performance in patients with PD and pRBD relative to patients with iRBD

index	params	task
pRBD	-0.114	Total Words Remembered - Immediate Recognition Memory
pRBD	0.136	Total Words Remembered - Delayed Recognition Memory
pRBD	-0.212	Total Correct Responses - 2D Manipulations
pRBD	-0.075	Total Correct Responses - Emotion Discrimination
pRBD	0.022	Total Correct Responses - Blocks Task
pRBD	-0.399	Total Correct Responses - Target Detection
pRBD	-0.180	Total Correct Responses - Switching Stroop
pRBD	0.177	Mean Reaction Time - Trail Making
pRBD	-0.111	Total Correct Responses - Spatial Span
pRBD	-0.141	Total Correct Responses - Verbal Analogies
pRBD	-0.115	Total Correct Responses - Word Definitions

pRBD	-0.073	Total Correct Responses - Paired Associates Learning
pRBD	0.062	Percentage Correct Cards - Card Pairs
pRBD	0.020	Total Correct Responses - Four Towers
pRBD	-0.060	Total Correct Responses - DigitSpan
pRBD	0.028	Total Achieved - Tower of London
pRBD	0.342	Mean Reaction Time - Motor Control
pRBD	0.071	Median Reaction Time - Simple Reaction Time
pRBD	0.092	Total Errors - Picture Completion
pRBD	-0.173	Accuracy Composite
pRBD	0.237	Median Reaction Time - Immediate Recognition Memory
pRBD	0.290	Median Reaction Time - Delayed Recognition Memory
pRBD	0.120	Median Reaction Time - 2D Manipulations
pRBD	0.347	Median Reaction Time - Emotion Discrimination
pRBD	0.113	Median Reaction Time - Blocks
pRBD	0.435	Mean Reaction Time - Target Detection
pRBD	0.169	Mean Number Reaction Time - Trail Making
pRBD	0.176	Mean Letter Reaction Time - Trail Making
pRBD	0.094	Number/Letter Cost - Trail Making
pRBD	0.179	Median Reaction Time - Switching Stroop
pRBD	-0.227	Mean Incongruent Accuracy - Switching Stroop
pRBD	0.166	Median Incongruent Reaction Time - Switching Stroop
pRBD	0.019	Mean Switch Accuracy - Switching Stroop
pRBD	0.206	Median Switch Reaction Time - Switching Stroop
pRBD	0.219	Median Reaction Time - Spatial Span
pRBD	0.266	Median Reaction Time - Verbal Analogies
pRBD	0.267	Median Reaction Time - Word Definitions
pRBD	0.366	Median Reaction Time - Paired Associates Learning
pRBD	0.080	Median Time spent per Array - Card Pairs
pRBD	0.365	Total Time Taken - Picture Completion
pRBD	0.260	Median Reaction Time - Four Towers
pRBD	0.097	Median Reaction Time - Digit Span
pRBD	0.042	Median Reaction Time - Tower of London
pRBD	-0.034	Mean Euclidean Distance from Target - Motor Control
pRBD	0.060	Median Reaction Time - Simple Reaction Time
pRBD	0.374	RT Composite

Supplementary Table 17. Beta coefficients representing the effect size differences in patients with PD&pRBD relative to patients with iRBD

	sum_sq	df	F	PR(>F)	task
pRBD	0.779	1.000	1.177	0.296	Total Words Remembered - Immediate Recognition Memory
Residual	9.274	14.000			Total Words Remembered - Immediate Recognition Memory

**pRBD**	1.102	1.000	1.225	0.287	Total Words Remembered - Delayed Recognition Memory
**Residual**	12.600	14.000			Total Words Remembered - Delayed Recognition Memory
**pRBD**	2.687	1.000	4.433	0.054	Total Correct Responses - 2D Manipulations Task
**Residual**	8.484	14.000			Total Correct Responses - 2D Manipulations Task
**pRBD**	0.334	1.000	0.295	0.596	Total Correct Responses - Emotion Discrimination Task
**Residual**	15.868	14.000			Total Correct Responses - Emotion Discrimination Task
**pRBD**	0.030	1.000	0.024	0.878	Total Correct Responses - Blocks Task
**Residual**	17.330	14.000			Total Correct Responses - Blocks Task
**pRBD**	9.561	1.000	25.631	0.000	Total Correct Responses - Target Detection Task
**Residual**	5.222	14.000			Total Correct Responses - Target Detection Task
**pRBD**	1.951	1.000	1.647	0.220	Total Correct Responses - Switching Stroop Task
**Residual**	16.587	14.000			Total Correct Responses - Switching Stroop Task
**pRBD**	1.876	1.000	1.944	0.185	Total Correct Responses - Trail Making Task
**Residual**	13.507	14.000			Total Correct Responses - Trail Making Task
**pRBD**	0.737	1.000	1.489	0.243	Total Correct Responses - Spatial Span Task
**Residual**	6.930	14.000			Total Correct Responses - Spatial Span Task
**pRBD**	1.197	1.000	1.379	0.260	Total Correct Responses - Verbal Analogies Task
**Residual**	12.151	14.000			Total Correct Responses - Verbal Analogies Task
**pRBD**	0.796	1.000	0.996	0.335	Total Words Remembered - Word Definitions Task
**Residual**	11.196	14.000			Total Words Remembered - Word Definitions Task
**pRBD**	0.320	1.000	0.279	0.605	Total Correct Responses - Paired Associates Learning Task
**Residual**	16.038	14.000			Total Correct Responses - Paired Associates Learning Task
**pRBD**	0.229	1.000	0.213	0.651	Percentage Correct Cards - Card Pairs Task
**Residual**	15.060	14.000			Percentage Correct Cards - Card Pairs Task
**pRBD**	0.024	1.000	0.027	0.871	Total Achieved - Four Towers Task
**Residual**	12.271	14.000			Total Achieved - Four Towers Task
**pRBD**	0.218	1.000	0.434	0.521	Total Correct Responses - Digit Span Task
**Residual**	7.047	14.000			Total Correct Responses - Digit Span Task
**pRBD**	0.049	1.000	0.094	0.764	Total Achieved - Tower of London Task
**Residual**	7.266	14.000			Total Achieved - Tower of London Task
**pRBD**	7.002	1.000	9.680	0.008	Mean Reaction Time - Motor Control Task
**Residual**	10.127	14.000			Mean Reaction Time - Motor Control Task
**pRBD**	0.301	1.000	0.317	0.582	Median Reaction Time - Simple Reaction Time Task
**Residual**	13.299	14.000			Median Reaction Time - Simple Reaction Time Task
**pRBD**	0.510	1.000	0.896	0.360	Total Errors - Picture Completion Task
**Residual**	7.970	14.000			Total Errors - Picture Completion Task

**pRBD**	1.795	1.000	2.829	0.115	Accuracy Composite
**Residual**	8.883	14.000			Accuracy Composite
**pRBD**	3.368	1.000	3.732	0.074	Immediate Prospective Memory - Median Reaction Time
**Residual**	12.633	14.000			Immediate Prospective Memory - Median Reaction Time
**pRBD**	5.059	1.000	5.776	0.031	Delayed Prospective Memory - Median Reaction Time
**Residual**	12.263	14.000			Delayed Prospective Memory - Median Reaction Time
**pRBD**	0.857	1.000	1.138	0.304	Manipulations 2D - Median Reaction Time
**Residual**	10.540	14.000			Manipulations 2D - Median Reaction Time
**pRBD**	7.212	1.000	13.654	0.002	Emotion Discrimination - Median Reaction Time
**Residual**	7.395	14.000			Emotion Discrimination - Median Reaction Time
**pRBD**	0.761	1.000	0.892	0.361	Blocks - Median Reaction Time
**Residual**	11.952	14.000			Blocks - Median Reaction Time
**pRBD**	11.339	1.000	27.772	0.000	Target Detection - Mean Reaction Time
**Residual**	5.716	14.000			Target Detection - Mean Reaction Time
**pRBD**	1.719	1.000	1.438	0.250	Trail Making - Mean Number Reaction Time
**Residual**	16.736	14.000			Trail Making - Mean Number Reaction Time
**pRBD**	1.858	1.000	2.018	0.177	Trail Making - Mean Letter Reaction Time
**Residual**	12.888	14.000			Trail Making - Mean Letter Reaction Time
**pRBD**	0.533	1.000	0.461	0.508	Trail Making - Number/Letter Cost
**Residual**	16.215	14.000			Trail Making - Number/Letter Cost
**pRBD**	1.927	1.000	2.340	0.148	Switching Stroop - Median Reaction Time
**Residual**	11.525	14.000			Switching Stroop - Median Reaction Time
**pRBD**	3.104	1.000	2.553	0.132	Switching Stroop - Mean Incongruent Accuracy
**Residual**	17.016	14.000			Switching Stroop - Mean Incongruent Accuracy
**pRBD**	1.652	1.000	2.398	0.144	Switching Stroop - Median Incongruent Reaction Time
**Residual**	9.640	14.000			Switching Stroop - Median Incongruent Reaction Time
**pRBD**	0.022	1.000	0.016	0.902	Switching Stroop - Mean Switch Accuracy
**Residual**	19.359	14.000			Switching Stroop - Mean Switch Accuracy
**pRBD**	2.554	1.000	2.638	0.127	Switching Stroop - Median Switch Reaction Time
**Residual**	13.551	14.000			Switching Stroop - Median Switch Reaction Time
**pRBD**	2.877	1.000	2.270	0.154	Spatial Span - Median Reaction Time
**Residual**	17.742	14.000			Spatial Span - Median Reaction Time
**pRBD**	4.248	1.000	7.834	0.014	Verbal Analogies - Median Reaction Time
**Residual**	7.592	14.000			Verbal Analogies - Median Reaction Time
**pRBD**	4.283	1.000	7.841	0.014	Word Definitions - Median Reaction Time
**Residual**	7.647	14.000			Word Definitions - Median Reaction Time

pRBD	8.035	1.000	14.091	0.002	PAL - Median Reaction Time
Residual	7.983	14.000			PAL - Median Reaction Time
pRBD	0.383	1.000	0.422	0.527	Card Pairs - Median Array Time
Residual	12.700	14.000			Card Pairs - Median Array Time
pRBD	7.993	1.000	11.222	0.005	Picture Completion - Total Time Taken
Residual	9.971	14.000			Picture Completion - Total Time Taken
pRBD	4.066	1.000	4.589	0.050	Four Towers - Median Reaction Time
Residual	12.403	14.000			Four Towers - Median Reaction Time
pRBD	0.566	1.000	0.999	0.335	Digit Span - Median Reaction Time
Residual	7.938	14.000			Digit Span - Median Reaction Time
pRBD	0.107	1.000	0.088	0.771	Tower of London - Median Reaction Time
Residual	16.919	14.000			Tower of London - Median Reaction Time
pRBD	0.070	1.000	0.060	0.809	Motor Control - Mean Euclidean Distance from Target
Residual	16.259	14.000			Motor Control - Mean Euclidean Distance from Target
pRBD	0.216	1.000	0.193	0.667	Simple Reaction Time - Median Reaction Time
Residual	15.668	14.000			Simple Reaction Time - Median Reaction Time
pRBD	8.379	1.000	10.294	0.006	RT Composite
Residual	11.396	14.000			RT Composite

Supplementary Table 18. ANOVA results looking at the significance of effect size differences in patients with PD&pRBD relative to iRBD patients

5. Effect size differences of PDonly, PD+pRBD, iRBD relative to controls

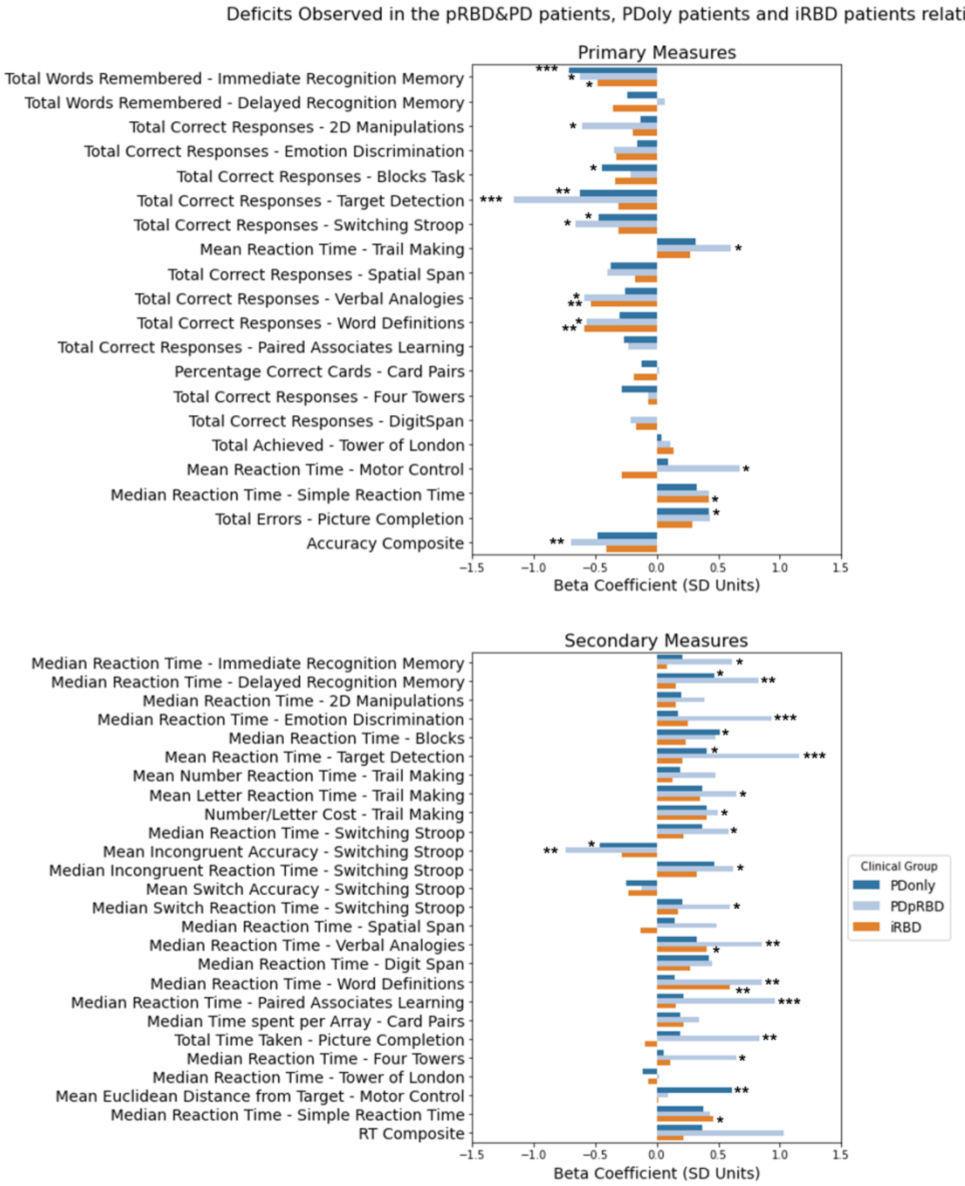

Supplementary Figure 9. Effect size differences in primary and secondary measures of task performance in patients with PDonly, PD and pRBD and iRBD patients relative to controls.

index	params	task
PDpRBD	-0.623	Total Words Remembered - Immediate Recognition Memory
PDonly	-0.715	Total Words Remembered - Immediate Recognition Memory
iRBD	-0.479	Total Words Remembered - Immediate Recognition Memory
PDpRBD	0.069	Total Words Remembered - Delayed Recognition Memory
PDonly	-0.240	Total Words Remembered - Delayed Recognition Memory
iRBD	-0.360	Total Words Remembered - Delayed Recognition Memory
PDpRBD	-0.602	Total Correct Responses - 2D Manipulations Task

PDonly	-0.132	Total Correct Responses - 2D Manipulations Task
iRBD	-0.194	Total Correct Responses - 2D Manipulations Task
PDpRBD	-0.346	Total Correct Responses - Emotion Discrimination Task
PDonly	-0.161	Total Correct Responses - Emotion Discrimination Task
iRBD	-0.326	Total Correct Responses - Emotion Discrimination Task
PDpRBD	-0.215	Total Correct Responses - Blocks Task
PDonly	-0.442	Total Correct Responses - Blocks Task
iRBD	-0.338	Total Correct Responses - Blocks Task
PDpRBD	-1.163	Total Correct Responses - Target Detection Task
PDonly	-0.628	Total Correct Responses - Target Detection Task
iRBD	-0.308	Total Correct Responses - Target Detection Task
PDpRBD	-0.661	Total Correct Responses - Switching Stroop Task
PDonly	-0.475	Total Correct Responses - Switching Stroop Task
iRBD	-0.314	Total Correct Responses - Switching Stroop Task
PDpRBD	0.603	Total Correct Responses - Trail Making Task
PDonly	0.318	Total Correct Responses - Trail Making Task
iRBD	0.272	Total Correct Responses - Trail Making Task
PDpRBD	-0.405	Total Correct Responses - Spatial Span Task
PDonly	-0.375	Total Correct Responses - Spatial Span Task
iRBD	-0.180	Total Correct Responses - Spatial Span Task
PDpRBD	-0.590	Total Correct Responses - Verbal Analogies Task
PDonly	-0.255	Total Correct Responses - Verbal Analogies Task
iRBD	-0.533	Total Correct Responses - Verbal Analogies Task
PDpRBD	-0.574	Total Words Remembered - Word Definitions Task
PDonly	-0.302	Total Words Remembered - Word Definitions Task
iRBD	-0.586	Total Words Remembered - Word Definitions Task
PDpRBD	-0.230	Total Correct Responses - Paired Associates Learning Task
PDonly	-0.264	Total Correct Responses - Paired Associates Learning Task
iRBD	0.000	Total Correct Responses - Paired Associates Learning Task
PDpRBD	0.021	Percentage Correct Cards - Card Pairs Task
PDonly	-0.122	Percentage Correct Cards - Card Pairs Task
iRBD	-0.190	Percentage Correct Cards - Card Pairs Task
PDpRBD	-0.066	Total Achieved - Four Towers Task
PDonly	-0.287	Total Achieved - Four Towers Task
iRBD	-0.067	Total Achieved - Four Towers Task
PDpRBD	-0.212	Total Correct Responses - Digit Span Task
PDonly	0.000	Total Correct Responses - Digit Span Task
iRBD	-0.166	Total Correct Responses - Digit Span Task
PDpRBD	0.108	Total Achieved - Tower of London Task
PDonly	0.041	Total Achieved - Tower of London Task
iRBD	0.140	Total Achieved - Tower of London Task

PDpRBD	0.677	Mean Reaction Time - Motor Control Task
PDonly	0.092	Mean Reaction Time - Motor Control Task
iRBD	-0.286	Mean Reaction Time - Motor Control Task
PDpRBD	0.427	Median Reaction Time - Simple Reaction Time Task
PDonly	0.326	Median Reaction Time - Simple Reaction Time Task
iRBD	0.425	Median Reaction Time - Simple Reaction Time Task
PDpRBD	0.436	Total Errors - Picture Completion Task
PDonly	0.423	Total Errors - Picture Completion Task
iRBD	0.288	Total Errors - Picture Completion Task
PDpRBD	-0.692	Accuracy Composite
PDonly	-0.477	Accuracy Composite
iRBD	-0.410	Accuracy Composite
PDpRBD	0.608	Immediate Prospective Memory - Median Reaction Time
PDonly	0.209	Immediate Prospective Memory - Median Reaction Time
iRBD	0.081	Immediate Prospective Memory - Median Reaction Time
PDpRBD	0.831	Delayed Prospective Memory - Median Reaction Time
PDonly	0.467	Delayed Prospective Memory - Median Reaction Time
iRBD	0.153	Delayed Prospective Memory - Median Reaction Time
PDpRBD	0.390	Manipulations 2D - Median Reaction Time
PDonly	0.201	Manipulations 2D - Median Reaction Time
iRBD	0.156	Manipulations 2D - Median Reaction Time
PDpRBD	0.935	Emotion Discrimination - Median Reaction Time
PDonly	0.170	Emotion Discrimination - Median Reaction Time
iRBD	0.251	Emotion Discrimination - Median Reaction Time
PDpRBD	0.480	Blocks - Median Reaction Time
PDonly	0.512	Blocks - Median Reaction Time
iRBD	0.232	Blocks - Median Reaction Time
PDpRBD	1.156	Target Detection - Mean Reaction Time
PDonly	0.410	Target Detection - Mean Reaction Time
iRBD	0.207	Target Detection - Mean Reaction Time
PDpRBD	0.481	Trail Making - Mean Number Reaction Time
PDonly	0.187	Trail Making - Mean Number Reaction Time
iRBD	0.130	Trail Making - Mean Number Reaction Time
PDpRBD	0.646	Trail Making - Mean Letter Reaction Time
PDonly	0.369	Trail Making - Mean Letter Reaction Time
iRBD	0.355	Trail Making - Mean Letter Reaction Time
PDpRBD	0.494	Trail Making - Number/Letter Cost
PDonly	0.406	Trail Making - Number/Letter Cost
iRBD	0.406	Trail Making - Number/Letter Cost
PDpRBD	0.581	Switching Stroop - Median Reaction Time
PDonly	0.369	Switching Stroop - Median Reaction Time

iRBD	0.218	Switching Stroop - Median Reaction Time
PDpRBD	-0.745	Switching Stroop - Mean Incongruent Accuracy
PDonly	-0.461	Switching Stroop - Mean Incongruent Accuracy
iRBD	-0.284	Switching Stroop - Mean Incongruent Accuracy
PDpRBD	0.620	Switching Stroop - Median Incongruent Reaction Time
PDonly	0.470	Switching Stroop - Median Incongruent Reaction Time
iRBD	0.329	Switching Stroop - Median Incongruent Reaction Time
PDpRBD	-0.123	Switching Stroop - Mean Switch Accuracy
PDonly	-0.246	Switching Stroop - Mean Switch Accuracy
iRBD	-0.228	Switching Stroop - Mean Switch Accuracy
PDpRBD	0.590	Switching Stroop - Median Switch Reaction Time
PDonly	0.208	Switching Stroop - Median Switch Reaction Time
iRBD	0.176	Switching Stroop - Median Switch Reaction Time
PDpRBD	0.491	Spatial Span - Median Reaction Time
PDonly	0.146	Spatial Span - Median Reaction Time
iRBD	-0.135	Spatial Span - Median Reaction Time
PDpRBD	0.854	Verbal Analogies - Median Reaction Time
PDonly	0.324	Verbal Analogies - Median Reaction Time
iRBD	0.405	Verbal Analogies - Median Reaction Time
PDpRBD	0.449	Digit Span - Median Reaction Time
PDonly	0.420	Digit Span - Median Reaction Time
iRBD	0.272	Digit Span - Median Reaction Time
PDpRBD	0.855	Word Definitions - Median Reaction Time
PDonly	0.150	Word Definitions - Median Reaction Time
iRBD	0.598	Word Definitions - Median Reaction Time
PDpRBD	0.958	PAL - Median Reaction Time
PDonly	0.222	PAL - Median Reaction Time
iRBD	0.159	PAL - Median Reaction Time
PDpRBD	0.341	Card Pairs - Median Array Time
PDonly	0.193	Card Pairs - Median Array Time
iRBD	0.217	Card Pairs - Median Array Time
PDpRBD	0.835	Picture Completion - Total Time Taken
PDonly	0.194	Picture Completion - Total Time Taken
iRBD	-0.092	Picture Completion - Total Time Taken
PDpRBD	0.643	Four Towers - Median Reaction Time
PDonly	0.052	Four Towers - Median Reaction Time
iRBD	0.109	Four Towers - Median Reaction Time
PDpRBD	0.021	Tower of London - Median Reaction Time
PDonly	-0.116	Tower of London - Median Reaction Time
iRBD	-0.065	Tower of London - Median Reaction Time
PDpRBD	0.096	Motor Control - Mean Euclidean Distance from Target

PDonly	0.611	Motor Control - Mean Euclidean Distance from Target
iRBD	0.015	Motor Control - Mean Euclidean Distance from Target
PDpRBD	0.431	Simple Reaction Time - Median Reaction Time
PDonly	0.375	Simple Reaction Time - Median Reaction Time
iRBD	0.462	Simple Reaction Time - Median Reaction Time
PDpRBD	1.032	RT composite
PDonly	0.371	RT composite
iRBD	0.214	RT composite

Supplementary Table 19. Beta coefficients representing the effect size differences in patients with PD&pRBD , PD only and iRBD relative to healthy controls

	sum_sq	df	F	PR(>F)	task
**PDpRBD**	4.461	1.000	5.024	0.026	Total Words Remembered - Immediate Recognition Memory
**PDonly**	11.246	1.000	12.664	0.000	Total Words Remembered - Immediate Recognition Memory
**iRBD**	5.839	1.000	6.576	0.011	Total Words Remembered - Immediate Recognition Memory
**Residual**	135.872	153.000			Total Words Remembered - Immediate Recognition Memory
**PDpRBD**	0.055	1.000	0.059	0.808	Total Words Remembered - Delayed Recognition Memory
**PDonly**	1.259	1.000	1.366	0.244	Total Words Remembered - Delayed Recognition Memory
**iRBD**	3.239	1.000	3.516	0.063	Total Words Remembered - Delayed Recognition Memory
**Residual**	139.094	151.000			Total Words Remembered - Delayed Recognition Memory
**PDpRBD**	4.167	1.000	4.390	0.038	Total Correct Responses - 2D Manipulations Task
**PDonly**	0.383	1.000	0.404	0.526	Total Correct Responses - 2D Manipulations Task
**iRBD**	0.961	1.000	1.013	0.316	Total Correct Responses - 2D Manipulations Task
**Residual**	145.232	153.000			Total Correct Responses - 2D Manipulations Task
**PDpRBD**	1.373	1.000	1.502	0.222	Total Correct Responses - Emotion Discrimination Task
**PDonly**	0.569	1.000	0.622	0.431	Total Correct Responses - Emotion Discrimination Task
**iRBD**	2.713	1.000	2.969	0.087	Total Correct Responses - Emotion Discrimination Task
**Residual**	139.825	153.000			Total Correct Responses - Emotion Discrimination Task
**PDpRBD**	0.530	1.000	0.581	0.447	Total Correct Responses - Blocks Task
**PDonly**	4.258	1.000	4.670	0.032	Total Correct Responses - Blocks Task
**iRBD**	2.874	1.000	3.152	0.078	Total Correct Responses - Blocks Task
**Residual**	138.597	152.000			Total Correct Responses - Blocks Task
**PDpRBD**	15.523	1.000	17.832	0.000	Total Correct Responses - Target Detection Task
**PDonly**	8.698	1.000	9.992	0.002	Total Correct Responses - Target Detection Task
**iRBD**	2.408	1.000	2.766	0.098	Total Correct Responses - Target Detection Task
**Residual**	133.186	153.000			Total Correct Responses - Target Detection Task
**PDpRBD**	4.998	1.000	5.593	0.019	Total Correct Responses - Switching Stroop Task
**PDonly**	4.849	1.000	5.426	0.021	Total Correct Responses - Switching Stroop Task
**iRBD**	2.439	1.000	2.729	0.101	Total Correct Responses - Switching Stroop Task

**Residual**	133.146	149.000			Total Correct Responses - Switching Stroop Task
**PDpRBD**	4.150	1.000	4.351	0.039	Total Correct Responses - Trail Making Task
**PDonly**	2.179	1.000	2.284	0.133	Total Correct Responses - Trail Making Task
**iRBD**	1.829	1.000	1.918	0.168	Total Correct Responses - Trail Making Task
**Residual**	142.127	149.000			Total Correct Responses - Trail Making Task
**PDpRBD**	1.877	1.000	1.976	0.162	Total Correct Responses - Spatial Span Task
**PDonly**	3.073	1.000	3.236	0.074	Total Correct Responses - Spatial Span Task
**iRBD**	0.819	1.000	0.863	0.355	Total Correct Responses - Spatial Span Task
**Residual**	144.371	152.000			Total Correct Responses - Spatial Span Task
**PDpRBD**	3.977	1.000	4.355	0.039	Total Correct Responses - Verbal Analogies Task
**PDonly**	1.423	1.000	1.559	0.214	Total Correct Responses - Verbal Analogies Task
**iRBD**	7.155	1.000	7.836	0.006	Total Correct Responses - Verbal Analogies Task
**Residual**	138.805	152.000			Total Correct Responses - Verbal Analogies Task
**PDpRBD**	3.759	1.000	4.069	0.045	Total Words Remembered - Word Definitions Task
**PDonly**	1.991	1.000	2.156	0.144	Total Words Remembered - Word Definitions Task
**iRBD**	8.643	1.000	9.356	0.003	Total Words Remembered - Word Definitions Task
**Residual**	140.415	152.000			Total Words Remembered - Word Definitions Task
**PDpRBD**	0.604	1.000	0.628	0.429	Total Correct Responses - Paired Associates Learning Task
**PDonly**	1.495	1.000	1.555	0.214	Total Correct Responses - Paired Associates Learning Task
**iRBD**	0.000	1.000	0.000	1.000	Total Correct Responses - Paired Associates Learning Task
**Residual**	143.320	149.000			Total Correct Responses - Paired Associates Learning Task
**PDpRBD**	0.005	1.000	0.005	0.943	Percentage Correct Cards - Card Pairs Task
**PDonly**	0.319	1.000	0.326	0.569	Percentage Correct Cards - Card Pairs Task
**iRBD**	0.882	1.000	0.899	0.345	Percentage Correct Cards - Card Pairs Task
**Residual**	145.166	148.000			Percentage Correct Cards - Card Pairs Task
**PDpRBD**	0.050	1.000	0.052	0.819	Total Achieved - Four Towers Task
**PDonly**	1.736	1.000	1.822	0.179	Total Achieved - Four Towers Task
**iRBD**	0.108	1.000	0.114	0.737	Total Achieved - Four Towers Task
**Residual**	138.223	145.000			Total Achieved - Four Towers Task
**PDpRBD**	0.514	1.000	0.535	0.465	Total Correct Responses - Digit Span Task
**PDonly**	0.000	1.000	0.000	0.999	Total Correct Responses - Digit Span Task
**iRBD**	0.693	1.000	0.723	0.397	Total Correct Responses - Digit Span Task
**Residual**	145.786	152.000			Total Correct Responses - Digit Span Task
**PDpRBD**	0.134	1.000	0.140	0.709	Total Achieved - Tower of London Task
**PDonly**	0.037	1.000	0.039	0.845	Total Achieved - Tower of London Task
**iRBD**	0.492	1.000	0.515	0.474	Total Achieved - Tower of London Task
**Residual**	144.270	151.000			Total Achieved - Tower of London Task
**PDpRBD**	5.291	1.000	5.908	0.016	Mean Reaction Time - Motor Control Task
**PDonly**	0.187	1.000	0.209	0.648	Mean Reaction Time - Motor Control Task

**iRBD**	2.111	1.000	2.357	0.127	Mean Reaction Time - Motor Control Task
**Residual**	137.937	154.000			Mean Reaction Time - Motor Control Task
**PDpRBD**	2.069	1.000	2.214	0.139	Median Reaction Time - Simple Reaction Time Task
**PDonly**	2.272	1.000	2.431	0.121	Median Reaction Time - Simple Reaction Time Task
**iRBD**	4.460	1.000	4.774	0.030	Median Reaction Time - Simple Reaction Time Task
**Residual**	139.217	149.000			Median Reaction Time - Simple Reaction Time Task
**PDpRBD**	2.174	1.000	2.357	0.127	Total Errors - Picture Completion Task
**PDonly**	3.851	1.000	4.175	0.043	Total Errors - Picture Completion Task
**iRBD**	2.015	1.000	2.184	0.142	Total Errors - Picture Completion Task
**Residual**	135.602	147.000			Total Errors - Picture Completion Task
**PDpRBD**	5.420	1.000	7.048	0.009	Accuracy Composite
**PDonly**	4.742	1.000	6.166	0.014	Accuracy Composite
**iRBD**	3.981	1.000	5.176	0.024	Accuracy Composite
**Residual**	110.739	144.000			Accuracy Composite
**PDpRBD**	4.242	1.000	4.447	0.037	Immediate Prospective Memory - Median Reaction Time
**PDonly**	0.959	1.000	1.005	0.318	Immediate Prospective Memory - Median Reaction Time
**iRBD**	0.167	1.000	0.175	0.676	Immediate Prospective Memory - Median Reaction Time
**Residual**	145.954	153.000			Immediate Prospective Memory - Median Reaction Time
**PDpRBD**	7.892	1.000	8.559	0.004	Delayed Prospective Memory - Median Reaction Time
**PDonly**	4.765	1.000	5.168	0.024	Delayed Prospective Memory - Median Reaction Time
**iRBD**	0.587	1.000	0.637	0.426	Delayed Prospective Memory - Median Reaction Time
**Residual**	139.230	151.000			Delayed Prospective Memory - Median Reaction Time
**PDpRBD**	1.743	1.000	1.819	0.179	Manipulations 2D - Median Reaction Time
**PDonly**	0.889	1.000	0.928	0.337	Manipulations 2D - Median Reaction Time
**iRBD**	0.617	1.000	0.644	0.424	Manipulations 2D - Median Reaction Time
**Residual**	146.587	153.000			Manipulations 2D - Median Reaction Time
**PDpRBD**	10.040	1.000	11.371	0.001	Emotion Discrimination - Median Reaction Time
**PDonly**	0.636	1.000	0.720	0.397	Emotion Discrimination - Median Reaction Time
**iRBD**	1.604	1.000	1.817	0.180	Emotion Discrimination - Median Reaction Time
**Residual**	135.086	153.000			Emotion Discrimination - Median Reaction Time
**PDpRBD**	2.637	1.000	2.758	0.099	Blocks - Median Reaction Time
**PDonly**	5.716	1.000	5.977	0.016	Blocks - Median Reaction Time
**iRBD**	1.360	1.000	1.422	0.235	Blocks - Median Reaction Time
**Residual**	145.365	152.000			Blocks - Median Reaction Time
**PDpRBD**	15.355	1.000	17.526	0.000	Target Detection - Mean Reaction Time
**PDonly**	3.695	1.000	4.218	0.042	Target Detection - Mean Reaction Time
**iRBD**	1.086	1.000	1.239	0.267	Target Detection - Mean Reaction Time
**Residual**	134.047	153.000			Target Detection - Mean Reaction Time
**PDpRBD**	2.649	1.000	2.730	0.101	Trail Making - Mean Number Reaction Time

**PDonly**	0.755	1.000	0.778	0.379	Trail Making - Mean Number Reaction Time
**iRBD**	0.417	1.000	0.430	0.513	Trail Making - Mean Number Reaction Time
**Residual**	144.569	149.000			Trail Making - Mean Number Reaction Time
**PDpRBD**	4.773	1.000	5.074	0.026	Trail Making - Mean Letter Reaction Time
**PDonly**	2.935	1.000	3.120	0.079	Trail Making - Mean Letter Reaction Time
**iRBD**	3.116	1.000	3.312	0.071	Trail Making - Mean Letter Reaction Time
**Residual**	140.159	149.000			Trail Making - Mean Letter Reaction Time
**PDpRBD**	2.791	1.000	2.994	0.086	Trail Making - Number/Letter Cost
**PDonly**	3.554	1.000	3.813	0.053	Trail Making - Number/Letter Cost
**iRBD**	4.069	1.000	4.365	0.038	Trail Making - Number/Letter Cost
**Residual**	138.891	149.000			Trail Making - Number/Letter Cost
**PDpRBD**	3.862	1.000	4.179	0.043	Switching Stroop - Median Reaction Time
**PDonly**	2.933	1.000	3.174	0.077	Switching Stroop - Median Reaction Time
**iRBD**	1.174	1.000	1.270	0.262	Switching Stroop - Median Reaction Time
**Residual**	137.672	149.000			Switching Stroop - Median Reaction Time
**PDpRBD**	6.351	1.000	7.123	0.008	Switching Stroop - Mean Incongruent Accuracy
**PDonly**	4.581	1.000	5.138	0.025	Switching Stroop - Mean Incongruent Accuracy
**iRBD**	1.989	1.000	2.231	0.137	Switching Stroop - Mean Incongruent Accuracy
**Residual**	132.862	149.000			Switching Stroop - Mean Incongruent Accuracy
**PDpRBD**	4.392	1.000	4.795	0.030	Switching Stroop - Median Incongruent Reaction Time
**PDonly**	4.755	1.000	5.191	0.024	Switching Stroop - Median Incongruent Reaction Time
**iRBD**	2.675	1.000	2.920	0.090	Switching Stroop - Median Incongruent Reaction Time
**Residual**	136.502	149.000			Switching Stroop - Median Incongruent Reaction Time
**PDpRBD**	0.172	1.000	0.183	0.670	Switching Stroop - Mean Switch Accuracy
**PDonly**	1.304	1.000	1.388	0.241	Switching Stroop - Mean Switch Accuracy
**iRBD**	1.280	1.000	1.363	0.245	Switching Stroop - Mean Switch Accuracy
**Residual**	139.961	149.000			Switching Stroop - Mean Switch Accuracy
**PDpRBD**	3.974	1.000	4.221	0.042	Switching Stroop - Median Switch Reaction Time
**PDonly**	0.931	1.000	0.989	0.322	Switching Stroop - Median Switch Reaction Time
**iRBD**	0.762	1.000	0.809	0.370	Switching Stroop - Median Switch Reaction Time
**Residual**	140.274	149.000			Switching Stroop - Median Switch Reaction Time
**PDpRBD**	2.753	1.000	2.882	0.092	Spatial Span - Median Reaction Time
**PDonly**	0.465	1.000	0.486	0.487	Spatial Span - Median Reaction Time
**iRBD**	0.459	1.000	0.480	0.489	Spatial Span - Median Reaction Time
**Residual**	145.185	152.000			Spatial Span - Median Reaction Time
**PDpRBD**	8.343	1.000	9.081	0.003	Verbal Analogies - Median Reaction Time
**PDonly**	2.296	1.000	2.499	0.116	Verbal Analogies - Median Reaction Time
**iRBD**	4.124	1.000	4.489	0.036	Verbal Analogies - Median Reaction Time
**Residual**	139.641	152.000			Verbal Analogies - Median Reaction Time

**PDpRBD**	2.301	1.000	2.468	0.118	Digit Span - Median Reaction Time
**PDonly**	3.846	1.000	4.125	0.044	Digit Span - Median Reaction Time
**iRBD**	1.870	1.000	2.006	0.159	Digit Span - Median Reaction Time
**Residual**	141.712	152.000			Digit Span - Median Reaction Time
**PDpRBD**	8.364	1.000	9.546	0.002	Word Definitions - Median Reaction Time
**PDonly**	0.489	1.000	0.558	0.456	Word Definitions - Median Reaction Time
**iRBD**	8.993	1.000	10.264	0.002	Word Definitions - Median Reaction Time
**Residual**	133.179	152.000			Word Definitions - Median Reaction Time
**PDpRBD**	10.485	1.000	11.684	0.001	PAL - Median Reaction Time
**PDonly**	1.060	1.000	1.181	0.279	PAL - Median Reaction Time
**iRBD**	0.626	1.000	0.697	0.405	PAL - Median Reaction Time
**Residual**	133.706	149.000			PAL - Median Reaction Time
**PDpRBD**	1.330	1.000	1.397	0.239	Card Pairs - Median Array Time
**PDonly**	0.802	1.000	0.842	0.360	Card Pairs - Median Array Time
**iRBD**	1.152	1.000	1.210	0.273	Card Pairs - Median Array Time
**Residual**	140.883	148.000			Card Pairs - Median Array Time
**PDpRBD**	7.964	1.000	9.003	0.003	Picture Completion - Total Time Taken
**PDonly**	0.811	1.000	0.917	0.340	Picture Completion - Total Time Taken
**iRBD**	0.204	1.000	0.230	0.632	Picture Completion - Total Time Taken
**Residual**	130.043	147.000			Picture Completion - Total Time Taken
**PDpRBD**	4.706	1.000	5.327	0.022	Four Towers - Median Reaction Time
**PDonly**	0.056	1.000	0.064	0.801	Four Towers - Median Reaction Time
**iRBD**	0.285	1.000	0.323	0.571	Four Towers - Median Reaction Time
**Residual**	128.087	145.000			Four Towers - Median Reaction Time
**PDpRBD**	0.005	1.000	0.006	0.940	Tower of London - Median Reaction Time
**PDonly**	0.294	1.000	0.316	0.575	Tower of London - Median Reaction Time
**iRBD**	0.105	1.000	0.113	0.737	Tower of London - Median Reaction Time
**Residual**	140.332	151.000			Tower of London - Median Reaction Time
**PDpRBD**	0.107	1.000	0.117	0.733	Motor Control - Mean Euclidean Distance from Target
**PDonly**	8.308	1.000	9.066	0.003	Motor Control - Mean Euclidean Distance from Target
**iRBD**	0.006	1.000	0.006	0.937	Motor Control - Mean Euclidean Distance from Target
**Residual**	141.135	154.000			Motor Control - Mean Euclidean Distance from Target
**PDpRBD**	2.112	1.000	2.280	0.133	Simple Reaction Time - Median Reaction Time
**PDonly**	3.000	1.000	3.239	0.074	Simple Reaction Time - Median Reaction Time
**iRBD**	5.277	1.000	5.698	0.018	Simple Reaction Time - Median Reaction Time
**Residual**	137.974	149.000			Simple Reaction Time - Median Reaction Time
**PDpRBD**	11.989	1.000	13.996	0.000	RT composite
**PDonly**	2.840	1.000	3.315	0.071	RT composite
**iRBD**	1.073	1.000	1.253	0.265	RT composite

Residual	122.492	143.000			RT composite

Supplementary Table 20. ANOVA results looking at the significance of effect size differences in patients with PD&pRBD , PD only and iRBD relative to healthy controls

6. Task performance vs. Bradykinesia in PD

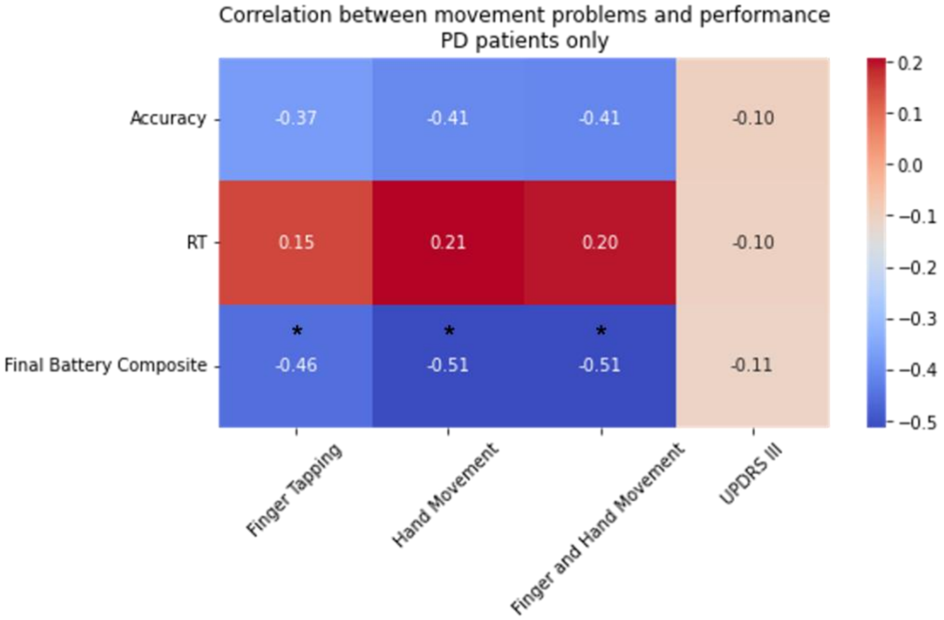

Supplementary Figure 10. Correlations between task performance composites and UPDRS III total as well as the subscales pertaining to finger and hand movement problems. The finger tapping composite is the average between the UPDRS III scale measuring finger tapping problems in the left finger summed with the one measuring finger tapping problems in the right finger. The hand movement composites represents the sum of the scales for both right and left hand movement problem scales summed with the pronation/supination scales from the UPDRS III. The hand and finger composite represents the sum of these two composite scores. * indicates statistically significant correlations.

7. Benchmarking sensitivity against MMSE

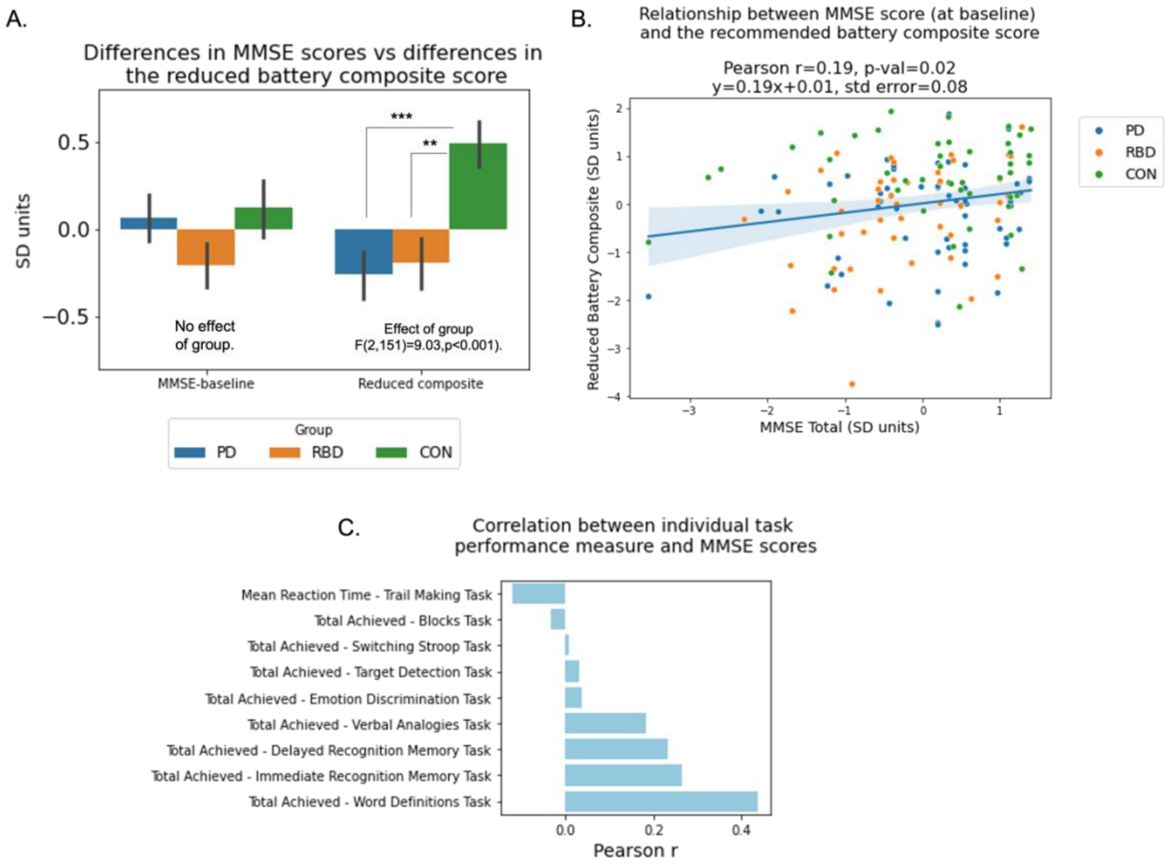

Supplementary Figure 11. Benchmarking against MMSE

We found that at baseline the MMSE was not able to differentiate between PD and RBD patients, or between either patient group and controls. In contrast, our battery was able to distinguish between controls and both patient groups, but not between the patient groups themselves. Overall there was a small but significant correlation between our battery's composite score and the MMSE total score. Following the application of a linear model with the individual task performance as predictors, and the MMSE total score as dependent variable, we also note there was a significant prediction (R-squared=0.305, p<0.001). The individual tasks that had the highest correlation with the MMSE total score were Word Definitions and the Immediate and Delayed Word Recognition Tasks.

	**coef**	**Std err**	**t**	**P>|t|**	**[0.025**	**0.975]**
**Intercept**	0.0348	0.088	0.397	0.692	-0.139	0.209
**Recognition Memory - immediate**	0.1835	0.099	1.862	0.066	-0.012	0.379
**Target Detection**	0.0440	0.098	0.448	0.655	-0.151	0.239
**Emotion Discrimination**	0.0086	0.095	0.091	0.927	-0.179	0.196

**Word Definitions**	0.4515	0.101	4.453	0.000	0.250	0.653
**Blocks**	-0.2337	0.108	-2.155	0.034	-0.449	-0.018
**Verbal Analogies**	-0.0111	0.105	-0.106	0.916	-0.220	0.198
**Recognition Memory - delayed**	0.1129	0.098	1.147	0.254	-0.083	0.308
**Switching Stroop**	-0.0176	0.106	-0.166	0.869	-0.229	0.193
**Trail Making**	-0.0660	0.100	-0.657	0.513	-0.265	0.133
**Number of observations**	103					
**Df residuals**	93					
**Df model**	9					
**R-squared**	0.305					
**P-value**	5.72e-05					

Supplementary Table 19. Predicting the baseline MMSE score from performance on the recommended selection of tasks.

8. Learning effects in the normative dataset

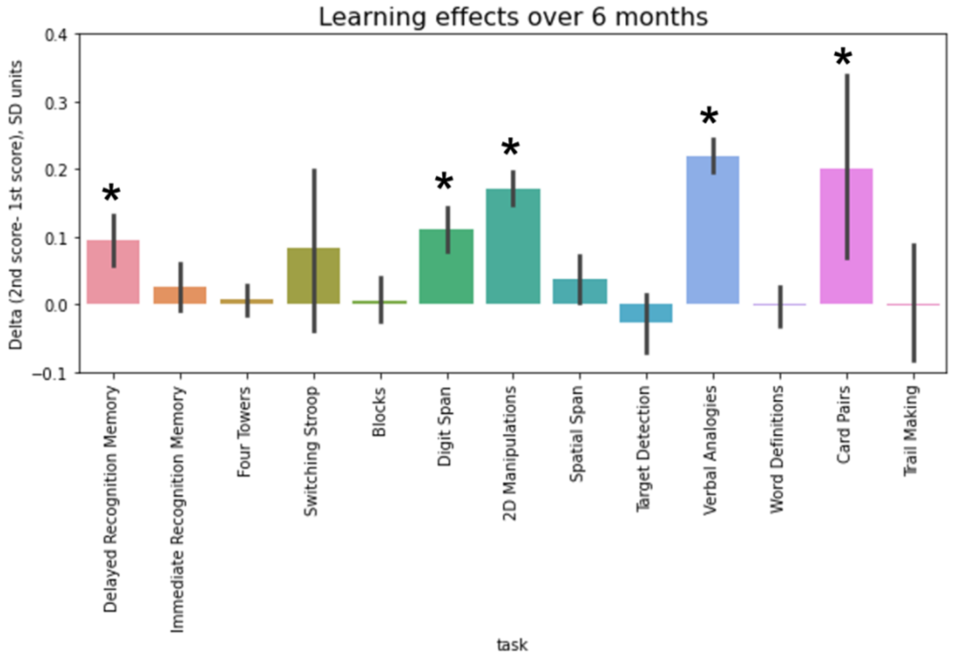

Supplementary Figure 12. Learning effects in the normative dataset over 6 months

Task	N Participants	t-statistic	p-value
Four Tower s	7070	-0.542	0.588
Recognition Memory Immediate	4306	-1.414	0.158
Recognition Memory Delayed	4016	-5.255	<0.001
Blocks	3823	-0.382	0.703
Tower of London	3787	-2.655	0.008
Digit Span	3779	-7.261	<0.001
Spatial Span	3729	-2.287	0.022
Verbal Analogies	3714	-17.489	<0.001
Target Detection	3691	1.347	0.178
Word Definitions	3685	0.123	0.902
Manipulations 2D	3674	-14.299	<0.001
Trail Making	288	0.038	0.970
Card Pairs	257	-3.189	0.002
Switching Stroop	233	-1.394	0.165

Supplementary Table 20. Statistics for the learning effects (repeated measures T-test).

9. Generalisability of results

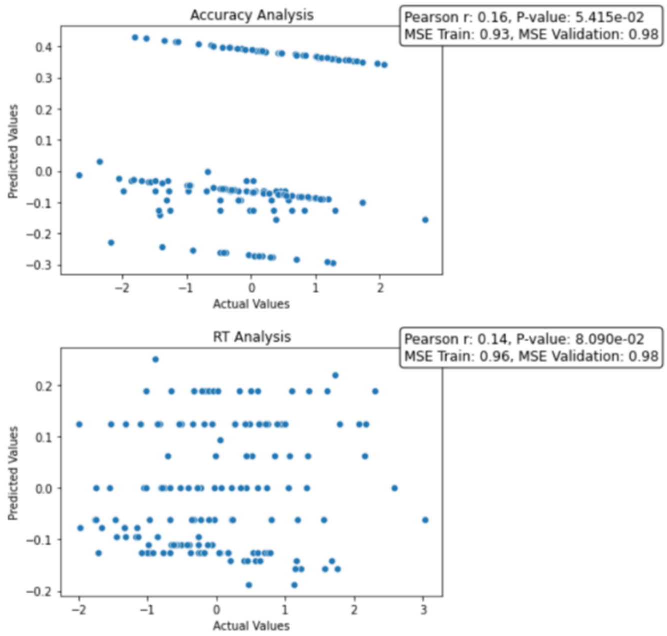

Supplementary Figure 13. Correlation between actual and predicted scores for Accuracy and RT after leave one out cross-validation.

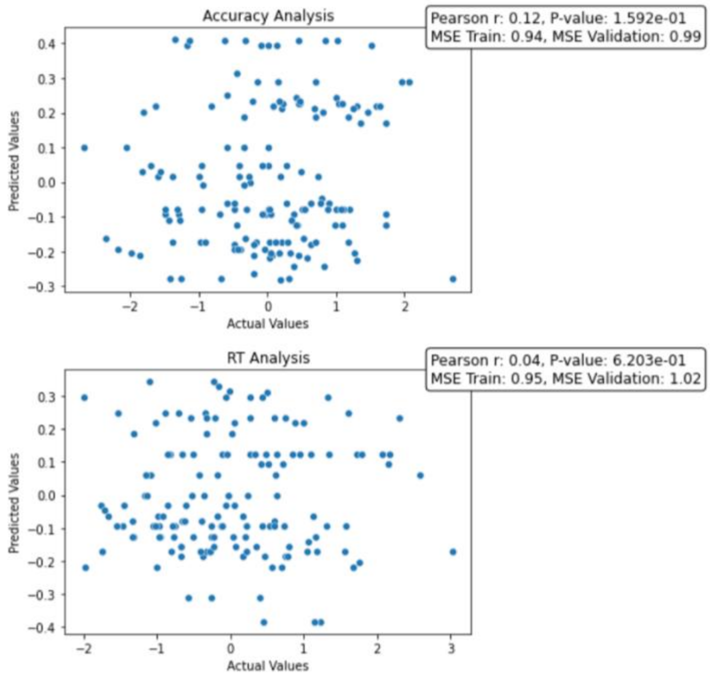

Supplementary Figure 14. Correlation between actual and predicted scores for Accuracy and RT after 25 fold cross-validation.

Running leave one out cross-validation or 25-fold cross-validation shows a significant correlation between observed and predicted scores when linear modelling cross group differences both in accuracy and response time composite scores.

10. Primary analyses supplementary tables

	sum_sq	df	F	PR(>F)	task
PD	12.244	1.000	13.844	0.000	Total Words Remembered - Immediate Recognition Memory
RBD	5.139	1.000	5.811	0.017	Total Words Remembered - Immediate Recognition Memory
Residual	140.621	159.000			Total Words Remembered - Immediate Recognition Memory
PD	1.367	1.000	1.451	0.230	Total Words Remembered - Delayed Recognition Memory
RBD	3.199	1.000	3.395	0.067	Total Words Remembered - Delayed Recognition Memory
Residual	147.929	157.000			Total Words Remembered - Delayed Recognition Memory
PD	2.630	1.000	2.774	0.098	Total Correct Responses - 2D Manipulations Task
RBD	0.979	1.000	1.032	0.311	Total Correct Responses - 2D Manipulations Task
Residual	150.717	159.000			Total Correct Responses - 2D Manipulations Task
PD	1.430	1.000	1.515	0.220	Total Correct Responses - Emotion Discrimination Task
RBD	2.927	1.000	3.101	0.080	Total Correct Responses - Emotion Discrimination Task
Residual	150.065	159.000			Total Correct Responses - Emotion Discrimination Task
PD	3.829	1.000	4.096	0.045	Total Correct Responses - Blocks Task
RBD	3.297	1.000	3.527	0.062	Total Correct Responses - Blocks Task
Residual	147.694	158.000			Total Correct Responses - Blocks Task
PD	14.204	1.000	16.273	0.000	Total Correct Responses - Target Detection Task
RBD	2.262	1.000	2.591	0.109	Total Correct Responses - Target Detection Task
Residual	138.783	159.000			Total Correct Responses - Target Detection Task
PD	7.350	1.000	8.023	0.005	Total Correct Responses - Switching Stroop Task
RBD	2.611	1.000	2.850	0.093	Total Correct Responses - Switching Stroop Task
Residual	142.003	155.000			Total Correct Responses - Switching Stroop Task
PD	4.587	1.000	4.912	0.028	Total Correct Responses - Trail Making Task
RBD	2.105	1.000	2.254	0.135	Total Correct Responses - Trail Making Task
Residual	144.727	155.000			Total Correct Responses - Trail Making Task
PD	3.062	1.000	3.245	0.074	Total Correct Responses - Spatial Span Task
RBD	0.785	1.000	0.832	0.363	Total Correct Responses - Spatial Span Task
Residual	149.079	158.000			Total Correct Responses - Spatial Span Task
PD	3.244	1.000	3.535	0.062	Total Correct Responses - Verbal Analogies Task
RBD	7.247	1.000	7.899	0.006	Total Correct Responses - Verbal Analogies Task
Residual	144.966	158.000			Total Correct Responses - Verbal Analogies Task
PD	4.524	1.000	4.980	0.027	Total Words Remembered - Word Definitions Task
RBD	8.301	1.000	9.138	0.003	Total Words Remembered - Word Definitions Task
Residual	143.524	158.000			Total Words Remembered - Word Definitions Task
PD	1.494	1.000	1.575	0.211	Total Correct Responses - Paired Associates Learning Task
RBD	0.003	1.000	0.003	0.953	Total Correct Responses - Paired Associates Learning Task

Residual	147.009	155.000			Total Correct Responses - Paired Associates Learning Task
PD	0.378	1.000	0.395	0.531	Percentage Correct Cards - Card Pairs Task
RBD	0.801	1.000	0.836	0.362	Percentage Correct Cards - Card Pairs Task
Residual	147.590	154.000			Percentage Correct Cards - Card Pairs Task
PD	1.549	1.000	1.630	0.204	Total Achieved - Four Towers Task
RBD	0.060	1.000	0.063	0.802	Total Achieved - Four Towers Task
Residual	142.566	150.000			Total Achieved - Four Towers Task
PD	0.275	1.000	0.287	0.593	Total Correct Responses - Digit Span Task
RBD	0.658	1.000	0.686	0.409	Total Correct Responses - Digit Span Task
Residual	151.509	158.000			Total Correct Responses - Digit Span Task
PD	0.000	1.000	0.000	0.995	Total Achieved - Tower of London Task
RBD	0.588	1.000	0.614	0.434	Total Achieved - Tower of London Task
Residual	150.416	157.000			Total Achieved - Tower of London Task
PD	1.507	1.000	1.641	0.202	Mean Reaction Time - Motor Control Task
RBD	1.979	1.000	2.154	0.144	Mean Reaction Time - Motor Control Task
Residual	146.989	160.000			Mean Reaction Time - Motor Control Task
PD	4.313	1.000	4.642	0.033	Median Reaction Time - Simple Reaction Time Task
RBD	4.044	1.000	4.353	0.039	Median Reaction Time - Simple Reaction Time Task
Residual	144.019	155.000			Median Reaction Time - Simple Reaction Time Task
PD	5.146	1.000	5.537	0.020	Total Errors - Picture Completion Task
RBD	1.834	1.000	1.973	0.162	Total Errors - Picture Completion Task
Residual	141.270	152.000			Total Errors - Picture Completion Task
PD	2.559	1.000	2.705	0.102	Immediate Prospective Memory - Median Reaction Time
RBD	0.105	1.000	0.111	0.740	Immediate Prospective Memory - Median Reaction Time
Residual	150.439	159.000			Immediate Prospective Memory - Median Reaction Time
PD	8.121	1.000	8.959	0.003	Delayed Prospective Memory - Median Reaction Time
RBD	0.485	1.000	0.535	0.466	Delayed Prospective Memory - Median Reaction Time
Residual	142.314	157.000			Delayed Prospective Memory - Median Reaction Time
PD	1.911	1.000	2.006	0.159	Manipulations 2D - Median Reaction Time
RBD	0.655	1.000	0.688	0.408	Manipulations 2D - Median Reaction Time
Residual	151.482	159.000			Manipulations 2D - Median Reaction Time
PD	4.467	1.000	4.772	0.030	Emotion Discrimination - Median Reaction Time
RBD	1.992	1.000	2.129	0.147	Emotion Discrimination - Median Reaction Time
Residual	148.824	159.000			Emotion Discrimination - Median Reaction Time
PD	6.115	1.000	6.606	0.011	Blocks - Median Reaction Time
RBD	1.407	1.000	1.520	0.219	Blocks - Median Reaction Time
Residual	146.261	158.000			Blocks - Median Reaction Time
PD	9.671	1.000	10.741	0.001	Target Detection - Mean Reaction Time

RBD	1.067	1.000	1.185	0.278	Target Detection - Mean Reaction Time
Residual	143.162	159.000			Target Detection - Mean Reaction Time
PD	2.088	1.000	2.197	0.140	Trail Making - Mean Number Reaction Time
RBD	0.358	1.000	0.377	0.540	Trail Making - Mean Number Reaction Time
Residual	147.316	155.000			Trail Making - Mean Number Reaction Time
PD	5.650	1.000	6.113	0.014	Trail Making - Mean Letter Reaction Time
RBD	3.549	1.000	3.840	0.052	Trail Making - Mean Letter Reaction Time
Residual	143.266	155.000			Trail Making - Mean Letter Reaction Time
PD	5.200	1.000	5.636	0.019	Trail Making - Number/Letter Cost
RBD	4.663	1.000	5.055	0.026	Trail Making - Number/Letter Cost
Residual	143.003	155.000			Trail Making - Number/Letter Cost
PD	4.546	1.000	4.863	0.029	Switching Stroop - Median Reaction Time
RBD	1.135	1.000	1.214	0.272	Switching Stroop - Median Reaction Time
Residual	144.891	155.000			Switching Stroop - Median Reaction Time
PD	7.593	1.000	8.297	0.005	Switching Stroop - Mean Incongruent Accuracy
RBD	2.003	1.000	2.189	0.141	Switching Stroop - Mean Incongruent Accuracy
Residual	141.847	155.000			Switching Stroop - Mean Incongruent Accuracy
PD	6.357	1.000	6.892	0.010	Switching Stroop - Median Incongruent Reaction Time
RBD	2.665	1.000	2.889	0.091	Switching Stroop - Median Incongruent Reaction Time
Residual	142.973	155.000			Switching Stroop - Median Incongruent Reaction Time
PD	1.356	1.000	1.425	0.234	Switching Stroop - Mean Switch Accuracy
RBD	1.382	1.000	1.454	0.230	Switching Stroop - Mean Switch Accuracy
Residual	147.421	155.000			Switching Stroop - Mean Switch Accuracy
PD	2.833	1.000	2.995	0.086	Switching Stroop - Median Switch Reaction Time
RBD	0.736	1.000	0.778	0.379	Switching Stroop - Median Switch Reaction Time
Residual	146.610	155.000			Switching Stroop - Median Switch Reaction Time
PD	1.860	1.000	1.989	0.160	Spatial Span - Median Reaction Time
RBD	0.443	1.000	0.474	0.492	Spatial Span - Median Reaction Time
Residual	147.773	158.000			Spatial Span - Median Reaction Time
PD	6.316	1.000	6.873	0.010	Verbal Analogies - Median Reaction Time
RBD	4.633	1.000	5.041	0.026	Verbal Analogies - Median Reaction Time
Residual	145.204	158.000			Verbal Analogies - Median Reaction Time
PD	3.573	1.000	3.911	0.050	Word Definitions - Median Reaction Time
RBD	7.856	1.000	8.599	0.004	Word Definitions - Median Reaction Time
Residual	144.355	158.000			Word Definitions - Median Reaction Time
PD	5.473	1.000	5.900	0.016	PAL - Median Reaction Time
RBD	0.785	1.000	0.846	0.359	PAL - Median Reaction Time
Residual	143.789	155.000			PAL - Median Reaction Time

PD	2.208	1.000	2.328	0.129	Card Pairs - Median Array Time
RBD	1.295	1.000	1.365	0.244	Card Pairs - Median Array Time
Residual	146.078	154.000			Card Pairs - Median Array Time
PD	3.608	1.000	3.923	0.049	Picture Completion - Total Time Taken
RBD	0.241	1.000	0.262	0.609	Picture Completion - Total Time Taken
Residual	139.795	152.000			Picture Completion - Total Time Taken
PD	1.189	1.000	1.244	0.266	Four Towers - Median Reaction Time
RBD	0.434	1.000	0.454	0.501	Four Towers - Median Reaction Time
Residual	143.302	150.000			Four Towers - Median Reaction Time
PD	5.336	1.000	5.733	0.018	Digit Span - Median Reaction Time
RBD	1.823	1.000	1.958	0.164	Digit Span - Median Reaction Time
Residual	147.060	158.000			Digit Span - Median Reaction Time
PD	0.409	1.000	0.425	0.515	Tower of London - Median Reaction Time
RBD	0.066	1.000	0.069	0.793	Tower of London - Median Reaction Time
Residual	151.003	157.000			Tower of London - Median Reaction Time
PD	5.004	1.000	5.415	0.021	Motor Control - Mean Euclidean Distance from Target
RBD	0.019	1.000	0.021	0.885	Motor Control - Mean Euclidean Distance from Target
Residual	147.857	160.000			Motor Control - Mean Euclidean Distance from Target
PD	5.145	1.000	5.578	0.019	Simple Reaction Time - Median Reaction Time
RBD	4.822	1.000	5.228	0.024	Simple Reaction Time - Median Reaction Time
Residual	142.974	155.000			Simple Reaction Time - Median Reaction Time

Supplementary Table 21. ANOVA analysis for primary and secondary task measures.

index	params	task
60s	0.258	Mean Reaction Time - Motor Control Task
70s	0.473	Mean Reaction Time - Motor Control Task
80s	0.640	Mean Reaction Time - Motor Control Task
60s	-0.126	Total Words Remembered - Immediate Recognition Memory
70s	-0.239	Total Words Remembered - Immediate Recognition Memory
80s	-0.494	Total Words Remembered - Immediate Recognition Memory
60s	-0.146	Total Words Remembered - Delayed Recognition Memory
70s	-0.244	Total Words Remembered - Delayed Recognition Memory
80s	-0.514	Total Words Remembered - Delayed Recognition Memory
60s	-0.307	Total Correct Responses - Target Detection Task
70s	-0.595	Total Correct Responses - Target Detection Task
80s	-1.022	Total Correct Responses - Target Detection Task
60s	-0.113	Total Correct Responses - Emotion Discrimination Task
70s	-0.306	Total Correct Responses - Emotion Discrimination Task
80s	-0.551	Total Correct Responses - Emotion Discrimination Task
60s	-0.418	Total Correct Responses - 2D Manipulations Task

70s	-0.753	Total Correct Responses - 2D Manipulations Task
80s	-1.110	Total Correct Responses - 2D Manipulations Task
60s	-0.095	Total Correct Responses - Digit Span Task
70s	-0.196	Total Correct Responses - Digit Span Task
80s	-0.470	Total Correct Responses - Digit Span Task
60s	-0.331	Total Correct Responses - Spatial Span Task
70s	-0.576	Total Correct Responses - Spatial Span Task
80s	-0.888	Total Correct Responses - Spatial Span Task
60s	-0.286	Total Correct Responses - Blocks Task
70s	-0.541	Total Correct Responses - Blocks Task
80s	-0.825	Total Correct Responses - Blocks Task
60s	-0.177	Total Achieved - Tower of London Task
70s	-0.377	Total Achieved - Tower of London Task
80s	-0.619	Total Achieved - Tower of London Task
60s	-0.137	Total Correct Responses - Verbal Analogies Task
70s	-0.357	Total Correct Responses - Verbal Analogies Task
80s	-0.701	Total Correct Responses - Verbal Analogies Task
60s	0.327	Total Correct Responses - Word Definitions Task
70s	0.429	Total Correct Responses - Word Definitions Task
80s	0.496	Total Correct Responses - Word Definitions Task
60s	-0.175	Total Achieved - Faulty Towers Task
70s	-0.394	Total Achieved - Faulty Towers Task
80s	-0.611	Total Achieved - Faulty Towers Task
60s	0.266	Simple Reaction Time - Median Reaction Time
70s	0.516	Simple Reaction Time - Median Reaction Time
80s	1.043	Simple Reaction Time - Median Reaction Time
60s	-0.402	Total Correct Responses - Switching Stroop Task
70s	-0.638	Total Correct Responses - Switching Stroop Task
80s	-0.963	Total Correct Responses - Switching Stroop Task
60s	-0.337	Card Pairs - Median Array Time
70s	-0.641	Card Pairs - Median Array Time
80s	-1.070	Card Pairs - Median Array Time
60s	0.430	Trail Making - Mean Reaction Time
70s	0.961	Trail Making - Mean Reaction Time
80s	1.376	Trail Making - Mean Reaction Time
60s	-0.241	Total Correct Responses - Paired Associates Learning Task
70s	-0.458	Total Correct Responses - Paired Associates Learning Task
80s	-0.890	Total Correct Responses - Paired Associates Learning Task
60s	0.072	Picture Completion - Total Time Taken
70s	0.427	Picture Completion - Total Time Taken
80s	0.519	Picture Completion - Total Time Taken

60s	0.318	Immediate Recognition Memory - Median Reaction Time
70s	0.529	Immediate Recognition Memory - Median Reaction Time
80s	1.006	Immediate Recognition Memory - Median Reaction Time
60s	0.322	Delayed Recognition Memory - Median Reaction Time
70s	0.514	Delayed Recognition Memory - Median Reaction Time
80s	0.856	Delayed Recognition Memory - Median Reaction Time
60s	0.353	Target Detection - Mean Reaction Time
70s	0.583	Target Detection - Mean Reaction Time
80s	0.873	Target Detection - Mean Reaction Time
60s	0.273	Emotion Discrimination - Median Reaction Time
70s	0.521	Emotion Discrimination - Median Reaction Time
80s	0.939	Emotion Discrimination - Median Reaction Time
60s	0.298	Manipulations 2D - Median Reaction Time
70s	0.583	Manipulations 2D - Median Reaction Time
80s	0.956	Manipulations 2D - Median Reaction Time
60s	0.148	Spatial Span - Median Reaction Time
70s	0.231	Spatial Span - Median Reaction Time
80s	0.440	Spatial Span - Median Reaction Time
60s	0.291	Blocks - Median Reaction Time
70s	0.518	Blocks - Median Reaction Time
80s	0.836	Blocks - Median Reaction Time
60s	0.236	Verbal Analogies - Median Reaction Time
70s	0.556	Verbal Analogies - Median Reaction Time
80s	1.096	Verbal Analogies - Median Reaction Time
60s	-0.087	Word Definitions - Median Reaction Time
70s	-0.091	Word Definitions - Median Reaction Time
80s	0.050	Word Definitions - Median Reaction Time
60s	0.218	Digit Span - Median Reaction Time
70s	0.398	Digit Span - Median Reaction Time
80s	0.696	Digit Span - Median Reaction Time
60s	0.181	Tower of London - Median Reaction Time
70s	0.352	Tower of London - Median Reaction Time
80s	0.672	Tower of London - Median Reaction Time
60s	0.310	Four Towers - Median Reaction Time
70s	0.530	Four Towers - Median Reaction Time
80s	0.708	Four Towers - Median Reaction Time
60s	0.519	Switching Stroop - Median Reaction Time
70s	0.958	Switching Stroop - Median Reaction Time
80s	1.745	Switching Stroop - Median Reaction Time
60s	-0.402	Switching Stroop - Mean Incongruent Accuracy
70s	-0.652	Switching Stroop - Mean Incongruent Accuracy

80s	-1.052	Switching Stroop - Mean Incongruent Accuracy
60s	0.521	Switching Stroop - Median Incongruent Reaction Time
70s	0.984	Switching Stroop - Median Incongruent Reaction Time
80s	1.835	Switching Stroop - Median Incongruent Reaction Time
60s	-0.336	Switching Stroop - Mean Switch Accuracy
70s	-0.464	Switching Stroop - Mean Switch Accuracy
80s	-0.861	Switching Stroop - Mean Switch Accuracy
60s	0.427	Switching Stroop - Median Switch Reaction Time
70s	0.761	Switching Stroop - Median Switch Reaction Time
80s	1.608	Switching Stroop - Median Switch Reaction Time
60s	-0.051	Picture Completion - Total Time Taken
70s	-0.035	Picture Completion - Total Time Taken
80s	-0.004	Picture Completion - Total Time Taken
60s	0.447	Card Pairs - Median Array Time
70s	0.879	Card Pairs - Median Array Time
80s	1.405	Card Pairs - Median Array Time
60s	0.225	PAL - Median Reaction Time
70s	0.607	PAL - Median Reaction Time
80s	0.870	PAL - Median Reaction Time
60s	0.249	Simple Reaction Time - Median Reaction Time
70s	0.459	Simple Reaction Time - Median Reaction Time
80s	0.928	Simple Reaction Time - Median Reaction Time
60s	0.351	Trail Making - Mean Number Reaction Time
70s	0.893	Trail Making - Mean Number Reaction Time
80s	1.386	Trail Making - Mean Number Reaction Time
60s	0.397	Trail Making - Mean Letter Reaction Time
70s	0.821	Trail Making - Mean Letter Reaction Time
80s	1.109	Trail Making - Mean Letter Reaction Time
60s	0.214	Trail Making - Number/Letter Cost
70s	0.323	Trail Making - Number/Letter Cost
80s	0.306	Trail Making - Number/Letter Cost
60s	-0.080	Motor Control - Mean Euclidean Distance from Target
70s	-0.178	Motor Control - Mean Euclidean Distance from Target
80s	-0.144	Motor Control - Mean Euclidean Distance from Target

Supplementary Table 22. Beta coefficients for ageing – primary and secondary measures.

	sum_sq	df	F	PR(>F)	task
**decade60s**	105.884	1.000	109.023	0.000	Mean Reaction Time - Motor Control Task
**decade70s**	148.784	1.000	153.194	0.000	Mean Reaction Time - Motor Control Task
**decade80s**	26.642	1.000	27.432	0.000	Mean Reaction Time - Motor Control Task

Residual	7312.234	7529.000			Mean Reaction Time - Motor Control Task
decade60s	200.489	1.000	202.148	0.000	Total Words Remembered - Immediate Recognition Memory
decade70s	270.247	1.000	272.483	0.000	Total Words Remembered - Immediate Recognition Memory
decade80s	142.240	1.000	143.416	0.000	Total Words Remembered - Immediate Recognition Memory
Residual	56598.825	57067.000			Total Words Remembered - Immediate Recognition Memory
decade60s	260.463	1.000	262.900	0.000	Total Words Remembered - Delayed Recognition Memory
decade70s	264.513	1.000	266.987	0.000	Total Words Remembered - Delayed Recognition Memory
decade80s	145.742	1.000	147.105	0.000	Total Words Remembered - Delayed Recognition Memory
Residual	54623.111	55134.000			Total Words Remembered - Delayed Recognition Memory
decade60s	4808.033	1.000	5052.376	0.000	Total Correct Responses - Target Detection Task
decade70s	7382.052	1.000	7757.205	0.000	Total Correct Responses - Target Detection Task
decade80s	2658.334	1.000	2793.429	0.000	Total Correct Responses - Target Detection Task
Residual	223960.420	235342.000			Total Correct Responses - Target Detection Task
decade60s	438.849	1.000	443.415	0.000	Total Correct Responses - Emotion Discrimination Task
decade70s	1130.670	1.000	1142.434	0.000	Total Correct Responses - Emotion Discrimination Task
decade80s	419.458	1.000	423.822	0.000	Total Correct Responses - Emotion Discrimination Task
Residual	155383.224	157000.000			Total Correct Responses - Emotion Discrimination Task
decade60s	8909.382	1.000	9653.714	0.000	Total Correct Responses - 2D Manipulations Task
decade70s	11772.376	1.000	12755.895	0.000	Total Correct Responses - 2D Manipulations Task
decade80s	3126.421	1.000	3387.617	0.000	Total Correct Responses - 2D Manipulations Task
Residual	216519.912	234609.000			Total Correct Responses - 2D Manipulations Task
decade60s	466.427	1.000	469.261	0.000	Total Correct Responses - Digit Span Task
decade70s	804.626	1.000	809.514	0.000	Total Correct Responses - Digit Span Task
decade80s	555.195	1.000	558.568	0.000	Total Correct Responses - Digit Span Task
Residual	235410.782	236841.000			Total Correct Responses - Digit Span Task
decade60s	5618.340	1.000	5893.937	0.000	Total Correct Responses - Spatial Span Task
decade70s	6954.035	1.000	7295.152	0.000	Total Correct Responses - Spatial Span Task
decade80s	2005.533	1.000	2103.910	0.000	Total Correct Responses - Spatial Span Task
Residual	225371.812	236427.000			Total Correct Responses - Spatial Span Task
decade60s	4289.591	1.000	4464.795	0.000	Total Correct Responses - Blocks Task
decade70s	6310.050	1.000	6567.778	0.000	Total Correct Responses - Blocks Task
decade80s	1817.908	1.000	1892.159	0.000	Total Correct Responses - Blocks Task
Residual	231370.863	240821.000			Total Correct Responses - Blocks Task
decade60s	1623.512	1.000	1653.704	0.000	Total Achieved - Tower of London Task
decade70s	3021.829	1.000	3078.026	0.000	Total Achieved - Tower of London Task
decade80s	1009.448	1.000	1028.220	0.000	Total Achieved - Tower of London Task

Residual	234784.722	239151.000			Total Achieved - Tower of London Task
decade60s	959.797	1.000	975.985	0.000	Total Correct Responses - Verbal Analogies Task
decade70s	2674.422	1.000	2719.530	0.000	Total Correct Responses - Verbal Analogies Task
decade80s	1265.086	1.000	1286.423	0.000	Total Correct Responses - Verbal Analogies Task
Residual	232544.789	236467.000			Total Correct Responses - Verbal Analogies Task
decade60s	5463.278	1.000	5644.525	0.000	Total Words Remembered - Word Definitions Task
decade70s	3842.382	1.000	3969.855	0.000	Total Words Remembered - Word Definitions Task
decade80s	627.130	1.000	647.935	0.000	Total Words Remembered - Word Definitions Task
Residual	227772.541	235329.000			Total Words Remembered - Word Definitions Task
decade60s	523.216	1.000	533.482	0.000	Total Achieved - Four Towers Task
decade70s	1101.788	1.000	1123.406	0.000	Total Achieved - Four Towers Task
decade80s	336.373	1.000	342.973	0.000	Total Achieved - Four Towers Task
Residual	76544.111	78046.000			Total Achieved - Four Towers Task
decade60s	90.996	1.000	94.750	0.000	Median Reaction Time - Simple Reaction Time Task
decade70s	168.478	1.000	175.428	0.000	Median Reaction Time - Simple Reaction Time Task
decade80s	64.926	1.000	67.605	0.000	Median Reaction Time - Simple Reaction Time Task
Residual	5802.624	6042.000			Median Reaction Time - Simple Reaction Time Task
decade60s	346.860	1.000	370.529	0.000	Total Correct Responses - Switching Stroop Task
decade70s	431.993	1.000	461.471	0.000	Total Correct Responses - Switching Stroop Task
decade80s	135.385	1.000	144.623	0.000	Total Correct Responses - Switching Stroop Task
Residual	9491.337	10139.000			Total Correct Responses - Switching Stroop Task
decade60s	246.794	1.000	262.855	0.000	Percentage Correct Cards - Card Pairs Task
decade70s	443.270	1.000	472.116	0.000	Percentage Correct Cards - Card Pairs Task
decade80s	173.588	1.000	184.885	0.000	Percentage Correct Cards - Card Pairs Task
Residual	9628.428	10255.000			Percentage Correct Cards - Card Pairs Task
decade60s	436.281	1.000	497.200	0.000	Total Correct Responses - Trail Making Task
decade70s	1127.092	1.000	1284.472	0.000	Total Correct Responses - Trail Making Task
decade80s	264.674	1.000	301.631	0.000	Total Correct Responses - Trail Making Task
Residual	9848.781	11224.000			Total Correct Responses - Trail Making Task
decade60s	68.835	1.000	71.251	0.000	Total Correct Responses - Paired Associates Learning Task
decade70s	126.762	1.000	131.210	0.000	Total Correct Responses - Paired Associates Learning Task
decade80s	69.528	1.000	71.968	0.000	Total Correct Responses - Paired Associates Learning Task
Residual	5450.712	5642.000			Total Correct Responses - Paired Associates Learning Task
decade60s	4.648	1.000	4.746	0.029	Total Errors - Picture Completion Task
decade70s	77.201	1.000	78.817	0.000	Total Errors - Picture Completion Task
decade80s	17.369	1.000	17.733	0.000	Total Errors - Picture Completion Task
Residual	4130.527	4217.000			Total Errors - Picture Completion Task
decade60s	1274.273	1.000	1330.730	0.000	Immediate Prospective Memory - Median Reaction Time

**decade70s**	1322.277	1.000	1380.862	0.000	Immediate Prospective Memory - Median Reaction Time
**decade80s**	587.384	1.000	613.409	0.000	Immediate Prospective Memory - Median Reaction Time
**Residual**	54631.501	57052.000			Immediate Prospective Memory - Median Reaction Time
**decade60s**	1263.330	1.000	1314.870	0.000	Delayed Prospective Memory - Median Reaction Time
**decade70s**	1171.768	1.000	1219.572	0.000	Delayed Prospective Memory - Median Reaction Time
**decade80s**	403.577	1.000	420.041	0.000	Delayed Prospective Memory - Median Reaction Time
**Residual**	52922.929	55082.000			Delayed Prospective Memory - Median Reaction Time
**decade60s**	6372.870	1.000	6704.801	0.000	Target Detection - Mean Reaction Time
**decade70s**	7090.500	1.000	7459.809	0.000	Target Detection - Mean Reaction Time
**decade80s**	1937.934	1.000	2038.871	0.000	Target Detection - Mean Reaction Time
**Residual**	223685.357	235336.000			Target Detection - Mean Reaction Time
**decade60s**	2565.018	1.000	2659.051	0.000	Emotion Discrimination - Median Reaction Time
**decade70s**	3278.146	1.000	3398.321	0.000	Emotion Discrimination - Median Reaction Time
**decade80s**	1218.383	1.000	1263.048	0.000	Emotion Discrimination - Median Reaction Time
**Residual**	151444.110	156996.000			Emotion Discrimination - Median Reaction Time
**decade60s**	4518.529	1.000	4733.309	0.000	Manipulations 2D - Median Reaction Time
**decade70s**	7053.716	1.000	7389.000	0.000	Manipulations 2D - Median Reaction Time
**decade80s**	2317.512	1.000	2427.671	0.000	Manipulations 2D - Median Reaction Time
**Residual**	223902.254	234545.000			Manipulations 2D - Median Reaction Time
**decade60s**	1133.040	1.000	1143.076	0.000	Spatial Span - Median Reaction Time
**decade70s**	1117.133	1.000	1127.028	0.000	Spatial Span - Median Reaction Time
**decade80s**	492.641	1.000	497.004	0.000	Spatial Span - Median Reaction Time
**Residual**	234351.197	236427.000			Spatial Span - Median Reaction Time
**decade60s**	4420.367	1.000	4594.929	0.000	Blocks - Median Reaction Time
**decade70s**	5778.345	1.000	6006.535	0.000	Blocks - Median Reaction Time
**decade80s**	1867.210	1.000	1940.947	0.000	Blocks - Median Reaction Time
**Residual**	231667.347	240816.000			Blocks - Median Reaction Time
**decade60s**	2855.655	1.000	2979.211	0.000	Verbal Analogies - Median Reaction Time
**decade70s**	6478.387	1.000	6758.689	0.000	Verbal Analogies - Median Reaction Time
**decade80s**	3095.415	1.000	3229.346	0.000	Verbal Analogies - Median Reaction Time
**Residual**	226660.033	236467.000			Verbal Analogies - Median Reaction Time
**decade60s**	181.416	1.000	181.778	0.000	Word Definitions - Median Reaction Time
**decade70s**	86.857	1.000	87.030	0.000	Word Definitions - Median Reaction Time
**decade80s**	3.380	1.000	3.387	0.066	Word Definitions - Median Reaction Time
**Residual**	110010.937	110230.000			Word Definitions - Median Reaction Time
**decade60s**	2453.157	1.000	2509.617	0.000	Digit Span - Median Reaction Time
**decade70s**	3314.001	1.000	3390.274	0.000	Digit Span - Median Reaction Time

**decade80s**	1219.271	1.000	1247.334	0.000	Digit Span - Median Reaction Time
**Residual**	231512.629	236841.000			Digit Span - Median Reaction Time
**decade60s**	1692.661	1.000	1723.245	0.000	Tower of London - Median Reaction Time
**decade70s**	2641.585	1.000	2689.315	0.000	Tower of London - Median Reaction Time
**decade80s**	1188.927	1.000	1210.409	0.000	Tower of London - Median Reaction Time
**Residual**	234868.231	239112.000			Tower of London - Median Reaction Time
**decade60s**	1635.377	1.000	1701.572	0.000	Four Towers - Median Reaction Time
**decade70s**	1993.853	1.000	2074.557	0.000	Four Towers - Median Reaction Time
**decade80s**	452.014	1.000	470.310	0.000	Four Towers - Median Reaction Time
**Residual**	75008.910	78045.000			Four Towers - Median Reaction Time
**decade60s**	578.826	1.000	675.717	0.000	Switching Stroop - Median Reaction Time
**decade70s**	975.461	1.000	1138.746	0.000	Switching Stroop - Median Reaction Time
**decade80s**	444.402	1.000	518.792	0.000	Switching Stroop - Median Reaction Time
**Residual**	8685.164	10139.000			Switching Stroop - Median Reaction Time
**decade60s**	347.264	1.000	372.342	0.000	Switching Stroop - Mean Incongruent Accuracy
**decade70s**	451.957	1.000	484.597	0.000	Switching Stroop - Mean Incongruent Accuracy
**decade80s**	161.593	1.000	173.263	0.000	Switching Stroop - Mean Incongruent Accuracy
**Residual**	9456.101	10139.000			Switching Stroop - Mean Incongruent Accuracy
**decade60s**	582.720	1.000	686.887	0.000	Switching Stroop - Median Incongruent Reaction Time
**decade70s**	1028.353	1.000	1212.182	0.000	Switching Stroop - Median Incongruent Reaction Time
**decade80s**	491.456	1.000	579.309	0.000	Switching Stroop - Median Incongruent Reaction Time
**Residual**	8598.014	10135.000			Switching Stroop - Median Incongruent Reaction Time
**decade60s**	241.530	1.000	251.649	0.000	Switching Stroop - Mean Switch Accuracy
**decade70s**	229.042	1.000	238.637	0.000	Switching Stroop - Mean Switch Accuracy
**decade80s**	108.131	1.000	112.661	0.000	Switching Stroop - Mean Switch Accuracy
**Residual**	9731.335	10139.000			Switching Stroop - Mean Switch Accuracy
**decade60s**	392.076	1.000	435.453	0.000	Switching Stroop - Median Switch Reaction Time
**decade70s**	615.221	1.000	683.286	0.000	Switching Stroop - Median Switch Reaction Time
**decade80s**	377.140	1.000	418.865	0.000	Switching Stroop - Median Switch Reaction Time
**Residual**	9129.010	10139.000			Switching Stroop - Median Switch Reaction Time
**decade60s**	2.350	1.000	2.349	0.125	Picture Completion - Total Time Taken
**decade70s**	0.531	1.000	0.531	0.466	Picture Completion - Total Time Taken
**decade80s**	0.001	1.000	0.001	0.974	Picture Completion - Total Time Taken
**Residual**	4218.576	4217.000			Picture Completion - Total Time Taken
**decade60s**	433.456	1.000	487.929	0.000	Card Pairs - Median Array Time
**decade70s**	834.438	1.000	939.303	0.000	Card Pairs - Median Array Time
**decade80s**	299.164	1.000	336.760	0.000	Card Pairs - Median Array Time
**Residual**	9110.122	10255.000			Card Pairs - Median Array Time

decade60s	60.094	1.000	63.104	0.000	PAL - Median Reaction Time
decade70s	223.429	1.000	234.617	0.000	PAL - Median Reaction Time
decade80s	66.371	1.000	69.695	0.000	PAL - Median Reaction Time
Residual	5372.940	5642.000			PAL - Median Reaction Time
decade60s	79.907	1.000	82.543	0.000	Simple Reaction Time - Median Reaction Time
decade70s	133.141	1.000	137.532	0.000	Simple Reaction Time - Median Reaction Time
decade80s	51.414	1.000	53.110	0.000	Simple Reaction Time - Median Reaction Time
Residual	5849.080	6042.000			Simple Reaction Time - Median Reaction Time
decade60s	290.231	1.000	324.495	0.000	Trail Making - Mean Number Reaction Time
decade70s	972.658	1.000	1087.487	0.000	Trail Making - Mean Number Reaction Time
decade80s	268.616	1.000	300.328	0.000	Trail Making - Mean Number Reaction Time
Residual	10038.843	11224.000			Trail Making - Mean Number Reaction Time
decade60s	372.313	1.000	409.193	0.000	Trail Making - Mean Letter Reaction Time
decade70s	822.759	1.000	904.259	0.000	Trail Making - Mean Letter Reaction Time
decade80s	172.095	1.000	189.142	0.000	Trail Making - Mean Letter Reaction Time
Residual	10212.391	11224.000			Trail Making - Mean Letter Reaction Time
decade60s	108.499	1.000	110.213	0.000	Trail Making - Number/Letter Cost
decade70s	126.952	1.000	128.958	0.000	Trail Making - Number/Letter Cost
decade80s	13.131	1.000	13.339	0.000	Trail Making - Number/Letter Cost
Residual	11049.462	11224.000			Trail Making - Number/Letter Cost
decade60s	10.254	1.000	10.284	0.001	Motor Control - Mean Euclidean Distance from Target
decade70s	21.021	1.000	21.083	0.000	Motor Control - Mean Euclidean Distance from Target
decade80s	1.343	1.000	1.347	0.246	Motor Control - Mean Euclidean Distance from Target
Residual	7506.890	7529.000			Motor Control - Mean Euclidean Distance from Target

Supplementary Table 23. ANOVA analysis for ageing – primary and secondary measures.
